# Machine-learning detection of stress severity expressed on a continuous scale using acoustic, verbal, visual, and physiological data: lessons learned

**DOI:** 10.3389/fpsyt.2025.1548287

**Published:** 2025-06-13

**Authors:** Marketa Ciharova, Khadicha Amarti, Ward van Breda, Martin J. Gevonden, Sina Ghassemi, Annet Kleiboer, Christiaan H. Vinkers, Milou S. C. Sep, Sophia Trofimova, Alexander C. Cooper, Xianhua Peng, Mieke Schulte, Eirini Karyotaki, Pim Cuijpers, Heleen Riper

**Affiliations:** ^1^ Department of Clinical, Neuro- and Developmental Psychology, Amsterdam Public Health Research Institute, Vrije Universiteit Amsterdam, Amsterdam, Netherlands; ^2^ Department of Computer Science, Vrije Universiteit Amsterdam, Amsterdam, Netherlands; ^3^ Sentimentics, Dordrecht, Netherlands; ^4^ Department of Biological Psychology, Amsterdam Public Health Research Institute, Vrije Universiteit Amsterdam, Amsterdam, Netherlands; ^5^ Department of Organizational Psychology, Vrije Universiteit Amsterdam, Amsterdam, Netherlands; ^6^ Department of Psychiatry, Amsterdam University Medical Centre, Amsterdam, Netherlands; ^7^ Mood, Anxiety, Psychosis, Stress & Sleep Program, Amsterdam Neuroscience Research Institute, Amsterdam, Netherlands; ^8^ Mental Health Program, Amsterdam Public Health Research Institute, Amsterdam, Netherlands; ^9^ GGZ InGeest, Mental Health Care Institution, Amsterdam, Netherlands; ^10^ Department of Methodology and Statistics, Tilburg School of Social and Behavioral Sciences, Tilburg University, Tilburg, Netherlands; ^11^ Center for Substance Use and Addiction Research (CESAR), Department of Psychology, Education & Child Studies, Erasmus School of Social and Behavioral Science, Erasmus University Rotterdam, Rotterdam, Netherlands; ^12^ World Health Organization (WHO) Collaborating Center for Research and Dissemination of Psychological Interventions, Vrije Universiteit Amsterdam, Amsterdam, Netherlands; ^13^ Department of Clinical Psychology, University of Amsterdam, Amsterdam, Netherlands; ^14^ International Institute for Psychotherapy, Babes-Bolyai University, Cluj-Napoca, Romania

**Keywords:** stress, machine learning, multimodal, acoustic, verbal, video, physiology

## Abstract

**Background:**

Early detection of elevated acute stress is necessary if we aim to reduce consequences associated with prolonged or recurrent stress exposure. Stress monitoring may be supported by valid and reliable machine-learning algorithms. However, investigation of algorithms detecting stress severity on a continuous scale is missing due to high demands on data quality for such analyses. Use of multimodal data, meaning data coming from multiple sources, might contribute to machine-learning stress severity detection. We aimed to detect laboratory-induced stress using multimodal data and identify challenges researchers may encounter when conducting a similar study.

**Methods:**

We conducted a preliminary exploration of performance of a machine-learning algorithm trained on multimodal data, namely visual, acoustic, verbal, and physiological features, in its ability to detect stress severity following a partially automated online version of the Trier Social Stress Test. College students (*n* = 42; *M* age = 20.79, 69% female) completed a self-reported stress visual analogue scale at five time-points: After the initial resting period (P1), during the three stress-inducing tasks (i.e., preparation for a presentation, a presentation task, and an arithmetic task, P2-4) and after a recovery period (P5). For the whole duration of the experiment, we recorded the participants’ voice and facial expressions by a video camera and measured cardiovascular and electrodermal physiology by an ambulatory monitoring system. Then, we evaluated the performance of the algorithm in detection of stress severity using 3 combinations of visual, acoustic, verbal, and physiological data collected at each of the periods of the experiment (P1-5).

**Results:**

Participants reported minimal (P1, *M* = 21.79, *SD* = 17.45) to moderate stress severity (P2, *M* = 47.95, *SD* = 15.92), depending on the period at hand. We found a very weak association between the detected and observed scores (*r^2^* = .154; *p* = .021). In our *post-hoc* analysis, we classified participants into categories of stressed and non-stressed individuals. When applying all available features (i.e., visual, acoustic, verbal, and physiological), or a combination of visual, acoustic and verbal features, performance ranged from acceptable to good, but only for the presentation task (accuracy up to.71, F1-score up to.73).

**Conclusions:**

The complexity of input features needed for machine-learning detection of stress severity based on multimodal data requires large sample sizes with wide variability of stress reactions and inputs among participants. These are difficult to recruit for laboratory setting, due to high time and effort demands on the side of both researcher and participant. Resources needed may be decreased using automatization of experimental procedures, which may, however, lead to additional technological challenges, potentially causing other recruitment setbacks. Further investigation is necessary, with the emphasis on quality ground truth, i.e., gold standard (self-report) instruments, but also outside of laboratory experiments, mainly in general populations and mental health care patients.

## 1 Introduction

Acute stress is considered an adaptive process preparing an individual for changes (1–3). However, in situations where the initial stress reaction is exaggerated (4) or where recovery from the initial stress response is delayed (5), stress may pose a strain on the organism (6). Repeated or prolonged exposure to situations causing acute stress over longer periods may lead to chronic stress, characterized by long-term alterations in the autonomic nervous system (ANS) and mental health (7). Such disruptions are a risk factor for many mental disorders and physical conditions, such as depression (8), anxiety (9), substance dependence (10), or cardiovascular diseases (11). Therefore, early detection of elevated acute stress is important for the prevention of its consequences (12).

Recently, theoretical breakthroughs in machine learning and increased computational capacity provided innovative opportunities for the detection and monitoring of stress and mental states (13, 14). Moreover, capturing biological and behavioral reactions to daily stress in the individual’s natural habitat and without extra effort, so-called passive sensing (15–17), has become easier thanks to digital devices, such as smartphones or sensory devices (18, 19). Stress detection using machine learning based on quantifiable passive-sensing data, such as electrocardiogram (ECG), facial expressions, or voice, suggests good detection potential (20–22), and has the advantage of providing real-time, objective information (23).

Most studies using passive sensing to date have detected stress dichotomously, meaning its presence or absence (24, 25), or categorically, meaning in three or more categories, such as no, low, and high stress (26, 27). Detection of stress severity on a continuous scale, rather than two or more categories, is still in its infancy. Yet, it is highly relevant for clinical practice, as it represents the nature of mental states better than categories, shows the variability among individuals and thus reflects their needs for prevention of psychopathology. Furthermore, such detection may contribute to tracking trends over time, as it captures not only simple presence or absence of the state, but also approaching or crossing over to the opposite state (28).

Thus far, very few studies have attempted to detect stress severity using machine-learning approaches based on passive-sensing data, such as heart rate or facial expressions. This may be related to barriers that need to be overcome if the detected variable, e.g., stress, is defined on a scale, rather than categorically. First, we need more detailed information about the individuals’ stress experience (29). Moreover, to acquire such detailed information, reliable and valid sensors able to distinguish subtle differences between individuals and changes over time are necessary (30). The acquired data may also be influenced by additional factors, not solely the experience of stress itself, which may hamper the interpretation. For example, an important source of variance for physiological measures, such as heart rate variability, are physical demands related to postural change, locomotion, or speech production (31). Finally, more fine-grained machine-learning approaches such as continuous stress severity detection require richer data compared to coarser approaches such as dichotomous detection (32). However, data used for machine-learning detection of mental states is often compromised by noisiness and missing information, especially in uncontrolled settings in daily life (33).

Available research on continuous stress severity detection has so far been very rare and did not provide evidence related to acute stress. (34) invited actors to mimic basic emotions, their photos were evaluated by psychologists on a scale ranging from “non-stressed” to “very stressed”, and a score on the same scale was detected by an algorithm. The relationship between the score assigned by the psychologist and the one detected by the algorithm was strong, namely *r^2^* = .98 (35). However, mimicked emotions may be exaggerated and thus more easily recognizable than experienced acute stress (36). Another study (37) investigated a more clinical research question, namely chronic stress detection. The authors’ algorithm detected severity of self-reported chronic stress using ECG data in expectant mothers well, with a strong relationship between the detected and self-reported scores (*r^2^* = .94). Nevertheless, as mentioned above, chronic stress manifests differently than acute stress in physiological reactions (38). Furthermore, encouraging results come from research into conditions related to stress, such as anxiety. For example, (39) predicted public speaking anxiety based on visual and acoustic data in participants who were presenting to a virtual audience, and who were subsequently trained on presenting skills, in order to present again, applying these newly-acquired skills. Anxiety was predicted with a strong relationship (*r^2^* = .825). Hence, the abovementioned studies may serve as a base for research into acute stress severity detection. Yet, it is obvious that they cannot provide us with sufficient evidence of performance of machine-learning algorithms in stress severity detection based on passive sensing data, and more investigation is thus necessary.

Some barriers related to detecting stress severity on a continuous scale may be overcome by using a combination of data sources, such as a camera, audio recorder, and physiological sensors. Such “multimodal data” may make continuous detection more robust. First, it is not affected as much by loss in a single modality, such as background noise in voice recording, or occlusion of the face in a video, as such loss may be complemented by other data, collected from other sources (33, 40). Second, stress is a multifaceted phenomenon influencing the natural behavior of an individual, such as their voice intonations (41) or facial expressions (42), as well as their physiology (24). Therefore, multimodal data may provide a more comprehensive and holistic input (43), enhancing thus passive sensing detection in comparison to individual modalities (44).

Multimodal stress detection using passive-sensing data have applications in public health (e.g., prevention of stress-related disorders) and clinical fields, for diagnosis and to guide treatment (33, 45). When used in real-time symptom monitoring, it may show how severity of stress decreases or increases, and thus provide actionable insights to users, such as to prompt them to try to reduce stress, for example, with relaxation or breathing exercises (46). It may also capture exaggerated stress reactions or delayed recovery from stress, potentially indicating unhealthy stress responses (4, 5). Consequently, it may contribute to the indication of when to prevent an onset of a problem, when to early intervene, help understanding treatment progress, and tailor stress management strategies (47). Moreover, multimodal passive sensing may reveal (unhealthy) stress reactions before the individual is aware of them (48). It could also help exploring everyday manifestation of stress (49), and eventually lead to the identification of stress “markers”, contributing to its distinction from other mental health concepts, such as depression or anxiety (50). Multimodal passive-sensing machine-learning detection of stress severity may, if found reliable and valid, complement other valid and reliable, but more retrospective and subjective manners of stress detection, such as self-report of stressful life events and their emotional consequences (51) and ecological momentary assessment (EMA) data (52). It may also be integrated in a stress assessment together with more resource-demanding physiological measures, such as neuroendocrine (e.g., cortisol) and inflammatory markers (53, 54). Therefore, more investigation is needed.

In the current study, we aimed to explore the potential of acute stress detection based on multimodal passive-sensing data, namely acoustic (i.e., physical properties of sound), verbal (i.e., content of speech), visual (i.e., facial expressions) and physiological (i.e., ECG, electrodermal activity, and motility). We aimed to test, in a laboratory setting, whether it could detect acute stress severity indexed as a continuous score on a self-report acute stress measure. We hypothesized that we would find a significant relationship between the self-reported scores and score detected by the algorithm. Furthermore, we aimed to inform on challenges associated with this novel field of research.

## 2 Materials and methods

The current study was conducted as part of the IT4Anxiety project (55). This project aimed to connect research institutions to small and medium enterprises (i.e., start-ups) developing digital products, aiming at the prevention and treatment of anxiety and post-traumatic stress disorder (PTSD). The goal of the project was to provide a framework for collaboration in these sectors and thus help both to access the expertise the other sector possesses. The current project originated from the collaboration between Vrije Universiteit (VU) Amsterdam, its spin-off VU-Ambulatory Monitoring Solutions (VU-AMS), which developed an ambulatory monitoring system for the measurement of autonomic nervous system (ANS) activity, and the start-up Sentimentics, focused on development of machine-learning algorithms for detection of mental states. Given the multidisciplinary nature of the current study, we provide a glossary of terms used in the current text in Appendix A.

The protocol for the current study was preregistered at the Open Science Framework (https://osf.io/w3kh6; see Appendix B for differences between the protocol and the final manuscript). The experimental procedure was approved by the Scientific and Ethical Review Board of the Vrije Universiteit Amsterdam (VCWE, protocol number: VCWE-2022-110).

### 2.1 Sample size calculation

Currently, no definitive guidelines exist for power calculation in mental state detection studies. Previous studies recommended to recruit at least 100 participants to achieve satisfactory sensitivity when training and testing a prediction algorithm (56, 57). Thus, we also aimed to recruit 100 participants.

### 2.2 Participants

Participants were students of the VU Amsterdam, the Netherlands. They were informed about the study and recruited using SONA, a system for students who want to participate in experiments to gain study credits. Any Dutch-speaking student older than 18 years who provided informed consent was eligible for participation. No other exclusion criteria were applied.

### 2.3 Design and procedure

**Figure 1:**
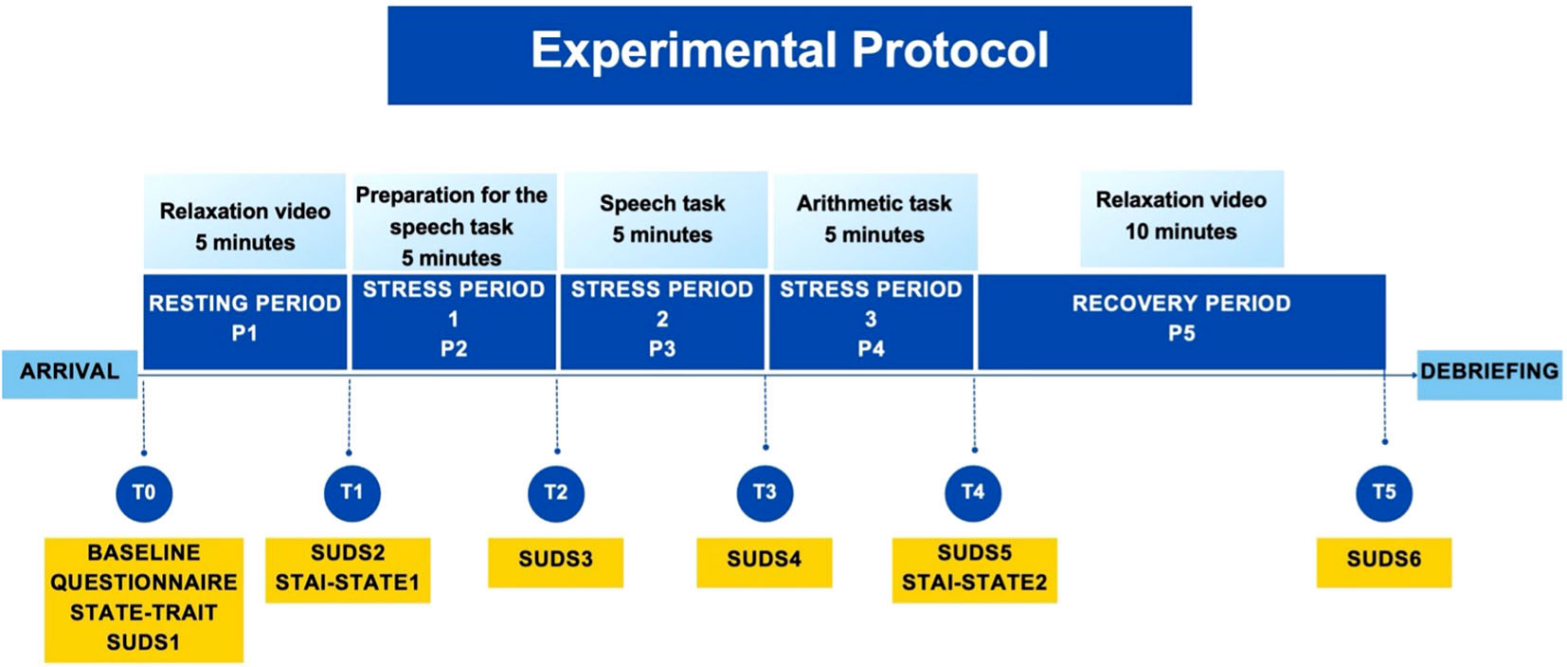
Overview of the experimental protocol.

The participants took part in a laboratory experiment. They were informed that the experiment investigated the relationship between emotions and physical reactions. Upon their arrival to the laboratory, the experimenter welcomed the participants, who then signed the informed consent. They learned that their participation was voluntary, they could withdraw at any time, and how the data would be handled. The information letter and the informed consent were provided already when the participant signed up for the study, allowing thus sufficient time to the participant to reflect on their participation. After signing the informed consent, the participant was invited to sit in a cubicle, equipped with a computer, on which the whole experiment was completed. A graphic overview of the experimental protocol can be found in **Figure 1**, while a table with a point-by-point description of all experimental periods and pictures of the solutions used is in Appendix C.

The experiment covered the following periods (P0 – P5) of the Trier Social Stress Test (TSST) (58, 59): Welcoming of the participant to the laboratory and baseline questionnaire assessment (P0), resting period (P1), preparation for presentation (P2), presentation (P3), arithmetic task (P4), and recovery period (P5). Self-report assessments (T0 - T5) took place after each period (i.e., for example, assessment T1 took place after period P1). The experimenter was not present in periods P1, P2, and P5, but he or she was present in periods P3 and P4, when joining an online videocall from another part of the laboratory (see explanation for periods P2 – P4). Audio, video, and physiology of the participant were recorded during the whole duration of the experiment.

P1 (5 minutes) served to allow the participants’ physiological and emotional responses to stabilize (60). Five minutes were chosen as they are needed for the heart rate to return to a normal level after stress (61), and longer periods were considered frustrating by participants of our pilot study. The TSST instructions do not specify a validated activity for the resting period of TSST, only suggest emotionally neutral activities or reading. To ensure participants’ focus on the screen and to uniformly deliver the resting period activity, we decided to show a video of islands, which was found to be relaxing in a previous stress-inducing laboratory experiment (62).

In P2-P4, the stress-inducing periods of TSST was administered. This was done in its validated, online version, over a Zoom™ (version 5.16.2) call (63), together with the experimenter. TSST requires a committee of at least 2 interviewers, other than the experimenter, to be present (60). Therefore, also 2 interviewers seemingly joined the call. However, to decrease resource demands, the 2 interviewers were pre-recorded, an approach which increased stress in participants of previous similar studies (64–68). Participants were not informed that the interviewers were pre-recorded. The interviewers were one male and one female professional actor (60, 63) who provided scripted instructions to the participant throughout the TSST. The role of the experimenter was to answer questions of the participant, if there were any, create additional stress in case the participants discovered the real nature of the interviewers, and to interact with the pre-recorded interviewers in a way that suggested that they were attending live (69). The script of the TSST followed its validated protocol as well as its online version (58, 59, 63), in the Dutch translation used in previous research (68, 70).

In P2 (preparation for presentation; 5 minutes), the participant received instructions to prepare for P3, in which they had to deliver a presentation about their strengths and weaknesses as part of an interview for their ideal job. They were informed that their performance would be recorded and analyzed. They could use a notepad, which opened for the duration of P2, but which closed as soon as P2 finished. For this period, the interviewers and the experimenter switched their cameras off, and the participant was informed that they were not being watched. In P3 (presentation; 5 minutes), the participant had to deliver their presentation. The interviewers and the experimenter appeared on the screen, now dressed in lab coats. If the participant stopped speaking for more than 20 seconds, the experimenter prompted them to continue speaking. In P4 (arithmetic task; 5 minutes), the participant was instructed to subtract 13 from 1022. If they made a mistake, the experimenter asked the participant to start over from 1022. If the participant stopped speaking for more than 20 seconds, the experimenter again prompted them to continue. Finally, before P5 (10 minutes; recovery period), the video call ended, and the participant watched another relaxing video of islands.

After P5, participants were debriefed. They were informed that no analysis of their speech or math performance was conducted, that the tasks were difficult and did not reflect on the participants’ aptitude or abilities.

### 2.4 Materials

#### 2.4.1 Baseline characteristics

Participants were asked about their socio-demographic characteristics (for example, age, gender, and level of education) as well as features potentially influencing stress reactions (e.g., use of medication and relaxation techniques, or mental and physical disorders).

#### 2.4.2 Stress

A visual analogue scale (VAS) called Subjective Units of Distress Scale (SUDS) (71) was used to assess stress at T0 – T5 (i.e., before and after P1-P5). VAS has been chosen as the main outcome of the current study, as it is the recommended and validated stress measure in TSST (58, 59). It is also more feasible for repeated administration than other, more comprehensive stress measures, as it consists of only one question (“How do you feel according to the following scale?”), which can be answered on an 11-point scale, ranging from “0” (“Totally relaxed”) to “100” (“Highest distress/fear/anxiety/discomfort that you have ever felt”). The score may be further categorized into “noticeable, but not bothersome anxiety” (SUDS > 25), “bothersome anxiety” (SUDS > 50), and “very bothersome anxiety” (SUDS > 75) (72, 73). It has good psychometric properties, for example concurrent validity with stress measures (Spearman *rho* = .50, p <.001) (74) and clinician’s rating of general functioning (*r* = -.44, *p* <.001) (75). The SUDS has been translated into Dutch using a back-translation method recommended by the World Health Organization (76).

#### 2.4.3 State and trait anxiety

State and Trait Anxiety Inventory-Alternate Form (STAI-A) (77) was administered in its Dutch version (78). STAI-A is a shorter version of the State and Trait Anxiety Inventory (STAI) (79) and introduces 10 questions focused on state and 10 on trait anxiety. The total scores can then be categorized according to respective norms into low (below the 25. percentile), high (above the 75. percentile), and normal levels of anxiety (80). STAI-A has been found to be a reliable instrument (*α* = .80), with results equivalent to the full version of the STAI (77). The Trait subscale was administered at T0, the first State subscale at T1, and the second State subscale at T4.

#### 2.4.4 Audio and video

A video camera (Canon, Legria HF R86, 10 ADP, 1920x1080, 50 frames per second, 35 mbps, Dolby Digital 2ch) was used to record audio and video data. It was located below the computer screen on which the participant followed the experiment, and it was focused on the participant from their shoulders up.

##### 2.4.4.1 Visual features

Fifty-seven visual features were extracted: 17 action units (meaning the fundamental actions of individual muscles of the face (81)), 32 parameters of the Point Distribution Model (PDM) capturing facial landmark shape variations and changes (82), and 8 parameters of the eye gaze direction. For a detailed explanation of these parameters, please see the original study on OpenFace 2.0, the software used in the current analysis (83). Visual features were extracted for all periods of the experiment, i.e., P1 – P5. As the features had to be aggregated across each of these periods, the following parameters were created for each feature: mean (*M*), median (*Med*), standard deviation (*SD*), minimum (*MIN*), maximum (*MAX*), skewness, kurtosis, slope, offset, and curvature of fitting a second-degree polynomial to capture temporal patterns. As a result, 570 parameters were extracted for visual features.

##### 2.4.4.2 Acoustic features

There were 79 acoustic features related to pitch intensity and frequency, formant frequency and bandwidth, harmonic to noise ratio, zero crossing rate, Mel Frequency Cepstral Coefficients (MFCC), Linear Frequency Cepstral Coefficients (LFCC), and other parameters which capture spectral properties of audio (see Appendix D for explanations). These features were estimated for P3 and 4, i.e., the two periods in which the participants spoke. We used the same 10 aggregation methods applied to the visual features (see above), resulting in a total of 790 audio features.

##### 2.4.4.3 Verbal features

The experiment took place in the Dutch language. The content of participants’ speech was automatically transcribed using Speech-to-Text API with a guaranteed 85% accuracy (84) and manually corrected for mistakes by a researcher, a methodology previously recommended (85, 86). The transcripts were then processed: punctuations and stop words were removed, and words were lemmatized (i.e., returned to their dictionary form). Verbal features were extracted using Bag of Words method, where the most common words are counted and used as verbal features. To encourage generalization, a word was considered if it appeared in at least three transcripts. Verbal features were extracted for P3 (64 words) and P4 (22 words), in which participants had to speak.

#### 2.4.5 Physiological measures

Physiological data were recorded using the VU-AMS (version 5-wire 5fs) (87), a lightweight, portable ambulatory monitoring system for the non-invasive measurement of ANS activity. It records a continuous electro- and impedance cardiogram (ECG/ICG), electrodermal activity (EDA), and accelerometry signal. The ECG and ICG signal was acquired at 1000 Hz using 5 ECG electrodes (Kendall H98SG, Medtronic, Eindhoven, Netherlands) located on the chest and back of the participant (87, 88). The EDA signal was measured at 10 Hz using an EDA electrode (Type 10-W 55 GS, Movisens GmbH, Karlsruhe, Germany) placed on the thenar eminence of the non-dominant hand, and an ECG electrode on the lower arm. Participant movement was detected through a triaxial accelerometer placed on the table, so that postural changes would shift the sensor through pull on the cables but prevent loss of signal due to fall or disconnection of the device.

##### 2.4.5.1 Physiological features

First, data were preprocessed and the signals visually inspected for noise, ectopic beats, and misplacement of R-peaks in the ECG and B- and X-points in the ICG. Then each feature was calculated using Vrije Universiteit Data Acquisition and Management Software (Version 5.4.20) for every period of interest, i.e., P1 – P5. Similarly to the visual features, the features had to be aggregated across each of these periods. Thus, where applicable, *M*, *MIN*, *MAX* and/or *SD* were used. In total, 31 parameters were extracted for each period. A more detailed overview and explanation of physiological features used in the current study can be found in Appendix E.

### 2.5 Analyses

Descriptive analyses were conducted in IBM SPSS Statistics (version 27). The model fitting and prediction were performed using Python 3.6 for programming, using the following libraries: OpenFace 2.0 facial behavior analysis toolkit (83), and librosa (89) for the extraction of the visual, and acoustic features, respectively. Verbal features were extracted, and regression, classification and cross-validation were conducted in SKlearn (90).

All visual, acoustic, verbal, and physiological features for each participant were concatenated and fed into the model to detect stress severity at each of the measurement time points (T1 – T5). Data from each period were used to detect stress severity at the following time point, for example, data collected during period P1 (i.e., between the time points T0 and T1) were used to detect stress severity as assessed by the SUDS at T1. Separate analyses for each period are recommended because the TSST structure is divided into five distinct phases. Each phase is designed to elicit unique stress responses that capture the temporal dynamics of stress (61).

To assess the additive predictive power of each set of features, three different combinations of groups of features were used (1): visual + physiological features (2), visual + acoustic + verbal features, and (5) visual + acoustic + verbal + physiological features. A leave-one-out cross-validation was applied to the model, where in each iteration, data of one participant were placed aside, the rest of the data was used for the training of the model, and the resulting model was used for prediction of the stress level in the left-out participant (91). A feature selection was used, where for each iteration, 30 features with the most significant correlation with the self-reported stress severity were used in the model (meaning that not all extracted features were used for final stress detection). To avoid data leakage and ensure reliability of the results, this was also done in the training set of each iteration of cross-fold validation, meaning that different features could be selected in different iterations (92). The aim of feature selection was to prevent overfitting due to the large number of features and the small number of participants (93). Subsequently, stress levels were predicted as a continuous variable, meaning predicting a specific score on the SUDS scale, using Bayesian Ridge regression, as this linear model with a minimum number of hyper-parameters suits small datasets (94, 95). Details of the used algorithm are described in (96). This algorithm has been shown to outperform other state-of-the-art methods, and to enable feature extraction process and a novel temporal aggregation method with no initial data annotations. Additionally, it has been proposed as suitable for small sample sizes (96).

The coefficient of determination (*R^2^*) was calculated, where values can be interpreted as strong (>.75), moderate (.5 -.75), weak (.25 -.5), or no (<.25) relationship between the predicted and the observed scores (35). We also calculated correlations (Pearson’s r) between all features and stress at T1 through T5 to provide per-parameter descriptive information to the reader. Pearson’s *r* may be interpreted as small, medium, or large effect for *r* = .10,.30, and.50, respectively (97).

## 3 Results

### 3.1 Participant characteristics

According to our preregistered protocol, we aimed at recruiting 100 participants. However, we failed due to restrictions caused the COVID-19 pandemic, which postponed the recruitment and thus caused time constraints. Eventually, recruitment was conducted between March and June 2023.

Forty-six participants joined the study, but due to technical problems in the experiment, data for only 42 participants were available. Data for some participants were only partially available, due to software issues causing some of the parts of the experiment not to start or close prematurely. Subsequently, data for 28–31 participants were available for each period (**Table 1**). All participants were bachelor students, most were female (69%), born in the Netherlands (95%), and did a major in psychology (88%) (**Table 2**).

**Table 1:** Stress severity as a continuous variable: Detection of stress based on different combinations of features (Regression).

Period	r^2^	r	p
Visual + Physiological			
Period 1	-1.452	-.325	.080
Period 2	-0.447	-.084	.652
Period 3	-0.374	.075	.689
Period 4	-0.667	-.360	.047
Period 5	-1.889	-.212	.280
Visual + Acoustic + Verbal			
Period 1	-1.395	-.296	.112
Period 2	-1.544	-.196	.292
Period 3	0.051	.324	.075
Period 4	-1.601	-.597	.000
Period 5	-1.891	-.204	.297
Visual + Acoustic + Verbal + Physiological			
Period 1	-1.452	-.325	.080
Period 2	-0.447	-.084	.652
Period 3	**0.154**	**.414**	**.021**
Period 4	-1.297	-.436	.014
Period 5	-1.889	-.212	.280

p, p-value; r, Pearson’s r; r2, Coefficient of determination.

Numbers of participants with available data: Period 1: n = 30, Period 2-4: n = 31, Period 5: n = 28.

**Table 2:** Socio-demographic characteristics and scores on self-reported scales, *n* = 42.

Characteristics and Scores	*n* (%)
Female	29 (69%)
Born in the Netherlands	40 (95%)
	*M (SD)*
Age (years)	20.79 (2.12)
STAI-A-Trait (P0)	19.69 (5.73)
STAI-A-State (P1)	16.93 (4.18)
STAI-A-State (P4)	21.08 (4.53)
SUDS (P0)	28.10 (17.14)
SUDS (P1)	21.79 (17.45)
SUDS (P2)	47.95 (15.92)
SUDS (P3)	43.95 (17.64)
SUDS (P4)	36.05 (18.09)
SUDS (P5)	22.00 (17.62)

*Abbreviations (alphabetical).* M, mean; n, number of participants; SD, standard deviation; STAI-A, State and Trait Anxiety Inventory-Alternate Form; SUDS, Subjective Units of Distress Scale; T0-T5, Time points 0 – 5.

The average scores on STAI-A-Trait and -State before and after the TSST fell into the category of normal anxiety (**Table 2**). The SUDS ratings, on average, ranged from minimal distress at P1 (i.e., during the resting period) to moderate distress at P2 (i.e., during the preparation for presentation). There was a visible increase in stress in P2, when the participants entered the video call, which was then slightly decreasing over the stress periods (P3-4) and eventually decreased again in the recovery period (P5).

### 3.2 Predicting stress as a continuous variable (regression)

When stress was measured on a continuous scale, the algorithm did not predict stress severity well in most combinations of features and time periods (**Table 1**). However, it predicted stress severity at P3 when all features, i.e., visual, acoustic, verbal, and physiological, were used (*r^2^* = .154; *p* = .021). Still, it points to a very weak association between the predicted and observed scores.

### 3.3 *Post-hoc* analysis: predicting stress as a dichotomous variable (classification)

Since the prediction model could not fully detect patterns in the features, we also conducted a *post-hoc* analysis to simplify the modeling process (98). In this analysis, we treated stress as a dichotomous variable, meaning a stressed (i.e., “bothersome anxiety”, SUDS > 50) and a non-stressed group (SUDS < 50) (72, 73). We did so for P2 and P3, where higher variability of stress among participants was present. Logistic regression was conducted, and F1-measure and accuracy were computed. F1-measure ranges between 0 and 1, and its values may be evaluated as very good (>.9), good (.8 -.9), acceptable (.5 -.8), or poor (<.5) (99), where values approaching to 1 express the best trade-off between precision and sensitivity (100). Levels of accuracy also range between 0 and 1, and have been categorized as very good (>.9), good (.7 -.9), acceptable (.6 -.7), or poor (<.6) (101).

Classification results are shown in **Table 3**, the confusion matrix (i.e., table reporting numbers of true positives, false negatives, false positives and true negatives) is in **Table 4**. The algorithm reached acceptable to good performance in two cases at P3: when all but physiological (accuracy = .710, F1-score = .727) or all features (accuracy = .677, F1-score = .688) were combined.

**Table 3:** Stress presence and absence as a dichotomous variable: Detection of stress based on different combinations of features, *n* = 31 (Classification).

Period	Accuracy	F1-score
Visual + Physiological		
Period 2	0.452	0.564
Period 3	0.516	0.571
Period 4	0.419	0.100
Visual + Acoustic + Verbal		
Period 2	0.355	0.474
Period 3	**0.710**	**0.727**
Period 4	0.355	0.000
Visual + Acoustic + Verbal + Physiological		
Period 2	0.452	0.564
Period 3	**0.677**	**0.688**
Period 4	0.419	0.100

Results considered acceptable are in bold.

**Table 4:** Confusion matrix for the dichotomous (i.e., classification) detection based on different combinations of features.

Reported/predicted	Period 2		Period 3		Period 4	
	Not stressed (predicted)	Stressed (predicted)	Not stressed (predicted)	Stressed (predicted)	Not stressed (predicted)	Stressed (predicted)
Visual + Physiological						
Not stressed (reported)	3	8	6	8	12	8
Stressed (reported)	9	11	7	10	10	1
Visual + Acoustic + Verbal						
Not stressed (reported)	2	9	10	4	11	9
Stressed (reported)	11	9	5	12	11	0
Visual + Acoustic + Verbal + Physiological						
Not stressed (reported)	3	8	10	4	12	8
Stressed (reported)	9	11	6	11	10	1

### 3.4 Correlations between visual, audio, and physiological features, and stress

Appendix F shows correlations between tested features and stress. Correlations for visual features with stress at P1-P5 can be found in **Supplementary Table S1**, correlations between acoustic features with stress at P3 and 4 are in **Supplementary Table S2**, verbal features for P3 and 4 are shown in **Supplementary Tables S3** and **Supplementary Tables S4**, respectively. **Supplementary Table S5** demonstrates correlations between physiological features and stress in P1 through 5.

## 4 Discussion

This study explored the potential of using multimodal data collected in a laboratory setting through passive sensing for machine-learning detection of self-reported acute stress severity expressed on a continuous scale. The best algorithm performance was a weak relationship between the detected and observed score (*r^2^* = .154), when features of all modalities, meaning visual, acoustic, verbal, and physiological, were included, in the period when participants were giving a presentation. We also conducted a *post-hoc* analysis, in which we classified participants as stressed and non-stressed. The performance of the algorithm was then acceptable to good (accuracy up to.71) during the presentation period when using all or almost all modalities. All significant detection of stress thus took place during the presentation period, when data from all modalities could be collected. Moreover, during this period, the verbal modality provided a high variability of possible input, depending on the words the participant used, as opposed to the arithmetic task, where the participants’ speech was restricted. Moreover, we could see that combination of data from multiple modalities showed better performance than combinations of fewer data sources. In the continuous prediction, physiological features, when added to the detection, helped improve algorithm performance. However, this difference was not apparent in the *post-hoc* analysis (i.e., dichotomous classification) anymore.

The current study cannot be compared to previous research, as it is the first exploring the potential of continuous detection of stress by machine-learning models based on multimodal data. Evidence from the two studies attempting to detect mimicked or chronic stress (34, 37), as well as severity of conditions related to stress, such as anxiety or post-traumatic stress disorder (PTSD), suggests that moderate to strong relationship between detected and observed scores is possible even if stress is expressed on a continuous scale (39, 43, 102, 103). In the current study, we did not find such a strong relationship, which may be caused by a different outcome variable of interest than in these previous studies. Moreover, most of these studies focused on anxiety or PTSD in the community (102, 103) or clinical settings (43), thus further hampering the comparison.

Comparison between our results and previous research may thus be only based on the *post-hoc* dichotomous prediction, which we conducted when it became clear that our sample size prevented the algorithm from recognizing patterns in the data. Some studies used multimodal data for dichotomous detection of acute stress induced in laboratory experiments (62) used a combination of video (capturing movements, e.g., symmetry, and behavior, e.g., gestures), ECG, EDA, and foot trembling, for detection with very good performance (i.e., accuracy of up to 1) (101). 104 reported good performance (accuracy = .85) (101) when applying features related to voice, facial expressions, and ECG data. That is slightly better than detection in our *post-hoc* analysis, where we reached acceptable to good prediction (i.e., accuracy up to.71). The reasons for this discrepancy may be that statistical power in both studies was higher than ours due to bigger sample sizes. Even though neither of these studies recruited more participants than our study did (*n* = 21, and *n* = 20, respectively), in both studies, data from all conditions were merged in the analysis, meaning that one participant contributed to more data points, resulting thus in larger datasets (*n* = 108, and *n* = 1271, respectively). Their approach, however, does not express the temporal dynamics of stress which we aimed to reflect in our analysis by analyzing each period of the experiment separately (61).

From a methodological perspective, our study is innovative and explores potential improvements to laboratory experiment methods. To the best of our knowledge, it is the first study incorporating pre-recorded interviewers into a validated Zoom™ version of the TSST protocol (63). Although the interviewers were pre-recorded, reported stress among participants increased when they entered the video call. Thus, this method might be a viable solution to diminish resource demands of the online TSST in future studies (65, 67). In addition, the resting and recovery periods are not specified nor standardized in the TSST protocol (58, 59). We aimed at standardization by applying a method previously successfully inducing relaxation in another study applying a laboratory stress experiment (62). Finally, the results were based on long periods of time (i.e., 5–10 minutes), allowing thus more reliable estimates of stress severity, as longer recordings were suggested for precise stress detection (105).

Limitations need to be acknowledged as well. First and foremost, the combination of a small sample with the complexity of input features limited our predictive power. Larger samples are thus required to build more robust algorithms. Nevertheless, to handle this problem, we applied simpler machine-learning models, and we used feature selection to choose 30 features with the most significant correlation with the self-reported stress severity to balance it with the number of participants in our dataset. Additionally, to validate the performance, we performed a leave-one-out cross-validation. Furthermore, we added a *post-hoc* analysis, formulating the research question as a dichotomous problem. In this analysis, the algorithm’s performance was good, meaning that predictive power was present. Second, the generalizability of the results to natural behavior, ecologically valid or clinical contexts may be hampered, as the current study took place in a controlled, laboratory setting. Similarly, the results are limited to our sample, which mostly consisted of Netherlands-born female psychology students, thus potentially introducing selection bias. Third, the observed and detected stress in the current study was only artificially induced. Hence, caution must be taken when drawing conclusions about naturally occurring stress. Fourth, one of the included modalities were physiological data, which were observed not to have a strong relationship with self-reported stress (106). However, we deemed it still of importance to include it. While physiological data are objective markers of stress, capturing the body’s ANS activity, self-reported stress is of a subjective nature, showing stress awareness. These two pieces of information, also together with data from other modalities, such as video and audio, thus provide a more holistic picture of the individual’s stress experience. We also conducted an analysis excluding physiological measures, which did not affect the detection of stress in both main (continuous) and *post-hoc* (dichotomous) analysis. In the continuous analysis, it also seemed that physiological features contributed to the algorithm’s performance, as only combination of all modalities provided significant detection. However, this improvement disappeared in the *post-hoc* analysis, rendering thus physiological features to be the best candidates for exclusion. Finally, we used a unified relaxation method during resting and recovery periods, we thus did not let the participants simply sit in the waiting room as the protocol suggests. We also applied pre-recorded instead of in-person interviewers to provide instructions. Furthermore, we asked the participants to be seated during the whole duration of the experiment, as we needed to avoid noisiness or loss of data due to unnecessary movement, which is not uncommon in virtual versions of the TSST. However, these facts should be taken into account when interpreting our results (107).

Future research should focus on acquiring sufficient training data for multimodal algorithms, with a good “ground truth”, meaning a gold standard measure, such as a diagnostic interview or a validated self-report instrument (36), and a lot of variability (108). Currently, there has been a sharp increase in machine-learning algorithm development, and these methods keep evolving fast (109). Acquiring sufficient, rich training data may thus also help applying newer algorithms with better performance, something that was not feasible in the current study. Further laboratory experiments should include participants coming from both general and clinical populations. Then, it will be beneficial to relocate the research community’s attention towards real-life situations, such as daily stress monitoring in general populations, workplace settings or mental health care patients. Moreover, attitudes of professionals and patients towards usage of machine-learning algorithms for mental health will have to be explored through both qualitative and quantitative (user) research, as adoption of new technologies may be accompanied with reluctance, e.g., due to privacy issues (110). Finally, it is crucial to consider ethical aspects of research on machine-learning monitoring of mental states based on natural behavior, as such results may be, in extreme cases, used for privacy and human right violations (111).

Even if some results seem promising, we are currently very far from public health or clinical implementation. Only after thorough investigation, it may be explored whether validated stress detection measures may be complemented by real-time machine-learning algorithms based on multimodal data acquired through passive sensing. Low threshold and self-monitoring of daily stress in general populations may then contribute to prevention or early intervention efforts, even in situations where the individual is not yet ready to verbalize their experience. Successful measurement of severity may contribute to measurement of stress dynamics over time as well. As stress is a transdiagnostic concept, measuring of its severity, and especially delayed recovery from stress reactions, may help us detect the right moment for early intervention for potential consequences of chronic stress, such as burnout or depression. Later on, such detection may become an integrated component of digital interventions where real-time assessment is necessary or highly desirable, such as in just-in-time adaptive interventions for mental disorders (112, 113).

## Data Availability

The raw data supporting the conclusions of this article will be made available by the authors, without undue reservation.

## Author’s note

The multimodal algorithm used in this study was originally developed in a separate project involving Sentimentics, VU University Amsterdam, and other collaborators, under the leadership of Prof. Dr. R. de Vries, VU University Amsterdam.

## References

[1] Selye H. The stress syndrome. AJN Am J Nursing. (1965) 65:97–9. 5175587

[2] Hoffman-Goetz L Pedersen BK. Exercise and the immune system: a model of the stress response? Immunol Today. (1994) 15:382–7. 10.1016/0167-5699(94)90177-5 7916952

[3] McEwen B. Stress: Homeostasis, rheostasis, reactive scope, allostasis and allostatic load. In: Reference module in neurosciene and biobehavioral psychology. Amsterdam, the Netherlands: Elsevier (2017).

[4] Turner AI Smyth N Hall SJ Torres SJ Hussein M Jayasinghe SU. Psychological stress reactivity and future health and disease outcomes: A systematic review of prospective evidence. Psychoneuroendocrinology. (2020) 114:104599. doi: 10.1016/j.psyneuen.2020.104599 32045797

[5] De Calheiros Velozo J Lafit G Viechtbauer W van Amelsvoort T Schruers K Marcelis M. Delayed affective recovery to daily-life stressors signals a risk for depression. J Affect Disord. (2023) 320:499–506. doi: 10.1016/j.jad.2022.09.136 36208689

[6] Nesse RM Young EA. Evolutionary origins and functions of the stress response. Encyclopedia stress. (2000) 2:79–84. doi: 10.1016/B978-012373947-6.00150-1

[7] Rohleder N. Stress and inflammation–The need to address the gap in the transition between acute and chronic stress effects. Psychoneuroendocrinology. (2019) 105:164–71. doi: 10.1016/j.psyneuen.2019.02.021 30826163

[8] Hammen CL. Stress and depression: old questions, new approaches. Curr Opin Psychol. (2015) 4:80–5. doi: 10.1016/j.copsyc.2014.12.024

[9] Daviu N Bruchas MR Moghaddam B Sandi C Beyeler A. Neurobiological links between stress and anxiety. Neurobiol stress. (2019) 11:100191. doi: 10.1016/j.ynstr.2019.100191 31467945 PMC6712367

[10] Andersen SL. Stress, sensitive periods, and substance abuse. Neurobiol stress. (2019) 10:100140. doi: 10.1016/j.ynstr.2018.100140 30569003 PMC6288983

[11] Kivimäki M Steptoe A. Effects of stress on the development and progression of cardiovascular disease. Nat Rev Cardiol. (2018) 15:215–29. doi: 10.1038/nrcardio.2017.189 29213140

[12] Walker AJ Kim Y Price JB Kale RP McGillivray JA Berk M. Stress, inflammation, and cellular vulnerability during early stages of affective disorders: biomarker strategies and opportunities for prevention and intervention. Front Psychiatry. (2014) 5:34. doi: 10.3389/fpsyt.2014.00034 24782789 PMC3988376

[13] Thompson NC Greenewald K Lee K Manso GF. The computational limits of deep learning. In: arXiv preprint arXiv Ithaca, New York, United States: Cornell University (2020). p. 200705558.

[14] Xu Z Sun J. Model-driven deep-learning. Natl Sci Review. (2018) 5:22–4. doi: 10.1093/nsr/nwx099

[15] Torous J Kiang MV Lorme J Onnela J-P. New tools for new research in psychiatry: a scalable and customizable platform to empower data driven smartphone research. JMIR Ment Health. (2016) 3:e5165. doi: 10.2196/mental.5165 PMC4873624 27150677

[16] Oudin A Maatoug R Bourla A Ferreri F Bonnot O Millet B. Digital phenotyping: Data-driven psychiatry to redefine mental health. J Med Internet Res. (2023) 25:e44502. doi: 10.2196/44502 37792430 PMC10585447

[17] Cornet VP Holden RJ. Systematic review of smartphone-based passive sensing for health and wellbeing. J Biomed informatics. (2018) 77:120–32. doi: 10.1016/j.jbi.2017.12.008 PMC5793918 29248628

[18] Choi A Ooi A Lottridge D. Digital phenotyping for stress, anxiety, and mild depression: systematic literature review. JMIR mHealth uHealth. (2024) 12:e40689. doi: 10.2196/40689 38780995 PMC11157179

[19] Mirjafari S Masaba K Grover T Wang W Audia P Campbell AT. Differentiating higher and lower job performers in the workplace using mobile sensing. Proc ACM Interactive Mobile Wearable Ubiquitous Technologies. (2019) 3:1–24. doi: 10.1145/3328908

[20] Giannakakis G Trivizakis E Tsiknakis M Marias K. (2019). A novel multi-kernel 1D convolutional neural network for stress recognition from ECG, in: 2019 8th International Conference on Affective Computing and Intelligent Interaction Workshops and Demos (ACIIW), Piscataway, New Jersey, United States. IEEE.

[21] Gavrilescu M Vizireanu N. Predicting depression, anxiety, and stress levels from videos using the facial action coding system. Sensors. (2019) 19:3693. doi: 10.3390/s19173693 31450687 PMC6749518

[22] Lech M He L. Stress and emotion recognition using acoustic speech analysis. Ment Health Informatics: Springer;. (2013) p:163–84. doi: 10.1007/978-3-642-38550-6_9

[23] Jongs N. Passive digital phenotyping: Objective quantification of human behaviour through smartphones. Groningen, the Netherlands: University of Groningen (2021). doi: 10.1007/978-3-642-38550-6_9

[24] Arsalan A Majid M. Human stress classification during public speaking using physiological signals. Comput Biol medicine. (2021) 133:104377. doi: 10.1016/j.compbiomed.2021.104377 33866254

[25] Zhu L Spachos P Ng PC Yu Y Wang Y Plataniotis K. Stress detection through wrist-based electrodermal activity monitoring and machine learning. IEEE J Biomed Health Informatics. (2023) 27:2155–65. doi: 10.1109/JBHI.2023.3239305 37022004

[26] Gjoreski M Gjoreski H Luštrek M Gams M. (2016). Continuous stress detection using a wrist device: in laboratory and real life. In Proceedings of the 2016 ACM International Joint Conference on Pervasive and Ubiquitous Computing: Adjunct. Heidelberg, Germany: Association for Computing Machinery. pp. 1185–1193.

[27] Fazeli S Levine L Beikzadeh M Mirzasoleiman B Zadeh B Peris T. (2023). A self-supervised framework for improved data-driven monitoring of stress via multi-modal passive sensing, in: 2023 IEEE International Conference on Digital Health (ICDH). IEEE.

[28] Lahey BB Tiemeier H Krueger RF. Seven reasons why binary diagnostic categories should be replaced with empirically sounder and less stigmatizing dimensions. JCPP advances. (2022) 2:e12108. doi: 10.1002/jcv2.12108 37431412 PMC10242872

[29] Kumar A Batut B. Statistics and machine learning / machine learning: Classification and regression / hands-on: Machine learning: Classification and regression. Galaxy Training Network (2024). Available online at: https://training.galaxyproject.org/training-material/topics/statistics/tutorials/classification_regression/tutorial.html (Accessed April 25, 2008).

[30] Zou Z Ergan S. Evaluating the effectiveness of biometric sensors and their signal features for classifying human experience in virtual environments. Advanced Eng Informatics. (2021) 49:101358. doi: 10.1016/j.aei.2021.101358

[31] Damoun N Amekran Y Taiek N El Hangouche AJ. Heart rate variability measurement and influencing factors: Towards the standardization of methodology. Global Cardiol Sci Practice. (2024) 2024:e202435. doi: 10.21542/gcsp.2024.35 PMC11439429 39351472

[32] Zhou L Pan S Wang J Vasilakos AV. Machine learning on big data: Opportunities and challenges. Neurocomputing. (2017) 237:350–61. doi: 10.1016/j.neucom.2017.01.026

[33] Mentis A-FA Lee D Roussos P. Applications of artificial intelligence– machine learning for detection of stress: a critical overview. Mol Psychiatry. (2023) 29:1–13. doi: 10.1038/s41380-023-02047-6 37020048

[34] Das S Yamada K. Evaluating instantaneous psychological stress from emotional composition of a facial expression. J Advanced Comput Intell Intelligent Informatics. (2013) 17:480–92. doi: 10.20965/jaciii.2013.p0480

[35] Zach. What is considered to be a “Strong” Correlation? Statology. (2020). Available online at: https://www.statology.org/what-is-a-strong-correlation/.

[36] Ciharova M Amarti K van Breda W Peng X Lorente-Català R Funk B. Use of machine-learning algorithms based on text, audio and video data in the prediction of anxiety and post-traumatic stress in general and clinical populations: A systematic review. Biol Psychiatry. (2024) 96:519–31. doi: 10.1016/j.biopsych.2024.06.002 38866173

[37] Sarkar P Lobmaier S Fabre B González D Mueller A Frasch MG. Detection of maternal and fetal stress from the electrocardiogram with self-supervised representation learning. Sci reports. (2021) 11:24146. doi: 10.1038/s41598-021-03376-8 PMC8683397 34921162

[38] Schubert C Lambertz M Nelesen R Bardwell W Choi J-B Dimsdale J. Effects of stress on heart rate complexity—a comparison between short-term and chronic stress. Biol Psychol. (2009) 80:325–32. doi: 10.1016/j.biopsycho.2008.11.005 PMC2653595 19100813

[39] Wörtwein T Morency L-P Scherer S. (2015). Automatic assessment and analysis of public speaking anxiety: A virtual audience case study, in: 2015 International Conference on Affective Computing and Intelligent Interaction (ACII), Piscataway, New Jersey, United States. IEEE.

[40] Ngiam J Khosla A Kim M Nam J Lee H Ng AY. (2011). Multimodal deep learning, in: Proceedings of the 28th International Conference on Machine Learning, International Conference on Machine Learning, Bellevue, Washington, United States.

[41] Pešán J Juřík V Ruzickova A Svoboda V Janousek O Nemcova A. Speech production under stress for machine learning: multimodal dataset of 79 cases and 8 signals. Sci Data. (2024) 11:1221. doi: 10.1038/s41597-024-03991-w 39532912 PMC11557825

[42] Giannakakis G Koujan MR Roussos A Marias K. Automatic stress analysis from facial videos based on deep facial action units recognition. In: Pattern analysis and applications New York City, NY, United States: Springer (2022). p. 1–15.

[43] Schultebraucks K Yadav V Shalev AY Bonanno GA Galatzer-Levy IR. Deep learning-based classification of posttraumatic stress disorder and depression following trauma utilizing visual and auditory markers of arousal and mood. psychol Medicine. (2022) 52:957–67. doi: 10.1017/S0033291720002718 32744201

[44] Salvi M Loh HW Seoni S Barua PD García S Molinari F. Multi-modality approaches for medical support systems: A systematic review of the last decade. . Inf Fusion. (2023) 102134:102134. doi: 10.1016/j.inffus.2023.102134

[45] Bolpagni M Pardini S Dianti M Gabrielli S. Personalized stress detection using biosignals from wearables: A scoping review. Sensors. (2024) 24:3221. doi: 10.3390/s24103221 38794074 PMC11126007

[46] Lazarou E Exarchos TP. Predicting stress levels using physiological data: Real-time stress prediction models utilizing wearable devices. AIMS Neurosci. (2024) 11:76–102. doi: 10.3934/Neuroscience.2024006 38988886 PMC11230864

[47] Wang L Hu Y Jiang N Yetisen AK. Biosensors for psychiatric biomarkers in mental health monitoring. Biosensors Bioelectronics. (2024) 116242:116242. doi: 10.1016/j.bios.2024.116242 38631133

[48] Yu H Sano A. (2020). Passive sensor data based future mood, health, and stress prediction: User adaptation using deep learning, in: 2020 42nd Annual International Conference of the IEEE Engineering in Medicine & Biology Society (EMBC), Piscataway, New Jersey, United States. IEEE. 10.1109/EMBC44109.2020.9176242 33019313

[49] Huckvale K Venkatesh S Christensen H. Toward clinical digital phenotyping: a timely opportunity to consider purpose, quality, and safety. NPJ digital medicine. (2019) 2:1–11. doi: 10.1038/s41746-019-0166-1 31508498 PMC6731256

[50] Wulsin LR Sagui-Henson SJ Roos LG Wang D Jenkins B Cohen BE. Stress measurement in primary care: Conceptual issues, barriers, resources, and recommendations for study. Psychosomatic medicine. (2022) 84:267–75. doi: 10.1097/PSY.0000000000001051 PMC8976751 35067657

[51] Gillham B. Developing a questionnaire. London, United Kingdom: Bloomsbury (2008).

[52] Yang YS Ryu GW Choi M. Methodological strategies for ecological momentary assessment to evaluate mood and stress in adult patients using mobile phones: systematic review. JMIR mHealth uHealth. (2019) 7:e11215. doi: 10.2196/11215 30932866 PMC6462888

[53] Kemp AH Koenig J Thayer JF Bittencourt MS Pereira AC Santos IS. Race and resting-state heart rate variability in Brazilian civil servants and the mediating effects of discrimination: an ELSA-Brasil cohort study. Psychosomatic Medicine. (2016) 78:950–8. doi: 10.1097/PSY.0000000000000359 27359180

[54] Kudielka BM Schommer NC Hellhammer DH Kirschbaum C. Acute HPA axis responses, heart rate, and mood changes to psychosocial stress (TSST) in humans at different times of day. Psychoneuroendocrinology. (2004) 29:983–92. doi: 10.1016/j.psyneuen.2003.08.009 15219648

[55] INTERREG North-West Europe. IT4Anxiety (2020). Available online at: https://vb.nweurope.eu/projects/project-search/it4anxiety-managing-anxiety-via-innovative-technologies-for-better-mental-health/ (Accessed May 31, 2025).

[56] Beleites C Neugebauer U Bocklitz T Krafft C Popp J. Sample size planning for classification models. Analytica chimica Acta. (2013) 760:25–33. doi: 10.1016/j.aca.2012.11.007 23265730

[57] Sajjadian M Lam RW Milev R Rotzinger S Frey BN Soares CN. Machine learning in the prediction of depression treatment outcomes: a systematic review and meta-analysis. psychol Medicine. (2021) 51:2742–51. doi: 10.1017/S0033291721003871 35575607

[58] Kirschbaum C Pirke K-M Hellhammer DH. The ‘Trier Social Stress Test’–a tool for investigating psychobiological stress responses in a laboratory setting. Neuropsychobiology. (1993) 28:76–81. doi: 10.1159/000119004 8255414

[59] Birkett MA. The Trier Social Stress Test protocol for inducing psychological stress. JoVE (Journal Visualized Experiments). (2011) 56):e3238. doi: 10.3791/3238 PMC3227197 22042290

[60] Linares NN Charron V Ouimet A Labelle P Plamondon H. A systematic review of the Trier Social Stress Test methodology: Issues in promoting study comparison and replicable research. Neurobiol stress. (2020) 13:100235. doi: 10.1016/j.ynstr.2020.100235 33344691 PMC7739033

[61] Allen AP Kennedy PJ Cryan JF Dinan TG Clarke G. Biological and psychological markers of stress in humans: Focus on the Trier Social Stress Test. Neurosci biobehavioral Rev. (2014) 38:94–124. doi: 10.1016/j.neubiorev.2013.11.005 24239854

[62] Giakoumis D Drosou A Cipresso P Tzovaras D Hassapis G Gaggioli A. Using activity-related behavioural features towards more effective automatic stress detection. PLoS ONE (2012) 7(9). doi: 10.1371/journal.pone.0043571 PMC3446965 23028461

[63] Gunnar MR Reid BM Donzella B Miller ZR Gardow S Tsakonas NC. Validation of an online version of the Trier Social Stress Test in a study of adolescents. Psychoneuroendocrinology. (2021) 125:105111. doi: 10.1016/j.psyneuen.2020.105111 33341502 PMC7904651

[64] Cheetham TJ Turner-Cobb JM. Panel manipulation in social stress testing: the bath experimental stress test for children (BEST-C). Psychoneuroendocrinology. (2016) 63:78–85. doi: 10.1016/j.psyneuen.2015.09.013 26422711

[65] DeJoseph M Finegood E Raver CC Blair CB. Measuring stress reactivity in the home: Preliminary findings from a version of the Trier Social Stress Test (TSST-H) appropriate for field-based research. (2019). doi: 10.31234/osf.io/5qapw

[66] Hawn SE Paul L Thomas S Miller S Amstadter AB. Stress reactivity to an electronic version of the Trier Social Stress Test: a pilot study. Front Psychol. (2015) 6:724. doi: 10.3389/fpsyg.2015.00724 26074862 PMC4447999

[67] Riem MM Kunst LE Bekker MH Fallon M Kupper N. Intranasal oxytocin enhances stress-protective effects of social support in women with negative childhood experiences during a virtual Trier Social Stress Test. Psychoneuroendocrinology. (2020) 111:104482. doi: 10.1016/j.psyneuen.2019.104482 31677411

[68] Starcke K van Holst RJ van den Brink W Veltman DJ Goudriaan AE. Physiological and E ndocrine R eactions to P sychosocial S tress in A lcohol U se D isorders: D uration of A bstinence M atters. Alcoholism: Clin Exp Res. (2013) 37:1343–50. doi: 10.1111/acer.2013.37.issue-8 23488992

[69] Smith KE Leitzke BT Pollak SD. Youths’ processing of emotion information: Responses to chronic and video-based laboratory stress. Psychoneuroendocrinology. (2020) 122:104873. doi: 10.1016/j.psyneuen.2020.104873 33070023 PMC7686118

[70] Tollenaar MS Overgaauw S. Empathy and mentalizing abilities in relation to psychosocial stress in healthy adult men and women. Heliyon. (2020) 6:e04488. doi: 10.1016/j.heliyon.2020.e04488 32904299 PMC7452492

[71] Wolpe J. Psychotherapy by reciprocal inhibition. Conditional reflex: A Pavlovian J Res Ther. (1968) 3:234–40. doi: 10.1007/BF03000093 5712667

[72] Christian C Cash E Cohen DA Trombley CM Levinson CA. Electrodermal activity and heart rate variability during exposure fear scripts predict trait-level and momentary social anxiety and eating-disorder symptoms in an analogue sample. Clin psychol Science. (2023) 11:134–48. doi: 10.1177/21677026221083284

[73] Hope DA Heimberg RG Turk CL. Managing social anxiety: A cognitive-behavioral therapy approach: Therapist guide. USA: Oxford University Press (2010).

[74] Kim D Bae H Park YC. Validity of the subjective units of disturbance scale in EMDR. J EMDR Pract Res. (2008) 2:57–62. doi: 10.1891/1933-3196.2.1.57

[75] Tanner BA. Validity of global physical and emotional SUDS. Appl Psychophysiol biofeedback. (2012) 37:31–4. doi: 10.1007/s10484-011-9174-x 22038278

[76] Finnerty A. WHOQOL translation Methodology (2020). Available online at: https://www.who.int/tools/whoqol/whoqol-100/docs/default-source/publishing-policies/whoqol-100-guidelines/translation-methodology (Accessed May 31, 2025).

[77] Devito AJ Kubis JF. Alternate forms of the state-trait anxiety inventory. Educ psychol Measurement. (1983) 43:729–34. doi: 10.1177/00131644830430030

[78] Van der Ploeg H. Validity of the zelf-beoordelings-vragenlijst (A dutch version of the spielberger state-trait anxiety inventory). In: Nederlands Tijdschrift voor de Psychologie en haar Grensgebieden (1980) 35(4):243–49.

[79] Spielberger CD Gonzalez-Reigosa F Martinez-Urrutia A Natalicio LF Natalicio DS. The state-trait anxiety inventory. Rev Interamericana Psicologia/Interamerican J Psychol. (1971) 5:3–4.

[80] Spielberger CD. Manual for the State-Trait Anxiety Inventory. Palo Alto, CA: Consulting Psychologists Press (1983).

[81] Ekman P Friesen WV. Measuring facial movement. Environ Psychol nonverbal behavior. (1976) 1:56–75. doi: 10.1007/Bf01115465

[82] Kanaujia A Huang Y Metaxas D. (2006). Tracking facial features using mixture of point distribution models, in: Computer Vision, Graphics and Image Processing: 5th Indian Conference, ICVGIP 2006, Madurai, India, December 13-16, 2006. New York City, New York, United States: Springer.

[83] Baltrusaitis T Zadeh A Lim YC Morency L-P. (2018). Openface 2.0: Facial behavior analysis toolkit, in: 2018 13th IEEE international conference on automatic face & gesture recognition (FG 2018), Piscataway, New Jersey, United States: IEEE.

[84] Amberscript. Speech-to-text API. Amsterdam, the Netherlands: Amberscript (2024).

[85] Kivits N. Van spraak naar tekst: vijf transcriptie-apps getest (2017). Available online at: https://www.villamedia.nl/artikel/van-spraak-naar-tekst-vijf-transcriptie-apps-getest (Accessed May 31, 2025).

[86] Vollebregt M. Automatisch transcriberen? In: Bepaald nog niet perfect (2020). Available at: https://www.journalismlab.nl/automatisch-transcriberen-bepaald-nog-niet-perfect/.

[87] De Geus EJ Willemsen GH Klaver CH van Doornen LJ. Ambulatory measurement of respiratory sinus arrhythmia and respiration rate. Biol Psychol. (1995) 41:205–27. doi: 10.1016/0301-0511(95)05137-6 8608201

[88] Willemsen GH DeGeus EJ Klaver CH VanDoornen LJ Carrofl D. Ambulatory monitoring of the impedance cardiogram. Psychophysiology. (1996) 33:184–93. doi: 10.1111/j.1469-8986.1996.tb02122.x 8851246

[89] McFee B Raffel C Liang D Ellis DP McVicar M Battenberg E. librosa: Audio and music signal analysis in python. In: Proceedings of the 14th python in science conference (2015) 18–24.

[90] Pedregosa F Varoquaux G Gramfort A Michel V Thirion B Grisel O. Scikit-learn: machine learning in python. J Mach Learn Res. (2011) 12:2825–30.

[91] Cawley GC. Leave-one-out cross-validation based model selection criteria for weighted LS-SVMs. In: The 2006 IEEE international joint conference on neural network proceedings. Piscataway, New Jersey, United States: IEEE (2006).

[92] Maftoun M Joloudari JH Zare O Khademi M Atashi A Nematollahi MA. Improving prediction of mortality in ICU via fusion of selectKBest with SMOTE method and extra tree classifier. In: International work-conference on the interplay between natural and artificial computation;. New York City, New York, United States: Springer (2024).

[93] Pratiwi AI Adiwijaya. The feature selection and classification are based on information gained for document sentiment analysis. Appl Comput Intell Soft Computing. (2018) 2018:1407817. doi: 10.1155/2018/1407817

[94] Neal RM. Bayesian learning for neural networks: Springer Science & Business Media;. New York City, NY, United States: Springer (2012).

[95] Bishop C. Pattern recognition and machine learning. Springer google schola. (2006) 2:531–7.

[96] Ghassemi S Zhang T Van Breda W Koutsoumpis A Oostrom JK Holtrop D. Unsupervised multimodal learning for dependency-free personality recognition. In: IEEE transactions on affective computing (2023) 1053–66.

[97] Cohen J. Statistical power analysis for the behavioral sciences. Cambridge, Massachusetts, United States: Academic press; (2013).

[98] Siirtola P Röning J. Comparison of regression and classification models for user-independent and personal stress detection. Sensors. (2020) 20:4402. doi: 10.3390/s20164402 32784547 PMC7472084

[99] Allwright S. What is a good F1 score and how do I interpret it. Luettavissa. (2022) 12:2023. https://stephenallwright.com/good-f1-score/Luettu (Accessed June 1, 2023).

[100] Liu X-Y Li Q-Q Zhou Z-H. Learning imbalanced multi-class data with optimal dichotomy weights. In: 2013 IEEE 13th international conference on data mining. Piscataway, New Jersey, United States: IEEE (2013).

[101] Allwright S. What is a good accuracy score in machine learning? (2022). Available online at: https://stephenallwrightcom/good-accuracy-score/ (Accessed June 1, 2023).

[102] Grimm B Talbot B Larsen L. PHQ-V/GAD-V: assessments to identify signals of depression and anxiety from patient video responses. Appl Sci. (2022) 12:9150. doi: 10.3390/app12189150

[103] Jing C Liu X Zhao N Zhu T. (2019). Different performances of speech and natural gait in identifying anxiety and depression, in: Human Centered Computing: 5th International Conference, HCC 2019, Čačak, Serbia, August 5–7, 2019. New York City, New York, United States: Springer.

[104] Zhang J Yin H Zhang J Yang G Qin J He L. Real-time mental stress detection using multimodality expressions with a deep learning framework. Front Neurosci. (2022) 16:947168. doi: 10.3389/fnins.2022.947168 35992909 PMC9389269

[105] Giannakakis G Pediaditis M Manousos D Kazantzaki E Chiarugi F Simos PG. Stress and anxiety detection using facial cues from videos. Biomed Signal Process Control. (2017) 31:89–101. doi: 10.1016/j.bspc.2016.06.020

[106] Vaessen T Rintala A Otsabryk N Viechtbauer W Wampers M Claes S. The association between self-reported stress and cardiovascular measures in daily life: A systematic review. PloS One. (2021) 16:e0259557. doi: 10.1371/journal.pone.0259557 34797835 PMC8604333

[107] Helminen EC Morton ML Wang Q Felver JC. Stress reactivity to the trier social stress test in traditional and virtual environments: a meta-analytic comparison. Psychosomatic medicine. (2021) 83:200–11. doi: 10.1097/PSY.0000000000000918 33534392

[108] Therrien R Doyle S. Role of training data variability on classifier performance and generalizability. In: Medical imaging 2018: digital pathology. Bellingham, Washington, United States: SPIE (2018).

[109] Meehan AJ Lewis SJ Fazel S Fusar-Poli P Steyerberg EW Stahl D. Clinical prediction models in psychiatry: a systematic review of two decades of progress and challenges. Mol Psychiatry. (2022) 27:2700–8. doi: 10.1038/s41380-022-01528-4 PMC9156409 35365801

[110] Chan EY Saqib NU. Privacy concerns can explain unwillingness to download and use contact tracing apps when COVID-19 concerns are high. Comput Hum Behavior. (2021) 119:106718. doi: 10.1016/j.chb.2021.106718 PMC7840411 33526957

[111] Raji ID Gebru T Mitchell M Buolamwini J Lee J Denton E. Saving face: Investigating the ethical concerns of facial recognition auditing. In: Proceedings of the AAAI/ACM conference on AI, ethics, and society Washington D. C., United States: AAAI (2020).

[112] Nahum-Shani I Smith SN Spring BJ Collins LM Witkiewitz K Tewari A. Just-in-time adaptive interventions (JITAIs) in mobile health: key components and design principles for ongoing health behavior support. Ann Behav Med. (2018), 1–17. doi: 10.1007/s12160-016-9830-8 27663578 PMC5364076

[113] van Genugten C Smit A Thong M Riper H Sprangers M van Ballegooijen W. Just-in-time-adaptive interventions in mental health: A scoping review. Charlottesville, Virginia: Center for Open Science (2023).

