# Supplementary material for: Machine-learning detection of stress severity expressed on a continuous scale using acoustic, verbal, visual, and physiological data: lessons learned
Source: Front Psychiatry. 2025 Jun 13;16:1548287. doi: 10.3389/fpsyt.2025.1548287 (PMC12203116; doi:10.3389/fpsyt.2025.1548287)
Supplement: Supplementary file 1 [file DataSheet1.pdf]

Supplementary Material

Appendix A

Glossary of Terms Used in the Text

Categorical detection of stress. Detection of stress in terms of its presence or absence, or in terms of categories of severity (for example, “no stress”, “mild stress”, “moderate stress” and “severe stress”) (Ciharova et al., 2024).

Continuous detection of stress. In the context of the current study, continuous detection of stress refers to detection in terms of severity, for example, as a score on a continuous scale (e.g., on a scale from 0 to 100) (Ciharova et al., 2024). However, in other context, it may also mean detection over time, i.e., measured at multiple time points (Fazeli et al., 2023).

Detection of stress via machine learning. Recognizing that stress is present or absent in available data, alternatively its severity, by using machine-learning algorithms. Interchangeable with “prediction of stress via machine learning”. However, in some context, prediction aims to estimate the probability of outcome happening in the future. Hence, we use the word “detection”, which is more commonly used for outcomes measured in the present (Salehi et al., 2020).

Dichotomous detection of stress. Detection of stress in terms of its presence or absence (Ciharova et al., 2024).

Ecological momentary assessment. Recurrent assessment of self-reported (mental health) outcome close to real time in the individual’s ecological habitat (Shiffman, 2009).

Monitoring. Real-time or close to real-time information gathering about a (mental) state, its improvement and deterioration (Wang et al., 2024).

Multimodal data. Data obtained from multiple data sources and collected in different manners, ultimately providing a richer dataset and more contextual information (Schultebraucks et al., 2022).

Passive sensing. Unobtrusive collection of data from a participant, which may be partially or fully depending on digital devices, such as smartphones or sensory devices (Cornet & Holden, 2018). Capturing individuals' behavior and biology through digital devices is also often referred to as "digital phenotyping" (Oudin et al., 2023; Torous et al., 2016).

Appendix B

Differences between the Protocol and the Final Manuscript

Due to time constraints and restrictions due to the COVID-19 pandemic, we did not recruit the full planned sample size, i.e., 100 participants. Small sample caused that our model could not detect patterns in the features. Therefore, we decide to conduct a post-hoc analysis, in which we categorized participants into stressed and non-stressed. Finally, we originally aimed to predict the scores on STAI-A State. However, as this measure turned out to be too time-demanding to be administered after every period of the experiment, we decided to only use SUDS at all tested time points.

Appendix C

Point-by-point description of experimental periods

Period	Length	Goal	Activity	Experimenter and Interviewers present?
**Resting (P1)**	5 minutes	Stabilization of physiological and emotional responses	Watching a relaxation video portraying islands (part 1)	No
**Preparation for the speech task (P2)**	5 minutes	Testing anticipatory stress reaction	Written preparation for a presentation about the participant's strengths and weaknesses as part of an interview of an ideal job	No (present in the call, but with cameras switched off)
**Speech task (P3)**	5 minutes	Testing stress reaction to social stress	Delivery of the presentation. If not presenting for 20 s, prompted to continue by the experimenter.	Yes
**Arithmetic stress task (P4)**	5 minutes	Testing stress reaction to cognitive and social evaluative stress	Subtracting 13 from 1022 (out loud). If a mistake was made, prompted by the experimenter to start from the beginning. If not speaking for 20 s, prompted to continue by the experimenter.	Yes
**Recovery (P5)**	10 minutes	Stabilization of physiological and emotional responses	Watching a relaxation video portraying islands (part 2)	No

Pictures of the used solutions

Set-up of the experiment

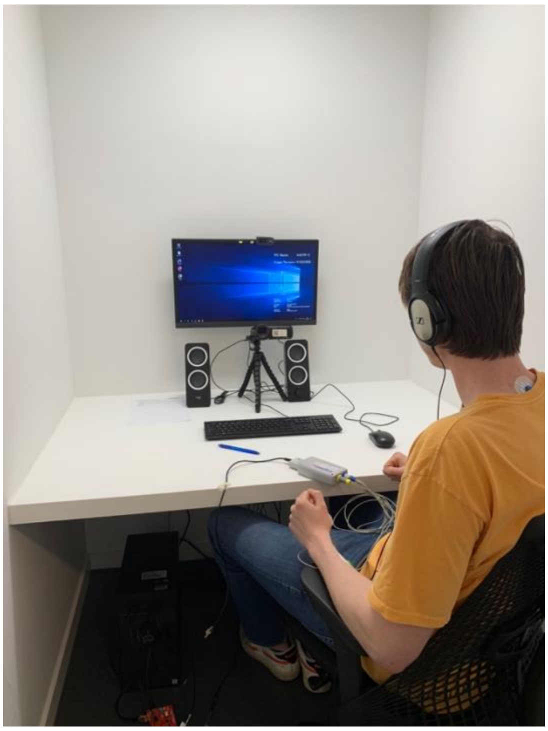

Pre-recorded interviewers

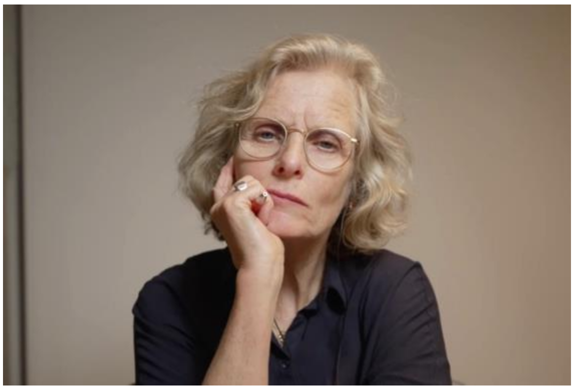

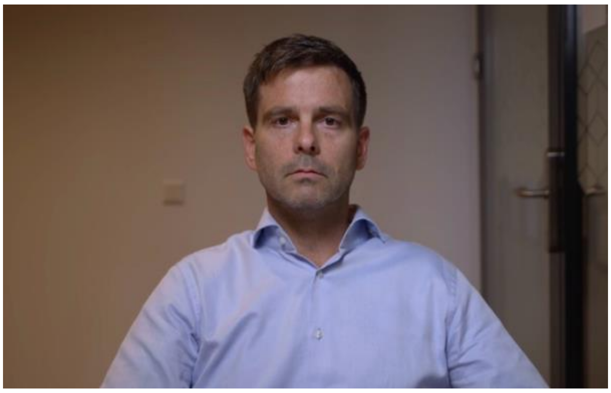

Note. Pictures are used with the permission of the depicted people, namely a volunteer of the piloting of the experiment (i.e., not a participant in the current study), and professional actors playing pre-recorded interviewers in the videocall with participants.

Relaxation video

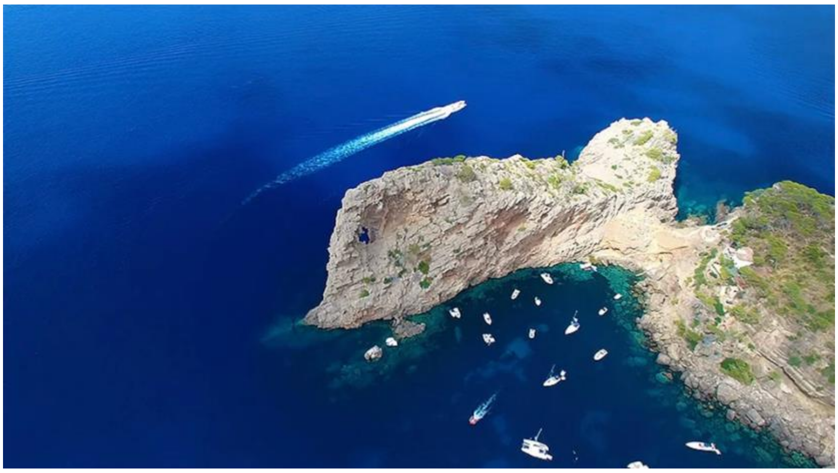

An Example of a Questionnaire Administered during the Experiment (Subjective Units of Distress Scale)

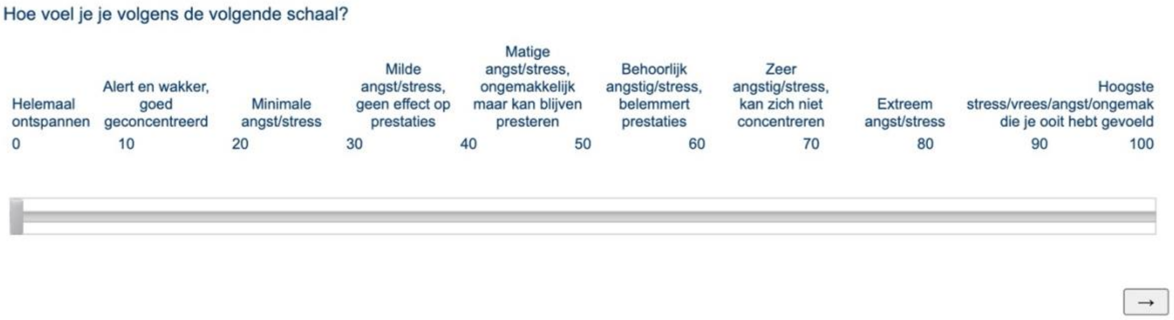

Appendix D

Acoustic features used in stress prediction

Pitch intensity and frequency

Pitch refers to a subjective sensation, which is associated with the frequency of periodic sound waves. Frequency is the number of wavelengths fitting into one unit of time (Thompson et al., 2012).

Formant frequency and bandwidth

A formant is a resonance in the vocal tract, a peak of frequency with high energy (Abhang et al., 2016). Its frequency and bandwidth are related to the articulated letters and their quality (Fleischer et al., 2015).

Harmonic to noise ratio

Harmonic to noise ratio refers to the ratio between two parts of the acoustic wave of a sustained vowel, namely the period part, i.e., vocal fold regular sign, and the additional noise coming from the vocal tract (Ferrand, 2002).

Zero crossing rate

Zero crossing rate characterizes how noisy the signal is (Giannakopoulos & Pikrakis, 2014).

Mel Frequency Cepstral Coefficients (MFCCs)

MFCCs create together the mel-frequency cepstrum, a short-term power spectrum of a sound. They are estimated as the amplitude of a spectrum resulting from a Fourier transform of a signal, overlapping of windows, and calculating logs and discrete cosine transforms from the result (Abdulsatar et al., 2019).

Linear Cepstral Coefficients (LFCCs)

LFCCs are derived by creating a model from the primary vibrations of the vocal folds, and filtering it with one or more resonant frequencies, due to the shape and movement of the vocal tract. The resulting technique aims to separate the source from the filter by estimating the frequency response of the vocal tract (Sueur & Sueur, 2018).

Appendix E

Physiological variables used in stress prediction

ECG derived variables

Inter beat interval (IBI) – average, maximum, and minimum. The time difference between two successive R peaks.

Root-mean-square of successive differences (RMSSD). A time-based measure of heart rate variability, expressed as the root of squared differences between successive R-R intervals in milliseconds.

Standard deviation of the normal-to-normal interval (SDNN). A time-based measure of heart rate variability, expressed as the standard deviation of a set of inter beat intervas in milliseconds. N-N interval is a synonym of the R-R interval.

Low frequency heart rate variability (LF). A frequency-based measure of heart rate variability, expressing how big of a portion of the signal lies within low frequency bands (0.04-0.15 Hz).

High frequency heart rate variability (HF). A frequency-based measure of heart rate variability, expressing how big of a portion of the signal lies within low frequency bands (0.15-0.40 Hz).

ICG derived variables

Stroke volume (SV). The average amount of blood ejected during the cardiac cycle in cm^3. It is calculated from the ICG based on the maximal amplitude of the dZ/dt, the ejection time, baseline thorax impedance, and the total volume, using the Kubicek's equation with the Nederend correction (Kubicek, 1966; Nederend et al., 2018).

Minute volume (MV). The amount of gas inhaled or exhaled from an individual's lungs in one minute, calculated using Kubicek's equation with the correction by Nederend (Kubicek, 1966; Nederend et al., 2018).

Respiratory sinus arrythmia (RSA) – average, maximum, minimum, and standard deviation. RSA is a variable combining information from heart rate variability and respiration, namely R-peak time series with the impedance derived respiration cycle, using a peak valley method. This method detects the shortest IBI during every inspiration and the longest IBI during every expiration, subsequently subtracting the former from the latter. Zero or negative values are then coded as zero (De Geus et al., 1995; Goedhart et al., 2007). A measure of parasympathetic nervous activity (Berntson et al., 1993; Katona & Jih, 1975; Migliaro, 2020).

often related to stress in literature (Beauchaine, 2015; Beauchaine et al., 2019; Campbell et al., 2019; Tonhajzerova et al., 2016).

Pre-ejection period (PEP). The PEP is the time difference between the onset of ventricular depolarization in the ECG signal and the time of the opening of the aortic valves in the ICG (Nederend et al., 2018; Willemsen et al., 1996). For each period of interest, we derived a single averaged ICG complex by ensemble averaging the ICG signal over all R-peaks in the conditions (Riese et al., 2003), a previously validated method (Nederend et al., 2018; Willemsen et al., 1996). Shorter PEP indicates more stress, because it is related to increases of sympathetic nervous system (Brindle et al., 2014; Rahman et al., 2018; van der Mee et al., 2020; van der Mee et al., 2021).

Left ventricular ejection time (LVET). LVET is the time it takes to eject the blood from the left ventricle into the aorta. It is expected to decrease with increased sympathetic nervous system activity.

T-wave amplitude (TWA). TWA means the difference between the peak of the T wave (T) and the value at the end of repolarization (T-offset), which are both reflected in the ICG signal. With increasing sympathetic nervous activity, for example due to stress, the TWA decreases.

Respiration rate (RR) – average, minimum, maximum, and standard deviation. RR refers to breaths per minute.

Tidal volume (TV) – average, minimum, maximum, and standard deviation. TV is the amount of air that moves in and out of the lungs with each respiratory cycle.

EDA

A measure of electrodermal activity of the skin regulated by the sweat glands of the body.

Nonspecific skin conductance responses per minute (nsSCRs). NsSCRs frequency is the number of peaks per minute, it is associated with negative emotions. arousal and stress (Kelsey, 1991; Miller & Shmavonian, 1965; Nikula, 1991; van der Mee et al., 2021), and has been shown to be a better indicator of changes in sympathetic nervous system than skin conductance level (van der Mee et al., 2021). It refers to changes in skin conductance happening spontaneously, without a clear relation to discrete external stimuli.

Skin conductance (SCL) – average, minimum, and maximum. The level of skin conductance obtained over longer periods, a measure of the individual’s arousal state, which is, however, sensitive to environmental changes.

Accelerometry derived variables

Motility - average X, Y, and Z. Average X, Y, and Z motility was calculated as the X-, Y-, and Z-axis accelerations.

Motility – total. Total motility was computed as the root of the mean of the squared accelerations on the X, Y and Z axis.

Appendix F

Table S1

Correlations between visual features and stress at T1 through T5

	T1 - SUDS		T2 - SUDS		T3 - SUDS		T4 - SUDS		T5 - SUDS	
	r	p	r	p	r	p	r	p	r	p
**Action units**										
visual_AU01_r_curvature	-.169	.371	-.072	.702	-.049	.795	.193	.297	-.191	.331
visual_AU01_r_kurtosis	.231	.219	-.171	.358	.082	.661	.073	.694	.129	.514
visual_AU01_r_max	.243	.196	-.252	.172	.095	.611	.164	.377	.045	.822
visual_AU01_r_mean	.214	.256	-.044	.816	-.062	.741	.098	.601	.150	.446
visual_AU01_r_median	-.146	.442	.062	.742	-.124	.506	-.145	.435	-.006	.976
visual_AU01_r_min	.000	.710	.000	1.000	.000	1.000	.000	1.000	.000	.905
visual_AU01_r_offset	.070	.712	-.103	.581	-.166	.372	.253	.170	.140	.478
visual_AU01_r_skewness	.200	.290	-.120	.521	.027	.885	.020	.914	.071	.719
visual_AU01_r_slop	.016	.935	.094	.615	.204	.271	-.300	.101	-.117	.553
visual_AU01_r_std	.205	.276	-.086	.647	-.059	.755	.096	.607	.150	.447
visual_AU02_r_curvature	-.194	.303	-.261	.157	-.091	.625	.258	.161	-.248	.203
visual_AU02_r_kurtosis	-.002	.994	-.147	.431	-.228	.218	.018	.922	.272	.162
visual_AU02_r_max	.076	.689	-.098	.599	-.045	.810	.258	.162	.074	.710
visual_AU02_r_mean	.118	.533	-.044	.816	-.008	.964	.197	.289	-.122	.536
visual_AU02_r_median	.000	.442	.000	1.000	.000	1.000	.000	1.000	.000	.976
visual_AU02_r_min	.000	.710	.000	1.000	.000	1.000	.000	1.000	.000	.905
visual_AU02_r_offset	.066	.729	-.238	.198	-.164	.378	.126	.500	-.135	.495
visual_AU02_r_skewness	-.047	.804	-.121	.516	-.159	.391	-.023	.904	.236	.227
visual_AU02_r_slop	-.042	.824	.269	.144	.199	.283	-.092	.624	.122	.537
visual_AU02_r_std	.067	.724	-.070	.710	-.031	.868	.211	.255	-.111	.574
visual_AU04_r_curvature	.000	.999	.119	.523	-.079	.674	-.088	.637	-.095	.632
visual_AU04_r_kurtosis	.256	.173	-.268	.145	-.169	.363	-.058	.755	-.070	.722
visual_AU04_r_max	**.472**	**.009**	.021	.911	-.041	.828	-.133	.475	.106	.592
visual_AU04_r_mean	.358	.052	.056	.763	.136	.467	.115	.536	.336	.081
visual_AU04_r_median	.360	.051	.074	.692	.150	.420	.126	.500	.321	.095
visual_AU04_r_min	.277	.139	-.005	.979	.017	.926	-.311	.089	-.132	.504
visual_AU04_r_offset	.358	.052	.049	.795	.104	.578	.138	.460	.268	.168
visual_AU04_r_skewness	.153	.420	-.281	.125	-.325	.075	-.145	.437	-.160	.416
visual_AU04_r_slop	.056	.770	.015	.935	.109	.561	-.066	.724	.157	.425
visual_AU04_r_std	.143	.452	.230	.214	.123	.510	.004	.981	-.033	.867
visual_AU05_r_curvature	-.341	.065	-.129	.488	.073	.696	.346	.057	.173	.377
visual_AU05_r_kurtosis	.168	.375	-.028	.880	**-.395**	**.028**	-.001	.997	-.249	.201
visual_AU05_r_max	-.029	.881	.069	.714	**-.387**	**.031**	-.087	.644	-.279	.151
visual_AU05_r_mean	-.219	.246	.127	.496	-.265	.149	.003	.989	-.096	.629
visual_AU05_r_median	.000	.051	.000	1.000	.000	1.000	.000	1.000	.000	.095
visual_AU05_r_min	.000	.139	.000	1.000	.000	1.000	.000	1.000	.000	.504
visual_AU05_r_offset	-.225	.232	.078	.675	-.169	.364	.049	.795	-.286	.140
visual_AU05_r_skewness	.093	.626	-.016	.930	**-.378**	**.036**	-.051	.786	-.263	.177
visual_AU05_r_slop	.189	.317	-.020	.916	.092	.624	-.048	.796	.283	.144
visual_AU05_r_std	-.248	.187	.135	.469	-.304	.096	-.008	.966	-.163	.408
visual_AU06_r_curvature	.032	.867	**.380**	**.035**	.266	.149	.029	.875	.215	.272
visual_AU06_r_kurtosis	-.078	.681	.022	.905	-.035	.852	-.252	.171	-.090	.647
visual_AU06_r_max	.113	.552	.167	.369	.123	.509	.012	.947	-.009	.965
visual_AU06_r_mean	.201	.287	.251	.173	.217	.240	-.051	.786	.299	.122
visual_AU06_r_median	.176	.353	.165	.376	.217	.240	-.034	.855	.295	.128
visual_AU06_r_min	.000	.139	.000	1.000	.000	1.000	.000	1.000	.000	.504
visual_AU06_r_offset	.159	.400	**.381**	**.034**	.250	.174	-.116	.535	.275	.157
visual_AU06_r_skewness	-.100	.598	.009	.962	-.007	.972	-.166	.373	-.112	.572
visual_AU06_r_slop	-.069	.717	**-.467**	**.008**	-.228	.218	.170	.360	-.209	.287
visual_AU06_r_std	.265	.158	.226	.221	.069	.712	-.075	.690	.207	.290
visual_AU07_r_curvature	-.018	.926	.071	.706	.293	.109	.136	.465	-.355	.064
visual_AU07_r_kurtosis	-.268	.153	-.108	.563	.058	.757	.229	.215	-.147	.457
visual_AU07_r_max	**.423**	**.020**	-.002	.993	-.075	.688	-.186	.316	-.223	.254
visual_AU07_r_mean	.223	.237	-.164	.378	-.038	.839	.088	.637	.045	.820
visual_AU07_r_median	.212	.262	-.181	.329	-.030	.871	.134	.473	.023	.908
visual_AU07_r_min	.108	.568	.000	1.000	.000	1.000	-.258	.161	.302	.118
visual_AU07_r_offset	.220	.244	-.122	.512	-.066	.725	-.035	.850	.064	.746
visual_AU07_r_skewness	-.261	.163	-.012	.949	.027	.885	.024	.899	-.209	.285
visual_AU07_r_slop	-.009	.962	-.092	.621	.099	.594	.286	.119	-.067	.735

	T1 - SUDS		T2 - SUDS		T3 - SUDS		T4 - SUDS		T5 - SUDS	
	**r**	**p**	**r**	**p**	**r**	**p**	**r**	**p**	**r**	**p**
visual_AU07_r_std	.296	.113	-.055	.770	-.053	.779	-.105	.573	.023	.908
visual_AU09_r_curvature	.051	.789	**.423**	**.018**	.001	.995	**-.357**	**.049**	-.213	.275
visual_AU09_r_kurtosis	.111	.559	.079	.671	.066	.725	-.005	.977	-.066	.740
visual_AU09_r_max	.055	.771	.161	.387	.066	.723	.033	.860	.110	.577
visual_AU09_r_mean	.117	.537	.109	.558	.011	.954	-.098	.600	.067	.734
visual_AU09_r_median	.000	.262	.000	1.000	.000	1.000	.000	1.000	.000	.908
visual_AU09_r_min	.000	.568	.000	1.000	.000	1.000	.000	1.000	.000	.118
visual_AU09_r_offset	.116	.543	.159	.392	.130	.487	-.304	.096	-.111	.574
visual_AU09_r_skewness	.045	.815	.043	.820	-.084	.652	.004	.983	-.026	.894
visual_AU09_r_slop	-.098	.607	-.137	.464	-.106	.569	.216	.242	.136	.491
visual_AU09_r_std	.093	.626	.103	.581	-.058	.759	-.108	.564	.126	.522
visual_AU10_r_curvature	-.001	.994	.268	.145	.173	.351	-.123	.509	.112	.571
visual_AU10_r_kurtosis	-.112	.557	.034	.856	-.001	.998	-.251	.174	.178	.364
visual_AU10_r_max	.058	.759	-.050	.787	.041	.827	.215	.246	.026	.896
visual_AU10_r_mean	.276	.140	.167	.369	.125	.504	-.009	.963	.193	.324
visual_AU10_r_median	.288	.123	.153	.412	.116	.533	-.020	.914	.225	.250
visual_AU10_r_min	.000	.568	.000	1.000	.000	1.000	.000	1.000	.000	.118
visual_AU10_r_offset	.185	.328	.185	.319	.112	.548	-.069	.714	.219	.262
visual_AU10_r_skewness	-.098	.605	.007	.970	-.060	.749	-.195	.293	.187	.341
visual_AU10_r_slop	.042	.824	-.179	.335	.006	.973	.177	.342	-.204	.297
visual_AU10_r_std	.223	.236	.095	.610	.009	.962	.068	.715	.137	.486
visual_AU12_r_curvature	.018	.926	.144	.439	.199	.282	-.202	.277	-.077	.695
visual_AU12_r_kurtosis	-.150	.429	.176	.344	-.081	.666	.280	.127	.047	.813
visual_AU12_r_max	-.048	.803	.057	.762	.155	.405	.093	.618	.054	.786
visual_AU12_r_mean	.053	.782	.051	.783	.255	.167	.010	.959	.117	.552
visual_AU12_r_median	.008	.966	.019	.920	.248	.179	.047	.801	.113	.568
visual_AU12_r_min	.000	.568	.000	1.000	.000	1.000	.025	.892	.000	.118
visual_AU12_r_offset	.095	.616	.161	.387	.253	.169	-.031	.868	.118	.548
visual_AU12_r_skewness	-.114	.547	.208	.262	-.192	.302	.301	.100	.016	.934
visual_AU12_r_slop	-.095	.617	-.246	.182	-.085	.650	.082	.661	-.095	.630
visual_AU12_r_std	.219	.246	.043	.818	.182	.327	-.049	.792	.128	.516
visual_AU14_r_curvature	-.141	.456	.083	.657	.019	.918	.135	.470	-.043	.829
visual_AU14_r_kurtosis	-.297	.111	.014	.941	-.129	.488	-.042	.822	.003	.987
visual_AU14_r_max	.247	.189	.149	.424	-.147	.429	-.007	.968	-.084	.669
visual_AU14_r_mean	.301	.106	-.035	.853	.151	.417	.110	.555	.130	.511
visual_AU14_r_median	.000	.966	.000	1.000	.252	.172	.142	.445	.000	.568
visual_AU14_r_min	.000	.568	.000	1.000	.000	1.000	.000	1.000	.000	.118
visual_AU14_r_offset	.275	.141	.013	.946	.273	.137	-.024	.899	.139	.481
visual_AU14_r_skewness	-.336	.070	.103	.580	-.088	.639	-.009	.963	-.077	.697
visual_AU14_r_slop	.048	.799	-.062	.739	-.294	.108	.271	.140	-.032	.870
visual_AU14_r_std	.360	.051	-.072	.699	.044	.814	.014	.941	.075	.705
visual_AU15_r_curvature	.239	.204	.298	.104	-.336	.065	-.161	.387	-.346	.071
visual_AU15_r_kurtosis	-.118	.533	.135	.468	-.233	.208	-.118	.526	.033	.867
visual_AU15_r_max	.083	.664	.265	.150	-.194	.295	-.029	.879	.053	.789
visual_AU15_r_mean	.199	.292	.055	.769	.035	.853	.040	.829	.026	.894
visual_AU15_r_median	.303	.103	-.076	.686	-.355	.050	-.284	.121	-.054	.785
visual_AU15_r_min	.000	.568	.000	1.000	.000	1.000	.000	1.000	.000	.118
visual_AU15_r_offset	.347	.060	-.109	.560	.292	.111	.083	.656	.035	.859
visual_AU15_r_skewness	.001	.997	.149	.424	-.247	.181	-.144	.440	.038	.847
visual_AU15_r_slop	-.319	.086	.197	.289	-.203	.272	-.042	.823	-.040	.839
visual_AU15_r_std	.200	.289	.092	.623	-.066	.724	.005	.977	.039	.842
visual_AU17_r_curvature	-.122	.520	.126	.501	-.232	.208	.088	.640	.156	.427
visual_AU17_r_kurtosis	-.090	.636	.006	.973	.068	.715	.304	.096	-.052	.793
visual_AU17_r_max	.115	.544	.148	.428	-.057	.762	.161	.388	-.032	.873
visual_AU17_r_mean	.224	.234	-.252	.171	-.112	.548	-.060	.750	-.064	.745
visual_AU17_r_median	.212	.261	-.287	.118	-.159	.394	-.177	.342	-.052	.795
visual_AU17_r_min	.000	.568	.000	1.000	.000	1.000	.000	1.000	.000	.118
visual_AU17_r_offset	.031	.873	-.022	.906	-.089	.632	.062	.740	-.040	.841
visual_AU17_r_skewness	-.149	.432	.052	.782	.065	.728	.346	.057	-.053	.789
visual_AU17_r_slop	.204	.280	-.132	.480	.046	.808	-.120	.520	-.006	.977
visual_AU17_r_std	.209	.268	-.235	.203	-.071	.704	.072	.699	-.077	.697
visual_AU20_r_curvature	.023	.906	.315	.084	-.322	.078	-.040	.831	-.084	.670
visual_AU20_r_kurtosis	-.206	.275	.158	.395	-.249	.177	.187	.314	.240	.218
visual_AU20_r_max	-.124	.515	-.024	.899	-.231	.212	.133	.475	-.037	.853
visual_AU20_r_mean	-.089	.640	-.112	.547	-.023	.901	.171	.359	-.090	.649

	T1 - SUDS		T2 - SUDS		T3 - SUDS		T4 - SUDS		T5 - SUDS	
	r	p	r	p	r	p	r	p	r	p
visual_AU20_r_median	.133	.483	.000	1.000	.000	1.000	.000	1.000	.000	.795
visual_AU20_r_min	.000	.568	.000	1.000	.000	1.000	.000	1.000	.000	.118
visual_AU20_r_offset	-.072	.705	-.084	.654	.232	.210	.189	.308	-.154	.433
visual_AU20_r_skewness	-.163	.388	.166	.374	-.332	.068	.249	.176	.215	.272
visual_AU20_r_slop	.050	.793	.059	.751	-.297	.105	-.132	.478	.163	.409
visual_AU20_r_std	-.151	.427	-.086	.644	-.138	.458	.251	.174	-.085	.667
visual_AU23_r_curvature	-.309	.096	-.063	.737	**-.475**	**.007**	.100	.594	.344	.073
visual_AU23_r_kurtosis	-.067	.726	.083	.657	-.125	.501	-.301	.100	.004	.984
visual_AU23_r_max	.201	.287	.088	.637	-.023	.902	.108	.563	.087	.661
visual_AU23_r_mean	.192	.311	-.052	.781	.163	.382	.282	.125	.166	.399
visual_AU23_r_median	.000	.483	.000	1.000	.000	1.000	.000	1.000	.000	.795
visual_AU23_r_min	.000	.568	.000	1.000	.000	1.000	.000	1.000	.000	.118
visual_AU23_r_offset	.270	.148	-.159	.394	.133	.477	.046	.806	.194	.322
visual_AU23_r_skewness	-.041	.828	.063	.735	-.132	.480	-.302	.099	.005	.980
visual_AU23_r_slop	-.159	.401	.139	.455	-.056	.766	.072	.702	-.074	.708
visual_AU23_r_std	.227	.228	-.048	.796	.127	.497	.242	.189	.142	.472
visual_AU25_r_curvature	**-.457**	**.011**	.272	.139	-.026	.889	-.238	.197	-.040	.839
visual_AU25_r_kurtosis	-.136	.473	-.210	.256	.021	.910	.080	.669	.155	.430
visual_AU25_r_max	-.154	.417	-.263	.153	-.078	.678	.003	.986	-.042	.833
visual_AU25_r_mean	-.029	.879	-.117	.532	-.040	.830	.114	.540	-.187	.340
visual_AU25_r_median	.025	.894	-.127	.497	-.042	.823	-.054	.771	-.083	.673
visual_AU25_r_min	.000	.568	.000	1.000	.000	1.000	.000	1.000	.000	.118
visual_AU25_r_offset	.013	.947	-.037	.841	.003	.989	-.114	.541	-.324	.092
visual_AU25_r_skewness	-.037	.845	-.169	.364	-.015	.935	.214	.248	.128	.515
visual_AU25_r_slop	-.031	.871	-.052	.780	-.050	.791	.214	.249	.349	.068
visual_AU25_r_std	-.015	.936	-.155	.407	-.033	.860	.221	.231	-.230	.239
visual_AU26_r_curvature	-.111	.559	.152	.414	.152	.415	.328	.072	-.065	.742
visual_AU26_r_kurtosis	.131	.490	.084	.652	**-.458**	**.010**	-.296	.106	-.170	.387
visual_AU26_r_max	.065	.732	.026	.890	-.310	.090	.146	.434	-.099	.618
visual_AU26_r_mean	.056	.769	.059	.752	-.010	.956	.222	.231	-.047	.813
visual_AU26_r_median	.069	.716	.104	.576	-.027	.885	.129	.489	-.032	.870
visual_AU26_r_min	.000	.568	.000	1.000	.000	1.000	.000	1.000	.000	.118
visual_AU26_r_offset	.298	.109	.162	.385	-.017	.928	.273	.137	-.188	.339
visual_AU26_r_skewness	.139	.463	.064	.733	-.244	.186	-.228	.217	-.170	.388
visual_AU26_r_slop	-.307	.099	-.168	.366	.020	.916	-.278	.130	.194	.323
visual_AU26_r_std	.069	.716	.063	.738	-.009	.963	.228	.218	-.061	.758
visual_AU45_r_curvature	**-.377**	**.040**	-.169	.363	-.184	.321	-.173	.351	**-.510**	**.006**
visual_AU45_r_kurtosis	.018	.926	.068	.718	-.101	.590	-.223	.227	.036	.854
visual_AU45_r_max	-.009	.963	.105	.574	.232	.210	-.168	.366	.031	.876
visual_AU45_r_mean	-.110	.563	-.004	.985	.235	.203	-.109	.561	.024	.903
visual_AU45_r_median	-.087	.646	.066	.724	.056	.765	-.249	.176	.010	.962
visual_AU45_r_min	.000	.568	.000	1.000	.000	1.000	.000	1.000	.000	.118
visual_AU45_r_offset	-.215	.253	.072	.701	-.094	.616	-.070	.707	-.125	.526
visual_AU45_r_skewness	-.035	.853	.058	.759	-.102	.586	-.219	.236	.055	.783
visual_AU45_r_slop	.211	.262	-.087	.640	.223	.228	-.012	.947	.192	.327
visual_AU45_r_std	-.136	.473	-.054	.774	.195	.292	-.142	.445	.026	.895
**Point Distribution Model**										
visual_p_0_curvature	.182	.335	-.163	.381	-.257	.163	-.116	.533	.178	.364
visual_p_0_kurtosis	.003	.989	-.033	.859	-.260	.158	-.234	.205	-.043	.829
visual_p_0_max	.039	.837	-.027	.886	-.003	.986	.226	.221	-.150	.447
visual_p_0_mean	-.147	.438	-.134	.471	.030	.872	.296	.106	-.011	.957
visual_p_0_median	-.158	.405	-.112	.550	.025	.893	.305	.095	.010	.961
visual_p_0_min	-.085	.656	-.113	.544	.137	.462	.186	.316	.234	.231
visual_p_0_offset	-.249	.185	-.137	.463	.095	.613	.312	.088	-.007	.971
visual_p_0_skewness	.128	.501	-.028	.879	-.093	.619	-.283	.123	.094	.634
visual_p_0_slop	.298	.110	.010	.957	-.169	.365	.048	.798	-.007	.972
visual_p_0_std	.177	.349	.009	.960	.094	.615	.121	.517	-.114	.563
visual_p_1_curvature	-.076	.689	-.332	.068	.116	.534	.001	.995	.091	.644
visual_p_1_kurtosis	-.053	.780	.025	.896	.092	.624	.248	.178	-.038	.847
visual_p_1_max	.109	.566	-.071	.705	-.005	.977	-.172	.355	-.012	.952
visual_p_1_mean	-.040	.833	.070	.710	-.189	.309	-.154	.409	.147	.454
visual_p_1_median	-.026	.893	.053	.777	-.168	.368	-.144	.439	.153	.437
visual_p_1_min	-.208	.270	.052	.783	-.040	.832	-.328	.072	.009	.966
visual_p_1_offset	.030	.874	.180	.333	-.127	.494	.018	.922	.068	.732
visual_p_1_skewness	-.104	.586	.063	.736	.002	.992	-.317	.082	-.113	.566

	T1 - SUDS		T2 - SUDS		T3 - SUDS		T4 - SUDS		T5 - SUDS	
	r	p	r	p	r	p	r	p	r	p
visual_p_1_slop	-.132	.486	-.173	.351	-.137	.462	-.140	.454	.173	.380
visual_p_1_std	.293	.116	-.173	.351	.101	.589	.149	.425	-.099	.616
visual_p_10_curvature	.224	.234	.320	.079	.086	.647	-.237	.199	**-.413**	**.029**
visual_p_10_kurtosis	.225	.231	.159	.393	**-.387**	**.031**	-.143	.442	.152	.439
visual_p_10_max	.229	.224	.058	.757	-.233	.208	.096	.608	.038	.848
visual_p_10_mean	.093	.624	.049	.791	-.143	.444	-.038	.841	.113	.566
visual_p_10_median	.061	.748	.060	.750	-.134	.473	-.019	.919	.111	.573
visual_p_10_min	.037	.846	-.073	.698	-.048	.796	-.051	.784	.212	.278
visual_p_10_offset	.201	.287	.071	.704	-.138	.461	.045	.810	.069	.727
visual_p_10_skewness	.225	.232	.238	.197	-.171	.359	.025	.895	.118	.549
visual_p_10_slop	-.311	.094	-.067	.722	.002	.993	-.315	.084	.159	.420
visual_p_10_std	.048	.801	-.008	.964	.080	.668	.108	.564	-.069	.725
visual_p_11_curvature	.055	.772	.075	.689	-.049	.794	-.024	.897	.142	.471
visual_p_11_kurtosis	.013	.947	-.271	.140	**-.421**	**.018**	-.249	.177	-.202	.303
visual_p_11_max	.106	.577	-.017	.926	-.007	.971	-.210	.256	-.006	.976
visual_p_11_mean	.021	.913	-.110	.555	.086	.644	.048	.799	.073	.713
visual_p_11_median	.025	.894	-.103	.582	.099	.597	.029	.876	.076	.701
visual_p_11_min	-.008	.968	-.273	.138	-.072	.701	-.077	.681	.000	.999
visual_p_11_offset	.089	.639	-.022	.907	.150	.420	-.076	.686	-.031	.877
visual_p_11_skewness	-.003	.987	.155	.405	-.096	.606	-.219	.236	-.047	.814
visual_p_11_slop	-.174	.357	-.182	.328	-.202	.276	**.366**	**.043**	.188	.339
visual_p_11_std	.054	.777	.164	.377	**.374**	**.038**	-.005	.979	.066	.740
visual_p_12_curvature	.132	.486	-.082	.659	.138	.459	-.205	.269	-.124	.530
visual_p_12_kurtosis	.081	.670	.059	.754	-.300	.101	-.268	.146	**-.394**	**.038**
visual_p_12_max	.123	.518	.291	.113	-.109	.561	-.002	.993	-.085	.667
visual_p_12_mean	.005	.981	.212	.253	-.084	.654	.068	.716	-.162	.410
visual_p_12_median	-.008	.967	.185	.318	-.089	.634	.111	.553	-.149	.450
visual_p_12_min	-.142	.455	-.043	.817	.025	.893	.005	.978	-.160	.417
visual_p_12_offset	.073	.700	.305	.095	-.092	.622	.123	.509	-.209	.285
visual_p_12_skewness	.129	.496	-.006	.974	.182	.326	-.269	.144	-.140	.478
visual_p_12_slop	-.216	.251	-.321	.078	.038	.839	-.181	.330	.162	.410
visual_p_12_std	.048	.802	.158	.395	.124	.508	.150	.421	-.008	.967
visual_p_13_curvature	-.004	.984	.040	.829	-.228	.218	.017	.926	.329	.088
visual_p_13_kurtosis	.018	.926	-.086	.646	-.115	.537	.206	.267	-.025	.899
visual_p_13_max	.170	.370	-.002	.991	.078	.677	-.054	.772	-.144	.464
visual_p_13_mean	-.059	.758	.092	.622	.044	.812	-.190	.307	.119	.546
visual_p_13_median	-.072	.704	.087	.642	.051	.784	-.170	.361	.113	.568
visual_p_13_min	-.042	.824	-.067	.719	-.087	.640	**-.402**	**.025**	-.054	.784
visual_p_13_offset	.049	.797	.076	.683	.106	.569	-.184	.323	.114	.562
visual_p_13_skewness	.038	.843	-.041	.826	-.023	.904	-.212	.252	-.050	.801
visual_p_13_slop	-.288	.123	.008	.968	-.175	.347	.068	.717	-.001	.997
visual_p_13_std	.221	.241	-.009	.960	.179	.336	.197	.288	.001	.995
visual_p_14_curvature	-.045	.814	-.137	.463	-.059	.754	.184	.321	.039	.843
visual_p_14_kurtosis	.330	.075	-.164	.378	-.250	.174	-.106	.571	.076	.699
visual_p_14_max	.100	.600	-.172	.354	.002	.993	-.056	.765	.098	.620
visual_p_14_mean	-.129	.497	-.353	.051	.040	.832	-.333	.067	.008	.967
visual_p_14_median	-.146	.443	**-.372**	**.039**	.053	.776	-.348	.055	.008	.966
visual_p_14_min	-.134	.480	-.218	.239	-.232	.209	-.280	.126	.194	.322
visual_p_14_offset	-.002	.991	-.315	.084	.019	.918	-.351	.053	.083	.676
visual_p_14_skewness	.221	.240	.168	.365	**-.386**	**.032**	.143	.443	.112	.570
visual_p_14_slop	-.294	.115	-.066	.724	.099	.597	.148	.426	-.223	.254
visual_p_14_std	.007	.971	-.010	.955	.187	.315	.156	.402	-.108	.583
visual_p_15_curvature	.074	.698	.175	.346	-.171	.357	-.001	.998	-.204	.299
visual_p_15_kurtosis	.074	.697	-.182	.327	-.154	.409	.330	.070	-.201	.304
visual_p_15_max	.227	.228	-.022	.906	.022	.905	**.384**	**.033**	.080	.686
visual_p_15_mean	.062	.744	-.093	.618	-.059	.751	.051	.785	.031	.877
visual_p_15_median	.050	.793	-.111	.553	-.051	.785	.022	.905	.030	.879
visual_p_15_min	.076	.688	-.104	.578	-.114	.542	-.139	.454	.036	.854
visual_p_15_offset	.058	.759	-.051	.785	-.015	.936	.111	.552	.013	.948
visual_p_15_skewness	-.006	.976	-.060	.748	.000	.999	.065	.727	.121	.538
visual_p_15_slop	.011	.952	-.090	.630	-.154	.408	-.188	.310	.045	.822
visual_p_15_std	-.023	.905	.056	.765	.218	.239	.282	.124	.048	.807
visual_p_16_curvature	**.427**	**.019**	.336	.064	.261	.156	.098	.598	.194	.322
visual_p_16_kurtosis	.012	.949	-.230	.214	-.119	.523	-.286	.119	-.109	.581
visual_p_16_max	.079	.678	.111	.554	.022	.908	.202	.277	-.042	.831

	T1 - SUDS		T2 - SUDS		T3 - SUDS		T4 - SUDS		T5 - SUDS	
	r	p	r	p	r	p	r	p	r	p
visual_p_16_mean	-.036	.850	-.070	.710	.020	.914	.052	.783	.021	.914
visual_p_16_median	-.048	.803	.012	.950	.023	.904	.036	.849	.010	.959
visual_p_16_min	-.090	.636	-.230	.213	-.100	.591	-.132	.478	.036	.855
visual_p_16_offset	.001	.996	-.012	.948	.044	.812	.013	.946	.038	.846
visual_p_16_skewness	.096	.614	.031	.870	.078	.676	.085	.648	-.097	.624
visual_p_16_slop	-.128	.501	-.124	.506	-.063	.735	.120	.521	-.067	.736
visual_p_16_std	.071	.711	.317	.082	.148	.427	.259	.160	.028	.889
visual_p_17_curvature	-.008	.965	.221	.232	.005	.978	-.140	.453	-.082	.678
visual_p_17_kurtosis	.039	.837	-.303	.098	-.035	.854	.076	.684	.060	.760
visual_p_17_max	.060	.755	.187	.314	.102	.585	.049	.793	-.156	.427
visual_p_17_mean	.122	.519	-.165	.376	.119	.523	-.064	.732	.005	.978
visual_p_17_median	.112	.555	-.137	.462	.120	.520	-.070	.708	-.053	.789
visual_p_17_min	.051	.789	-.175	.346	.011	.953	-.214	.248	.059	.765
visual_p_17_offset	.088	.643	-.210	.258	.149	.425	-.053	.777	.107	.589
visual_p_17_skewness	.035	.856	.012	.950	-.081	.665	-.035	.853	-.331	.085
visual_p_17_slop	.018	.925	.092	.621	-.117	.531	-.004	.984	-.171	.385
visual_p_17_std	.074	.697	.149	.424	.189	.309	.012	.948	-.169	.389
visual_p_18_curvature	.272	.146	.153	.411	-.032	.862	.226	.222	.208	.289
visual_p_18_kurtosis	.027	.889	-.093	.619	-.236	.201	-.205	.268	-.096	.626
visual_p_18_max	.265	.156	.334	.066	-.026	.889	.023	.902	.020	.921
visual_p_18_mean	.082	.668	.317	.083	.255	.166	-.036	.849	.233	.232
visual_p_18_median	.078	.680	.344	.058	.300	.102	-.044	.814	.214	.275
visual_p_18_min	.088	.643	-.087	.643	-.001	.995	-.119	.524	.201	.304
visual_p_18_offset	.108	.570	.229	.216	.218	.239	-.056	.766	.192	.327
visual_p_18_skewness	.056	.768	-.087	.640	-.132	.479	.034	.855	.057	.772
visual_p_18_slop	-.099	.602	.037	.845	.035	.850	.045	.810	.105	.594
visual_p_18_std	.021	.913	.332	.068	.162	.384	.111	.551	.098	.619
visual_p_19_curvature	.066	.729	.189	.307	.332	.068	-.005	.978	-.252	.195
visual_p_19_kurtosis	.018	.923	-.134	.474	-.057	.760	-.221	.232	-.146	.457
visual_p_19_max	.315	.090	.126	.501	-.151	.418	-.077	.679	-.010	.961
visual_p_19_mean	.112	.556	.130	.486	-.076	.684	-.172	.355	-.163	.406
visual_p_19_median	.062	.746	.079	.673	-.065	.729	-.178	.337	-.141	.475
visual_p_19_min	-.037	.847	.059	.754	-.133	.474	-.179	.334	.156	.429
visual_p_19_offset	.201	.287	.165	.374	-.051	.784	-.189	.310	-.202	.303
visual_p_19_skewness	.000	.999	.046	.808	-.040	.830	.150	.420	-.039	.844
visual_p_19_slop	-.276	.140	-.115	.536	-.097	.604	.109	.558	.140	.476
visual_p_19_std	.186	.326	.155	.404	.178	.339	.088	.638	-.061	.756
visual_p_2_curvature	-.068	.721	.094	.614	.108	.564	-.028	.880	.072	.716
visual_p_2_kurtosis	.146	.443	.063	.738	.164	.379	**.356**	**.049**	-.150	.447
visual_p_2_max	.096	.614	.008	.968	-.011	.952	.084	.654	-.117	.552
visual_p_2_mean	.137	.471	-.103	.582	.056	.763	-.108	.562	.293	.130
visual_p_2_median	.135	.476	-.085	.651	.057	.762	-.079	.671	.262	.178
visual_p_2_min	.023	.903	-.042	.824	.018	.922	-.077	.681	.172	.380
visual_p_2_offset	.145	.444	-.075	.689	.092	.623	-.169	.364	.296	.126
visual_p_2_skewness	.272	.146	.043	.818	.153	.411	.149	.425	-.128	.516
visual_p_2_slop	-.052	.785	-.037	.844	-.163	.380	.159	.392	-.089	.653
visual_p_2_std	.118	.535	-.008	.968	-.140	.451	-.075	.689	-.037	.852
visual_p_20_curvature	.171	.366	.025	.894	-.033	.858	-.096	.607	**-.431**	**.022**
visual_p_20_kurtosis	.304	.103	-.082	.660	-.197	.287	.211	.255	-.072	.715
visual_p_20_max	.159	.401	.039	.835	.112	.547	.229	.216	-.097	.623
visual_p_20_mean	.067	.724	.112	.548	.258	.161	.171	.357	.224	.253
visual_p_20_median	.052	.787	.109	.558	.257	.163	.197	.289	.216	.269
visual_p_20_min	-.129	.497	.025	.893	.158	.396	-.027	.886	.127	.519
visual_p_20_offset	.083	.664	.077	.682	.220	.235	.154	.407	.234	.230
visual_p_20_skewness	-.131	.491	-.107	.568	.075	.688	-.198	.285	-.107	.590
visual_p_20_slop	-.037	.846	.056	.763	.075	.688	-.012	.947	-.044	.823
visual_p_20_std	.046	.810	.284	.121	.138	.459	.112	.548	.026	.896
visual_p_21_curvature	.275	.141	.259	.160	.200	.281	-.229	.215	.239	.221
visual_p_21_kurtosis	-.039	.839	-.126	.498	-.225	.223	-.061	.746	-.253	.194
visual_p_21_max	.019	.919	.301	.100	-.079	.672	.062	.741	-.162	.411
visual_p_21_mean	.004	.985	**.424**	**.017**	.033	.861	-.157	.400	-.080	.686
visual_p_21_median	.005	.977	**.430**	**.016**	.016	.931	-.151	.417	-.065	.742
visual_p_21_min	.000	1.000	.111	.550	.002	.993	-.060	.748	.053	.791
visual_p_21_offset	-.001	.996	**.489**	**.005**	.062	.740	-.031	.867	-.080	.685
visual_p_21_skewness	.003	.987	-.069	.711	.122	.512	.171	.359	-.195	.319

	T1 - SUDS		T2 - SUDS		T3 - SUDS		T4 - SUDS		T5 - SUDS	
	**r**	**p**	**r**	**p**	**r**	**p**	**r**	**p**	**r**	**p**
visual_p_21_slop	.015	.939	-.145	.437	-.112	.549	-.237	.198	.003	.989
visual_p_21_std	.038	.841	.162	.384	.222	.230	.108	.564	-.040	.842
visual_p_22_curvature	-.181	.340	-.186	.315	**-.577**	**.001**	-.019	.919	-.093	.639
visual_p_22_kurtosis	.065	.733	-.040	.830	.102	.586	-.087	.642	-.084	.670
visual_p_22_max	.092	.628	.012	.950	.076	.686	.035	.853	.238	.223
visual_p_22_mean	-.034	.857	.035	.853	.134	.471	.211	.256	**.397**	**.036**
visual_p_22_median	-.040	.832	.033	.859	.110	.557	.247	.181	**.384**	**.044**
visual_p_22_min	-.129	.498	-.099	.597	.261	.157	-.129	.488	.067	.733
visual_p_22_offset	-.002	.993	-.044	.816	.108	.562	.181	.329	.371	.052
visual_p_22_skewness	-.037	.845	-.038	.841	.032	.863	-.259	.160	-.069	.728
visual_p_22_slop	-.081	.671	.115	.539	.062	.741	.085	.650	.084	.669
visual_p_22_std	.149	.433	.133	.475	-.004	.982	.173	.351	.024	.903
visual_p_23_curvature	.045	.812	.068	.717	.303	.098	.153	.411	.212	.278
visual_p_23_kurtosis	.195	.303	-.088	.636	-.219	.236	-.042	.823	-.215	.273
visual_p_23_max	.097	.612	.073	.695	-.158	.395	-.019	.921	-.214	.275
visual_p_23_mean	-.138	.468	.001	.998	-.002	.989	-.125	.504	-.206	.293
visual_p_23_median	-.137	.471	.022	.905	.015	.936	-.156	.403	-.193	.326
visual_p_23_min	-.024	.901	.041	.825	.021	.913	-.116	.533	-.176	.371
visual_p_23_offset	-.050	.792	.101	.589	.060	.749	-.200	.280	-.167	.396
visual_p_23_skewness	.184	.331	.051	.783	.080	.667	.251	.174	-.089	.654
visual_p_23_slop	-.261	.163	-.170	.361	-.174	.350	.229	.214	-.108	.585
visual_p_23_std	.083	.662	.334	.066	.146	.434	.047	.801	.044	.824
visual_p_24_curvature	-.055	.771	.270	.142	.016	.931	.221	.233	.083	.673
visual_p_24_kurtosis	.003	.989	-.025	.892	-.110	.557	-.197	.288	-.239	.220
visual_p_24_max	-.120	.529	-.246	.182	-.089	.633	-.128	.491	-.203	.300
visual_p_24_mean	-.164	.387	**-.587**	**.001**	-.136	.467	-.204	.271	**-.434**	**.021**
visual_p_24_median	-.179	.345	**-.554**	**.001**	-.127	.496	-.219	.237	**-.434**	**.021**
visual_p_24_min	.025	.895	-.097	.605	-.100	.593	-.169	.362	-.261	.179
visual_p_24_offset	-.102	.591	**-.555**	**.001**	-.106	.572	-.184	.321	**-.461**	**.014**
visual_p_24_skewness	.096	.613	.108	.564	-.021	.911	.071	.704	-.014	.945
visual_p_24_slop	-.165	.383	.084	.654	-.058	.757	-.072	.699	-.136	.490
visual_p_24_std	-.038	.844	.084	.654	.100	.593	.089	.634	-.048	.809
visual_p_25_curvature	-.359	.051	-.348	.055	-.289	.115	.161	.387	.361	.059
visual_p_25_kurtosis	.033	.864	-.087	.643	.048	.797	.120	.521	-.280	.150
visual_p_25_max	-.015	.936	.204	.271	-.004	.984	.139	.455	-.245	.209
visual_p_25_mean	-.092	.627	.019	.918	-.071	.705	-.021	.912	-.055	.782
visual_p_25_median	-.099	.604	-.021	.913	-.077	.682	-.021	.911	-.057	.772
visual_p_25_min	-.026	.890	.049	.793	-.093	.618	-.079	.672	.026	.896
visual_p_25_offset	-.084	.658	.018	.925	-.097	.602	.089	.636	-.086	.665
visual_p_25_skewness	.007	.970	.035	.852	-.151	.418	.094	.613	-.214	.273
visual_p_25_slop	.010	.960	-.003	.989	.081	.665	-.207	.264	.056	.779
visual_p_25_std	.128	.500	.281	.125	.068	.716	-.009	.960	-.173	.379
visual_p_26_curvature	.014	.943	.204	.271	-.139	.456	-.134	.473	-.215	.273
visual_p_26_kurtosis	.013	.946	-.074	.693	.066	.723	**.381**	**.034**	-.263	.176
visual_p_26_max	.034	.860	-.076	.684	.053	.776	-.193	.298	-.145	.463
visual_p_26_mean	.083	.661	-.291	.113	.060	.748	-.331	.069	.257	.186
visual_p_26_median	.075	.694	-.282	.125	.070	.708	-.331	.069	.282	.146
visual_p_26_min	.006	.976	-.151	.416	.075	.687	-.275	.134	.087	.659
visual_p_26_offset	.209	.268	-.248	.179	.067	.722	**-.384**	**.033**	.301	.119
visual_p_26_skewness	.025	.896	-.063	.738	.033	.861	-.091	.628	-.272	.162
visual_p_26_slop	-.328	.077	-.027	.885	-.028	.882	.205	.269	-.070	.722
visual_p_26_std	.035	.854	.251	.173	.048	.796	-.111	.552	.033	.867
visual_p_27_curvature	.023	.906	-.271	.141	.219	.237	-.049	.794	.109	.581
visual_p_27_kurtosis	.013	.945	-.074	.694	.061	.743	.249	.176	-.276	.155
visual_p_27_max	-.006	.974	.214	.248	-.074	.692	.206	.267	.098	.620
visual_p_27_mean	.008	.967	.133	.475	-.184	.322	.200	.280	-.340	.077
visual_p_27_median	.019	.920	.110	.555	-.180	.332	.183	.325	-.360	.060
visual_p_27_min	-.031	.869	.071	.704	-.067	.720	-.081	.664	.218	.266
visual_p_27_offset	-.004	.982	.095	.611	-.166	.372	.236	.202	**-.397**	**.037**
visual_p_27_skewness	-.011	.953	.072	.699	-.037	.844	-.042	.823	.315	.102
visual_p_27_slop	.033	.862	.024	.899	-.012	.950	-.048	.799	.197	.315
visual_p_27_std	.064	.735	.239	.195	.127	.495	.200	.280	-.080	.685
visual_p_28_curvature	-.084	.660	.331	.069	-.045	.810	.074	.694	.078	.692
visual_p_28_kurtosis	.015	.936	-.071	.705	.059	.752	.013	.943	-.037	.851
visual_p_28_max	.064	.736	-.037	.844	.063	.735	.046	.805	-.127	.520

	T1 - SUDS		T2 - SUDS		T3 - SUDS		T4 - SUDS		T5 - SUDS	
	r	p	r	p	r	p	r	p	r	p
visual_p_28_mean	.104	.584	-.032	.866	.024	.899	-.128	.493	.304	.116
visual_p_28_median	.069	.716	.008	.965	.069	.714	-.119	.524	.267	.170
visual_p_28_min	.102	.592	-.001	.996	-.030	.874	-.260	.157	.141	.473
visual_p_28_offset	.030	.874	-.038	.841	.064	.733	-.063	.738	**.400**	**.035**
visual_p_28_skewness	.064	.735	-.069	.714	.028	.883	-.017	.928	-.137	.487
visual_p_28_slop	.197	.296	.026	.891	-.119	.522	-.158	.397	-.360	.060
visual_p_28_std	.075	.693	.126	.499	.132	.479	.128	.491	-.006	.976
visual_p_29_curvature	-.079	.678	.087	.640	-.059	.753	.032	.863	-.298	.124
visual_p_29_kurtosis	.037	.846	.047	.800	-.078	.678	.132	.478	-.318	.099
visual_p_29_max	.282	.131	.188	.311	-.129	.490	.207	.264	-.046	.815
visual_p_29_mean	.232	.217	-.318	.081	-.271	.141	-.087	.642	-.136	.492
visual_p_29_median	.173	.362	-.299	.102	-.280	.127	-.110	.556	-.167	.396
visual_p_29_min	.082	.667	-.075	.687	-.063	.736	-.173	.352	.197	.314
visual_p_29_offset	.223	.237	-.334	.067	-.271	.140	.023	.900	-.156	.427
visual_p_29_skewness	.258	.168	.162	.385	.140	.452	.206	.266	.244	.210
visual_p_29_slop	.038	.842	.063	.738	.098	.600	-.222	.229	.101	.608
visual_p_29_std	.120	.528	.112	.548	.152	.414	.173	.353	-.070	.724
visual_p_3_curvature	-.095	.618	**-.384**	**.033**	**.358**	**.048**	.082	.663	.126	.523
visual_p_3_kurtosis	.028	.885	-.016	.931	-.250	.175	-.180	.333	-.026	.897
visual_p_3_max	.016	.935	-.077	.682	.058	.759	-.172	.354	-.289	.136
visual_p_3_mean	.095	.618	-.025	.896	-.269	.143	-.335	.066	-.128	.517
visual_p_3_median	.095	.616	-.061	.743	-.229	.216	**-.387**	**.032**	-.140	.478
visual_p_3_min	.020	.918	.047	.801	-.122	.512	-.159	.393	-.031	.875
visual_p_3_offset	.093	.625	.056	.764	-.288	.116	-.204	.272	-.024	.905
visual_p_3_skewness	-.073	.701	.052	.781	.308	.092	.219	.238	-.009	.965
visual_p_3_slop	-.026	.890	-.168	.366	-.036	.849	-.236	.201	-.235	.228
visual_p_3_std	-.077	.685	-.185	.320	.147	.431	.181	.329	.091	.646
visual_p_30_curvature	-.085	.654	-.237	.200	-.316	.084	.144	.438	-.006	.975
visual_p_30_kurtosis	.360	.051	-.065	.729	.043	.818	-.063	.735	-.245	.209
visual_p_30_max	.171	.365	.219	.237	.047	.801	-.094	.613	-.227	.246
visual_p_30_mean	.212	.262	.174	.349	-.004	.981	-.317	.083	-.052	.792
visual_p_30_median	.180	.342	.141	.451	-.005	.979	-.303	.098	-.056	.778
visual_p_30_min	.069	.719	.005	.977	-.078	.676	-.298	.104	-.215	.272
visual_p_30_offset	.249	.185	.145	.436	-.055	.771	-.102	.586	-.059	.766
visual_p_30_skewness	-.129	.498	-.160	.389	-.007	.969	.112	.548	-.005	.981
visual_p_30_slop	-.141	.459	-.009	.963	.156	.401	-.323	.076	.045	.820
visual_p_30_std	-.060	.754	.235	.204	.062	.739	.045	.811	.003	.989
visual_p_31_curvature	.064	.736	.349	.054	-.184	.322	-.082	.662	-.152	.439
visual_p_31_kurtosis	.021	.914	-.077	.682	-.134	.474	.179	.336	-.208	.288
visual_p_31_max	.076	.690	-.062	.739	.106	.571	.218	.238	-.112	.569
visual_p_31_mean	-.235	.212	-.291	.112	.292	.111	.069	.713	.267	.170
visual_p_31_median	-.249	.184	-.277	.132	.294	.109	.063	.736	.294	.129
visual_p_31_min	-.078	.682	**-.362**	**.045**	.046	.808	-.153	.411	.019	.924
visual_p_31_offset	-.211	.264	-.177	.341	.320	.079	-.133	.475	.326	.091
visual_p_31_skewness	.099	.603	-.073	.697	-.166	.373	-.020	.913	-.198	.312
visual_p_31_slop	-.114	.548	-.189	.309	-.184	.321	**.401**	**.025**	-.079	.690
visual_p_31_std	.167	.377	**.369**	**.041**	.128	.492	.128	.491	.025	.899
visual_p_32_curvature	.032	.868	.226	.220	-.216	.244	.201	.279	-.122	.536
visual_p_32_kurtosis	.022	.908	-.074	.692	.068	.716	-.068	.717	-.188	.339
visual_p_32_max	.119	.530	-.068	.715	.055	.768	-.131	.484	.054	.787
visual_p_32_mean	.203	.281	-.267	.147	-.040	.830	-.228	.218	-.140	.478
visual_p_32_median	.159	.402	-.229	.216	-.001	.997	-.231	.211	-.166	.398
visual_p_32_min	.061	.747	-.301	.100	-.176	.344	-.223	.227	.221	.258
visual_p_32_offset	.266	.155	-.245	.184	-.112	.548	-.224	.225	-.182	.355
visual_p_32_skewness	.099	.601	-.044	.814	.047	.801	.010	.959	.281	.148
visual_p_32_slop	-.200	.290	.046	.806	.183	.326	.026	.891	.092	.640
visual_p_32_std	.135	.478	.180	.332	.048	.797	.000	.999	-.195	.320
visual_p_33_curvature	-.185	.327	**-.402**	**.025**	.162	.383	-.255	.166	.127	.519
visual_p_33_kurtosis	-.098	.605	.029	.878	-.194	.296	.068	.715	-.263	.177
visual_p_33_max	.131	.490	.314	.086	-.120	.521	.183	.325	.001	.996
visual_p_33_mean	.184	.330	**.537**	**.002**	.015	.938	-.112	.548	-.122	.537
visual_p_33_median	.155	.413	**.527**	**.002**	.017	.927	-.078	.678	-.155	.430
visual_p_33_min	-.049	.795	.028	.879	-.131	.482	-.296	.106	.173	.377
visual_p_33_offset	.095	.616	**.543**	**.002**	.066	.725	.124	.505	-.254	.192
visual_p_33_skewness	-.107	.572	.062	.739	-.297	.105	-.115	.539	.055	.781

	T1 - SUDS		T2 - SUDS		T3 - SUDS		T4 - SUDS		T5 - SUDS	
	r	p	r	p	r	p	r	p	r	p
visual_p_33_slop	.280	.134	.084	.654	-.208	.262	-.342	.060	.252	.196
visual_p_33_std	.082	.667	.303	.098	.104	.579	.188	.312	-.142	.472
visual_p_4_curvature	.048	.803	-.192	.301	**-.469**	**.008**	-.044	.815	-.064	.747
visual_p_4_kurtosis	.032	.865	-.225	.223	-.322	.077	-.111	.554	.303	.117
visual_p_4_max	-.138	.466	-.064	.733	-.240	.194	.110	.554	-.011	.954
visual_p_4_mean	-.197	.297	.031	.870	-.176	.343	-.052	.780	-.114	.563
visual_p_4_median	-.199	.292	.073	.696	-.161	.386	-.070	.708	-.086	.662
visual_p_4_min	-.054	.777	-.185	.320	-.266	.148	-.163	.380	.026	.896
visual_p_4_offset	-.181	.339	-.059	.754	-.224	.225	.047	.802	-.120	.542
visual_p_4_skewness	.021	.912	-.248	.178	-.319	.081	-.036	.849	.084	.670
visual_p_4_slop	-.052	.785	.184	.321	.235	.204	-.192	.301	.062	.753
visual_p_4_std	-.044	.818	.078	.677	.114	.543	.238	.197	-.070	.722
visual_p_5_curvature	-.218	.247	-.310	.090	-.037	.843	.292	.111	.338	.079
visual_p_5_kurtosis	-.304	.103	-.011	.952	.013	.944	-.101	.588	-.113	.569
visual_p_5_max	.127	.504	-.043	.820	-.003	.986	.135	.469	.101	.608
visual_p_5_mean	.083	.661	-.047	.800	-.035	.853	.021	.909	.013	.949
visual_p_5_median	.084	.660	-.027	.884	-.047	.802	-.008	.965	-.023	.908
visual_p_5_min	.093	.624	.019	.921	-.155	.406	-.021	.911	.026	.897
visual_p_5_offset	.050	.791	-.100	.591	-.087	.642	.018	.924	.021	.916
visual_p_5_skewness	-.053	.779	.048	.799	.237	.199	.132	.479	.097	.624
visual_p_5_slop	.081	.671	.174	.350	.162	.385	-.015	.937	-.017	.932
visual_p_5_std	.184	.329	.030	.874	.134	.474	.156	.402	.072	.716
visual_p_6_curvature	-.237	.208	-.077	.679	.234	.204	.313	.087	.203	.299
visual_p_6_kurtosis	.003	.989	-.120	.519	-.001	.996	-.223	.228	.086	.664
visual_p_6_max	.023	.904	.002	.990	.032	.865	.331	.069	-.067	.735
visual_p_6_mean	-.041	.831	**.360**	**.046**	-.021	.909	.237	.200	.102	.605
visual_p_6_median	.003	.988	**.381**	**.034**	-.037	.841	.211	.254	.117	.554
visual_p_6_min	-.117	.537	.244	.187	.135	.470	-.030	.875	-.075	.705
visual_p_6_offset	-.113	.552	**.376**	**.037**	-.082	.660	.069	.711	.133	.499
visual_p_6_skewness	.043	.821	-.112	.550	.088	.639	.123	.509	-.126	.522
visual_p_6_slop	.172	.362	-.024	.896	.210	.256	.222	.231	-.121	.541
visual_p_6_std	.123	.517	.182	.326	.087	.641	.264	.151	-.013	.948
visual_p_7_curvature	-.242	.197	-.214	.249	.192	.301	.168	.365	.306	.113
visual_p_7_kurtosis	.045	.812	-.148	.427	.087	.640	-.082	.661	-.196	.318
visual_p_7_max	-.017	.930	.288	.117	-.116	.534	-.082	.662	-.213	.277
visual_p_7_mean	-.078	.680	.324	.075	-.198	.285	-.050	.788	.024	.905
visual_p_7_median	-.028	.883	.312	.088	-.185	.319	-.049	.795	.028	.886
visual_p_7_min	**-.391**	**.032**	.109	.559	-.113	.546	.093	.617	.034	.866
visual_p_7_offset	-.249	.184	.216	.243	-.199	.282	-.062	.739	-.008	.968
visual_p_7_skewness	-.323	.081	-.025	.893	.108	.563	.062	.742	.017	.930
visual_p_7_slop	**.367**	**.046**	.194	.296	.015	.938	.043	.817	.082	.677
visual_p_7_std	.252	.180	.135	.471	.132	.478	.028	.879	-.134	.498
visual_p_8_curvature	-.072	.705	.059	.752	-.233	.208	-.025	.894	**.444**	**.018**
visual_p_8_kurtosis	.030	.877	**.504**	**.004**	-.010	.959	-.169	.365	-.078	.692
visual_p_8_max	.148	.436	.149	.424	-.192	.301	.168	.367	-.059	.765
visual_p_8_mean	.108	.570	-.255	.166	-.040	.831	.288	.116	-.033	.867
visual_p_8_median	.112	.556	-.197	.288	-.030	.873	.265	.150	-.027	.892
visual_p_8_min	-.024	.900	.008	.967	-.076	.684	.037	.845	.013	.947
visual_p_8_offset	.098	.608	-.261	.157	.014	.941	.309	.090	-.147	.456
visual_p_8_skewness	.058	.761	.280	.127	-.144	.441	-.170	.362	-.060	.760
visual_p_8_slop	.012	.949	.052	.780	-.163	.382	-.040	.832	.359	.061
visual_p_8_std	.147	.439	-.194	.296	-.027	.887	.090	.628	.153	.438
visual_p_9_curvature	.194	.304	.325	.074	.105	.574	.084	.653	-.178	.366
visual_p_9_kurtosis	.122	.520	-.099	.597	-.219	.237	-.125	.501	-.184	.348
visual_p_9_max	.038	.841	.061	.744	-.034	.855	.054	.774	.088	.658
visual_p_9_mean	-.067	.726	.183	.323	-.023	.902	-.017	.927	.281	.148
visual_p_9_median	-.115	.545	.202	.277	-.017	.929	-.004	.982	.295	.127
visual_p_9_min	-.056	.768	-.018	.925	-.025	.892	-.264	.152	.202	.303
visual_p_9_offset	-.057	.763	.176	.343	.001	.994	.050	.788	.282	.146
visual_p_9_skewness	.090	.636	-.255	.167	.076	.685	-.130	.485	.048	.807
visual_p_9_slop	-.031	.870	.006	.974	-.118	.528	-.277	.132	.004	.984
visual_p_9_std	-.042	.826	-.174	.348	.058	.757	.173	.352	-.032	.871
visual_p_rx_curvature	.098	.607	-.175	.347	**-.422**	**.018**	-.021	.911	.170	.388
visual_p_rx_kurtosis	.010	.956	-.175	.347	-.024	.900	.182	.326	-.136	.489
visual_p_rx_max	-.057	.766	-.145	.438	-.290	.114	.158	.397	-.172	.381

	T1 - SUDS		T2 - SUDS		T3 - SUDS		T4 - SUDS		T5 - SUDS	
	r	p	r	p	r	p	r	p	r	p
visual_p_rx_mean	-.069	.719	-.110	.558	-.045	.811	.064	.732	-.182	.355
visual_p_rx_median	-.076	.689	-.110	.555	.009	.962	.034	.855	-.197	.316
visual_p_rx_min	.017	.929	-.030	.873	.142	.446	-.255	.166	.142	.471
visual_p_rx_offset	-.087	.647	-.117	.531	-.079	.674	.056	.766	-.260	.182
visual_p_rx_skewness	.129	.496	.097	.604	-.062	.741	.058	.759	.162	.411
visual_p_rx_slop	.073	.700	.031	.870	.081	.665	.001	.997	.209	.285
visual_p_rx_std	-.031	.869	-.043	.818	-.202	.276	.075	.689	-.111	.574
visual_p_ry_curvature	.076	.690	.074	.694	.005	.980	.071	.702	-.169	.390
visual_p_ry_kurtosis	.150	.428	-.275	.134	-.209	.259	-.163	.381	-.174	.375
visual_p_ry_max	.016	.932	.009	.962	-.188	.312	.200	.281	-.098	.619
visual_p_ry_mean	.170	.369	-.077	.682	-.004	.985	.189	.308	-.094	.633
visual_p_ry_median	.225	.232	-.003	.987	-.026	.888	.136	.465	-.102	.606
visual_p_ry_min	-.062	.743	.129	.488	.095	.612	.046	.805	-.009	.965
visual_p_ry_offset	.087	.649	-.229	.215	-.142	.445	-.014	.939	-.162	.411
visual_p_ry_skewness	.142	.455	.105	.575	-.005	.979	.266	.148	-.029	.883
visual_p_ry_slop	.081	.670	.305	.095	.218	.240	.187	.315	.138	.483
visual_p_ry_std	-.009	.964	-.068	.717	.109	.558	.166	.373	.036	.856
visual_p_rz_curvature	-.129	.498	-.294	.108	-.014	.941	.148	.426	.218	.265
visual_p_rz_kurtosis	.030	.875	-.152	.413	-.099	.595	.294	.108	.009	.965
visual_p_rz_max	-.018	.924	.116	.534	-.133	.474	.083	.657	-.142	.470
visual_p_rz_mean	-.063	.743	.265	.150	-.159	.393	.109	.559	.119	.545
visual_p_rz_median	-.003	.987	.263	.153	-.155	.404	.127	.495	.170	.388
visual_p_rz_min	-.023	.904	.334	.066	.055	.768	-.182	.327	.127	.519
visual_p_rz_offset	-.202	.284	.257	.162	-.077	.679	.073	.697	.083	.673
visual_p_rz_skewness	-.232	.217	.095	.612	.091	.626	-.081	.665	-.106	.590
visual_p_rz_slop	.264	.159	-.039	.835	-.165	.376	.088	.639	.021	.916
visual_p_rz_std	.130	.492	.004	.984	.047	.802	.030	.875	-.142	.472
visual_p_scale_curvature	-.195	.301	-.022	.907	.097	.605	.074	.692	.138	.482
visual_p_scale_kurtosis	.206	.274	.227	.219	**-.398**	**.026**	-.094	.615	.237	.225
visual_p_scale_max	.070	.713	.280	.128	-.024	.897	.154	.407	.076	.702
visual_p_scale_mean	.057	.764	**.367**	**.042**	.145	.436	.071	.704	.182	.355
visual_p_scale_median	.056	.771	**.360**	**.047**	.149	.423	.062	.742	.153	.437
visual_p_scale_min	.056	.768	.343	.059	.241	.191	.018	.924	.086	.663
visual_p_scale_offset	.013	.947	.348	.055	.108	.564	.079	.671	.113	.566
visual_p_scale_skewness	.137	.469	.172	.354	-.339	.062	.202	.275	-.023	.908
visual_p_scale_slop	.319	.085	.027	.885	.109	.561	-.055	.767	.134	.498
visual_p_scale_std	.000	1.000	.073	.696	-.062	.741	.157	.398	-.139	.481
**Eye Gaze direction**										
visual_gaze_angle_x_curvature	-.096	.614	.197	.288	.194	.295	-.097	.605	.129	.514
visual_gaze_angle_x_kurtosis	.152	.421	**-.359**	**.047**	-.340	.061	-.146	.432	-.252	.195
visual_gaze_angle_x_max	.014	.940	-.132	.478	-.020	.916	-.049	.793	.075	.705
visual_gaze_angle_x_mean	-.051	.788	-.164	.377	-.045	.811	-.335	.065	.313	.105
visual_gaze_angle_x_median	-.037	.846	-.203	.273	-.061	.743	-.319	.080	.315	.103
visual_gaze_angle_x_min	-.020	.917	-.052	.782	.142	.445	**-.422**	**.018**	.133	.499
visual_gaze_angle_x_offset	-.056	.770	-.041	.828	-.004	.984	-.137	.463	**.399**	**.035**
visual_gaze_angle_x_skewness	-.225	.232	-.080	.669	.132	.478	-.147	.432	-.035	.859
visual_gaze_angle_x_slop	.015	.939	-.315	.084	-.089	.635	-.213	.250	-.217	.266
visual_gaze_angle_x_std	-.055	.772	.112	.548	.175	.347	.280	.127	.188	.338
visual_gaze_angle_y_curvature	-.005	.980	-.238	.198	**-.391**	**.030**	-.005	.979	-.036	.857
visual_gaze_angle_y_kurtosis	.059	.758	-.204	.270	-.026	.889	-.021	.909	-.139	.481
visual_gaze_angle_y_max	.106	.577	.163	.381	-.050	.788	.194	.295	.022	.913
visual_gaze_angle_y_mean	.177	.351	.116	.534	.006	.974	.095	.612	.042	.834
visual_gaze_angle_y_median	.171	.367	.066	.723	-.003	.988	.119	.524	.026	.894
visual_gaze_angle_y_min	.196	.299	.047	.801	.181	.329	-.022	.907	.071	.721
visual_gaze_angle_y_offset	.146	.440	.102	.586	-.039	.836	.083	.655	-.043	.827
visual_gaze_angle_y_skewness	.280	.134	-.078	.676	.047	.802	-.031	.870	-.031	.876
visual_gaze_angle_y_slop	.260	.165	-.009	.960	.181	.331	-.015	.935	.273	.160
visual_gaze_angle_y_std	.059	.759	.259	.159	-.058	.757	.152	.413	.117	.554

Notes. Correlations in bold are significant at $p < .05$.

Table S2

Correlations between acoustic features and stress at T3 and T4

	T3 - SUDS		T4 - SUDS	
	r	p	r	p
**Pitch intensity and frequency**				
audio_pitchFrequency_curvature	-.103	.582	**-.407**	**.023**
audio_pitchFrequency_kurtosis	-.161	.387	-.033	.859
audio_pitchFrequency_max	.159	.392	.168	.367
audio_pitchFrequency_mean	.325	.074	.200	.281
audio_pitchFrequency_median	.196	.291	.208	.262
audio_pitchFrequency_min	.280	.127	.206	.266
audio_pitchFrequency_offset	**.370**	**.041**	.187	.313
audio_pitchFrequency_skewness	-.108	.563	-.129	.490
audio_pitchFrequency_slop	**-.405**	**.024**	-.019	.918
audio_pitchFrequency_std	.250	.175	.085	.650
audio_pitchIntensity_curvature	.007	.971	.111	.552
audio_pitchIntensity_kurtosis	.146	.433	.088	.639
audio_pitchIntensity_max	-.151	.418	-.065	.726
audio_pitchIntensity_mean	.140	.453	.066	.725
audio_pitchIntensity_median	.137	.463	.065	.727
audio_pitchIntensity_min	.166	.372	.028	.883
audio_pitchIntensity_offset	.154	.408	.148	.427
audio_pitchIntensity_skewness	-.107	.566	-.089	.633
audio_pitchIntensity_slop	-.061	.743	-.342	.060
audio_pitchIntensity_std	-.055	.770	-.141	.448
audio_Chromagram0_curvature	.147	.429	.219	.237
audio_Chromagram0_kurtosis	.204	.272	-.064	.733
audio_Chromagram0_max	.000	1.000	.000	1.000
audio_Chromagram0_mean	-.223	.228	-.110	.555
audio_Chromagram0_median	-.158	.396	-.128	.492
audio_Chromagram0_min	.092	.621	.136	.465
audio_Chromagram0_offset	-.315	.084	-.080	.670
audio_Chromagram0_skewness	.109	.560	.059	.753
audio_Chromagram0_slop	.320	.079	-.117	.532
audio_Chromagram0_std	-.305	.095	-.020	.913
audio_Chromagram1_curvature	-.063	.735	.173	.353
audio_Chromagram1_kurtosis	-.103	.581	.181	.330
audio_Chromagram1_max	.000	1.000	.000	1.000
audio_Chromagram1_mean	-.038	.841	-.179	.335
audio_Chromagram1_median	-.112	.547	-.187	.315
audio_Chromagram1_min	-.117	.532	.216	.244
audio_Chromagram1_offset	-.099	.596	-.176	.343
audio_Chromagram1_skewness	.077	.679	.192	.302
audio_Chromagram1_slop	**.362**	**.045**	.068	.714
audio_Chromagram1_std	.346	.057	-.099	.598
audio_Chromagram10_curvature	.271	.140	.013	.946
audio_Chromagram10_kurtosis	-.230	.213	.043	.820
audio_Chromagram10_max	.000	1.000	.000	1.000
audio_Chromagram10_mean	.178	.338	.120	.521
audio_Chromagram10_median	.121	.518	.163	.381
audio_Chromagram10_min	-.063	.738	.038	.840
audio_Chromagram10_offset	.184	.322	.162	.384
audio_Chromagram10_skewness	-.152	.415	-.133	.475
audio_Chromagram10_slop	-.066	.724	-.098	.600
audio_Chromagram10_std	**.474**	**.007**	-.055	.770
audio_Chromagram11_curvature	.140	.452	.013	.943
audio_Chromagram11_kurtosis	.029	.876	-.246	.182
audio_Chromagram11_max	.000	1.000	.000	1.000
audio_Chromagram11_mean	-.264	.151	.065	.730
audio_Chromagram11_median	-.272	.139	.009	.962
audio_Chromagram11_min	.004	.982	-.089	.633
audio_Chromagram11_offset	-.305	.095	.103	.580
audio_Chromagram11_skewness	.186	.316	-.089	.636
audio_Chromagram11_slop	.052	.782	-.043	.820
audio_Chromagram11_std	-.102	.585	.233	.207
audio_Chromagram2_curvature	-.226	.222	.215	.246

	T3 - SUDS		T4 - SUDS	
	**r**	**p**	**r**	**p**
audio_Chromagram2_kurtosis	.153	.411	.032	.862
audio_Chromagram2_max	.000	1.000	.000	1.000
audio_Chromagram2_mean	-.190	.305	-.160	.391
audio_Chromagram2_median	-.254	.168	-.210	.257
audio_Chromagram2_min	-.045	.808	.154	.409
audio_Chromagram2_offset	-.119	.525	-.285	.120
audio_Chromagram2_skewness	.224	.226	.114	.541
audio_Chromagram2_slop	-.151	.417	.330	.070
audio_Chromagram2_std	.068	.717	.050	.790
audio_Chromagram3_curvature	-.203	.273	.210	.258
audio_Chromagram3_kurtosis	-.267	.147	.201	.278
audio_Chromagram3_max	.000	1.000	.000	1.000
audio_Chromagram3_mean	.165	.376	-.097	.602
audio_Chromagram3_median	.108	.563	-.099	.596
audio_Chromagram3_min	-.041	.826	-.018	.923
audio_Chromagram3_offset	.024	.900	-.007	.972
audio_Chromagram3_skewness	-.173	.353	.112	.547
audio_Chromagram3_slop	**.434**	**.015**	-.292	.111
audio_Chromagram3_std	**.446**	**.012**	.003	.989
audio_Chromagram4_curvature	-.218	.238	.069	.713
audio_Chromagram4_kurtosis	.262	.155	.014	.939
audio_Chromagram4_max	.000	1.000	.000	1.000
audio_Chromagram4_mean	-.330	.070	.021	.911
audio_Chromagram4_median	-.346	.057	.027	.885
audio_Chromagram4_min	-.288	.116	-.026	.891
audio_Chromagram4_offset	**-.411**	**.022**	.049	.792
audio_Chromagram4_skewness	.322	.077	-.018	.923
audio_Chromagram4_slop	.285	.120	-.049	.795
audio_Chromagram4_std	-.162	.384	.167	.369
audio_Chromagram5_curvature	-.141	.449	.210	.256
audio_Chromagram5_kurtosis	.056	.764	.237	.200
audio_Chromagram5_max	.000	1.000	.000	1.000
audio_Chromagram5_mean	-.124	.507	-.228	.218
audio_Chromagram5_median	-.239	.195	-.217	.242
audio_Chromagram5_min	**-.370**	**.040**	-.029	.877
audio_Chromagram5_offset	-.025	.895	-.211	.254
audio_Chromagram5_skewness	.176	.342	.218	.238
audio_Chromagram5_slop	-.250	.174	-.111	.553
audio_Chromagram5_std	.208	.262	-.124	.507
audio_Chromagram6_curvature	-.146	.433	.275	.134
audio_Chromagram6_kurtosis	.234	.205	.212	.251
audio_Chromagram6_max	.000	1.000	.000	1.000
audio_Chromagram6_mean	**-.422**	**.018**	-.244	.186
audio_Chromagram6_median	**-.450**	**.011**	-.274	.136
audio_Chromagram6_min	-.198	.286	.232	.210
audio_Chromagram6_offset	**-.448**	**.012**	-.172	.354
audio_Chromagram6_skewness	**.370**	**.040**	.284	.122
audio_Chromagram6_slop	.089	.633	-.204	.271
audio_Chromagram6_std	-.230	.214	-.098	.598
audio_Chromagram7_curvature	-.029	.878	.088	.636
audio_Chromagram7_kurtosis	.234	.206	.097	.605
audio_Chromagram7_max	.000	1.000	.000	1.000
audio_Chromagram7_mean	**-.361**	**.046**	-.254	.168
audio_Chromagram7_median	**-.418**	**.019**	-.265	.149
audio_Chromagram7_min	.071	.706	.029	.876
audio_Chromagram7_offset	**-.455**	**.010**	-.312	.088
audio_Chromagram7_skewness	**.379**	**.036**	.224	.225
audio_Chromagram7_slop	.109	.561	.342	.060
audio_Chromagram7_std	-.105	.575	-.145	.435
audio_Chromagram8_curvature	-.101	.587	.084	.652
audio_Chromagram8_kurtosis	.036	.848	-.063	.736
audio_Chromagram8_max	.000	1.000	.000	1.000
audio_Chromagram8_mean	-.124	.506	.096	.606
audio_Chromagram8_median	**-.156**	**.401**	.071	.705
audio_Chromagram8_min	.269	.143	.338	.063

	T3 - SUDS		T4 - SUDS	
	r	p	r	p
audio_Chromagram8_offset	-.073	.696	.076	.686
audio_Chromagram8_skewness	.124	.507	-.046	.804
audio_Chromagram8_slop	-.215	.246	.138	.458
audio_Chromagram8_std	.005	.979	.145	.436
audio_Chromagram9_curvature	-.017	.927	-.134	.472
audio_Chromagram9_kurtosis	-.143	.444	-.145	.437
audio_Chromagram9_max	.000	1.000	.000	1.000
audio_Chromagram9_mean	.177	.340	.118	.529
audio_Chromagram9_median	.115	.536	.066	.726
audio_Chromagram9_min	.071	.705	.159	.392
audio_Chromagram9_offset	.235	.203	.077	.680
audio_Chromagram9_skewness	-.107	.567	-.101	.587
audio_Chromagram9_slop	-.233	.207	.246	.183
audio_Chromagram9_std	.340	.061	.198	.286
**Formant frequency and bandwidth**				
audio_formantsBandwidth0_curvature	-.001	.997	.146	.435
audio_formantsBandwidth0_kurtosis	.241	.191	.085	.649
audio_formantsBandwidth0_max	-.172	.356	-.032	.863
audio_formantsBandwidth0_mean	-.267	.147	-.119	.525
audio_formantsBandwidth0_median	-.258	.162	-.161	.386
audio_formantsBandwidth0_min	-.260	.158	-.091	.625
audio_formantsBandwidth0_offset	-.278	.130	-.114	.543
audio_formantsBandwidth0_skewness	.182	.326	.087	.640
audio_formantsBandwidth0_slop	-.098	.601	.100	.592
audio_formantsBandwidth0_std	-.355	.050	-.118	.527
audio_formantsBandwidth1_curvature	**.421**	**.018**	.153	.410
audio_formantsBandwidth1_kurtosis	.168	.365	.042	.822
audio_formantsBandwidth1_max	-.185	.319	-.136	.467
audio_formantsBandwidth1_mean	-.228	.218	.141	.451
audio_formantsBandwidth1_median	-.129	.490	.117	.530
audio_formantsBandwidth1_min	-.182	.328	**.389**	**.030**
audio_formantsBandwidth1_offset	-.263	.153	.181	.329
audio_formantsBandwidth1_skewness	.185	.319	.042	.823
audio_formantsBandwidth1_slop	.119	.525	.003	.986
audio_formantsBandwidth1_std	-.299	.102	.139	.456
audio_formantsBandwidth2_curvature	-.039	.834	.196	.289
audio_formantsBandwidth2_kurtosis	-.055	.769	-.129	.490
audio_formantsBandwidth2_max	-.280	.127	.038	.840
audio_formantsBandwidth2_mean	-.049	.793	.149	.425
audio_formantsBandwidth2_median	-.023	.900	.056	.767
audio_formantsBandwidth2_min	-.303	.097	.163	.381
audio_formantsBandwidth2_offset	-.090	.630	.180	.333
audio_formantsBandwidth2_skewness	-.036	.849	-.036	.848
audio_formantsBandwidth2_slop	.059	.752	.086	.646
audio_formantsBandwidth2_std	-.089	.635	.280	.128
audio_formantsBandwidth3_curvature	-.139	.457	-.136	.465
audio_formantsBandwidth3_kurtosis	-.284	.122	-.244	.186
audio_formantsBandwidth3_max	-.070	.707	-.019	.920
audio_formantsBandwidth3_mean	.283	.123	.264	.152
audio_formantsBandwidth3_median	.210	.257	.209	.258
audio_formantsBandwidth3_min	.000	1.000	.025	.892
audio_formantsBandwidth3_offset	.217	.242	.205	.268
audio_formantsBandwidth3_skewness	-.272	.139	-.264	.151
audio_formantsBandwidth3_slop	**.363**	**.044**	.161	.386
audio_formantsBandwidth3_std	.289	.115	.268	.145
audio_formantsBandwidth4_curvature	.206	.266	-.141	.448
audio_formantsBandwidth4_kurtosis	-.155	.405	-.010	.957
audio_formantsBandwidth4_max	-.205	.269	-.128	.492
audio_formantsBandwidth4_mean	.091	.627	-.091	.625
audio_formantsBandwidth4_median	.080	.669	-.138	.458
audio_formantsBandwidth4_min	.000	1.000	.000	1.000
audio_formantsBandwidth4_offset	.076	.686	-.020	.915
audio_formantsBandwidth4_skewness	-.017	.926	.097	.605
audio_formantsBandwidth4_slop	.014	.939	-.083	.657
audio_formantsBandwidth4_std	.076	.686	.072	.701

	T3 - SUDS		T4 - SUDS	
	r	p	r	p
audio_formantsFrequency0_curvature	-.105	.576	.008	.968
audio_formantsFrequency0_kurtosis	-.009	.964	-.257	.163
audio_formantsFrequency0_max	-.210	.256	-.219	.238
audio_formantsFrequency0_mean	.007	.969	.007	.970
audio_formantsFrequency0_median	.061	.744	.048	.799
audio_formantsFrequency0_min	.309	.090	.175	.345
audio_formantsFrequency0_offset	.103	.581	-.016	.930
audio_formantsFrequency0_skewness	-.140	.453	-.303	.098
audio_formantsFrequency0_slop	-.287	.117	.018	.921
audio_formantsFrequency0_std	-.347	.056	-.074	.694
audio_formantsFrequency1_curvature	.288	.116	-.083	.658
audio_formantsFrequency1_kurtosis	-.098	.599	-.201	.278
audio_formantsFrequency1_max	-.281	.126	-.196	.290
audio_formantsFrequency1_mean	-.136	.466	.108	.563
audio_formantsFrequency1_median	-.156	.403	.143	.444
audio_formantsFrequency1_min	.299	.103	.142	.446
audio_formantsFrequency1_offset	-.122	.513	-.011	.955
audio_formantsFrequency1_skewness	.056	.764	-.121	.518
audio_formantsFrequency1_slop	.034	.856	.141	.450
audio_formantsFrequency1_std	**-.376**	**.037**	.155	.404
audio_formantsFrequency2_curvature	.182	.328	-.143	.444
audio_formantsFrequency2_kurtosis	.057	.762	-.063	.735
audio_formantsFrequency2_max	-.273	.137	-.056	.765
audio_formantsFrequency2_mean	-.186	.316	.118	.526
audio_formantsFrequency2_median	-.116	.534	.082	.661
audio_formantsFrequency2_min	.022	.908	.318	.082
audio_formantsFrequency2_offset	-.137	.461	.051	.786
audio_formantsFrequency2_skewness	-.107	.566	-.100	.591
audio_formantsFrequency2_slop	-.178	.339	.021	.913
audio_formantsFrequency2_std	-.196	.291	-.017	.926
audio_formantsFrequency3_curvature	.214	.247	-.206	.266
audio_formantsFrequency3_kurtosis	-.230	.213	-.235	.204
audio_formantsFrequency3_max	-.109	.560	-.125	.503
audio_formantsFrequency3_mean	.045	.812	.057	.762
audio_formantsFrequency3_median	.032	.863	.072	.700
audio_formantsFrequency3_min	.000	1.000	.025	.892
audio_formantsFrequency3_offset	.033	.859	-.034	.854
audio_formantsFrequency3_skewness	.343	.059	.153	.411
audio_formantsFrequency3_slop	.053	.777	.243	.188
audio_formantsFrequency3_std	-.282	.125	.004	.983
audio_formantsFrequency4_curvature	.320	.079	-.128	.493
audio_formantsFrequency4_kurtosis	.023	.901	-.064	.731
audio_formantsFrequency4_max	-.003	.987	-.119	.524
audio_formantsFrequency4_mean	.136	.467	-.048	.796
audio_formantsFrequency4_median	.184	.322	-.007	.972
audio_formantsFrequency4_min	.000	1.000	.000	1.000
audio_formantsFrequency4_offset	.112	.549	-.017	.929
audio_formantsFrequency4_skewness	-.111	.553	.028	.882
audio_formantsFrequency4_slop	.027	.886	.091	.627
audio_formantsFrequency4_std	-.051	.784	.084	.653
**Harmonic to noise ratio**				
audio_HNR_curvature	.062	.742	.055	.768
audio_HNR_kurtosis	-.085	.648	-.022	.908
audio_HNR_max	.268	.145	-.234	.206
audio_HNR_mean	.195	.294	.014	.940
audio_HNR_median	.166	.372	.074	.694
audio_HNR_min	.097	.604	-.243	.188
audio_HNR_offset	.209	.259	.069	.713
audio_HNR_skewness	-.026	.891	-.280	.128
audio_HNR_slop	-.102	.584	-.282	.124
audio_HNR_std	.288	.116	-.139	.457
**Zero crossing rate**				
audio_zeroCrossingRate_curvature	.214	.248	-.036	.847
audio_zeroCrossingRate_kurtosis	**.387**	**.032**	.251	.173
audio_zeroCrossingRate_max	**.369**	**.041**	.067	.721

	T3 - SUDS		T4 - SUDS	
	r	p	r	p
audio_zeroCrossingRate_mean	.040	.829	-.010	.958
audio_zeroCrossingRate_median	.085	.650	.020	.913
audio_zeroCrossingRate_min	.262	.154	.045	.810
audio_zeroCrossingRate_offset	.204	.272	-.041	.825
audio_zeroCrossingRate_skewness	**.363**	**.045**	.260	.159
audio_zeroCrossingRate_slop	**-.505**	**.004**	-.010	.959
audio_zeroCrossingRate_std	-.041	.827	.068	.714
**MFCC**				
audio_MFCC0_curvature	.146	.432	-.142	.447
audio_MFCC0_kurtosis	-.080	.668	-.012	.947
audio_MFCC0_max	-.145	.438	-.106	.570
audio_MFCC0_mean	-.027	.884	-.081	.663
audio_MFCC0_median	-.020	.917	-.031	.869
audio_MFCC0_min	-.013	.946	.134	.473
audio_MFCC0_offset	.103	.582	-.108	.562
audio_MFCC0_skewness	-.021	.911	-.018	.924
audio_MFCC0_slop	**-.411**	**.022**	.036	.849
audio_MFCC0_std	.040	.831	.074	.692
audio_MFCC1_curvature	-.306	.095	.235	.204
audio_MFCC1_kurtosis	.153	.411	.035	.852
audio_MFCC1_max	-.185	.318	-.257	.163
audio_MFCC1_mean	-.064	.731	-.093	.618
audio_MFCC1_median	-.069	.711	-.142	.445
audio_MFCC1_min	-.140	.452	.217	.240
audio_MFCC1_offset	-.030	.872	-.036	.849
audio_MFCC1_skewness	-.120	.520	.182	.327
audio_MFCC1_slop	-.130	.486	-.187	.313
audio_MFCC1_std	-.135	.469	-.191	.303
audio_MFCC10_curvature	-.091	.625	.067	.722
audio_MFCC10_kurtosis	.067	.718	-.098	.600
audio_MFCC10_max	.071	.705	.147	.431
audio_MFCC10_mean	.112	.550	.077	.682
audio_MFCC10_median	.119	.525	.072	.699
audio_MFCC10_min	.172	.356	.263	.153
audio_MFCC10_offset	.058	.758	-.037	.843
audio_MFCC10_skewness	-.037	.843	.086	.645
audio_MFCC10_slop	.223	.229	**.488**	**.005**
audio_MFCC10_std	.072	.700	.046	.806
audio_MFCC11_curvature	.171	.356	-.114	.541
audio_MFCC11_kurtosis	.293	.109	.043	.819
audio_MFCC11_max	.275	.134	-.047	.802
audio_MFCC11_mean	.101	.588	.147	.431
audio_MFCC11_median	.095	.613	.112	.550
audio_MFCC11_min	.137	.463	-.009	.960
audio_MFCC11_offset	.186	.317	.088	.639
audio_MFCC11_skewness	.172	.355	.029	.876
audio_MFCC11_slop	**-.419**	**.019**	.193	.297
audio_MFCC11_std	-.027	.884	-.055	.769
audio_MFCC12_curvature	-.309	.091	.179	.335
audio_MFCC12_kurtosis	.058	.755	-.088	.636
audio_MFCC12_max	-.032	.866	-.007	.969
audio_MFCC12_mean	.094	.616	.002	.990
audio_MFCC12_median	.107	.568	.012	.948
audio_MFCC12_min	.085	.650	.199	.283
audio_MFCC12_offset	.074	.692	.061	.744
audio_MFCC12_skewness	-.170	.361	.051	.785
audio_MFCC12_slop	.084	.653	-.282	.125
audio_MFCC12_std	.041	.828	-.063	.735
audio_MFCC2_curvature	.010	.959	.170	.362
audio_MFCC2_kurtosis	.176	.343	-.072	.702
audio_MFCC2_max	-.071	.705	-.184	.323
audio_MFCC2_mean	-.015	.935	.054	.771
audio_MFCC2_median	.024	.898	.066	.725
audio_MFCC2_min	-.151	.418	-.026	.890
audio_MFCC2_offset	-.159	.394	.112	.549

	T3 - SUDS		T4 - SUDS	
	r	p	r	p
audio_MFCC2_skewness	**-.357**	**.049**	-.122	.512
audio_MFCC2_slop	**.421**	**.018**	-.161	.388
audio_MFCC2_std	.201	.279	.079	.671
audio_MFCC3_curvature	.245	.185	-.204	.271
audio_MFCC3_kurtosis	-.004	.985	-.156	.402
audio_MFCC3_max	-.181	.330	-.184	.322
audio_MFCC3_mean	-.184	.323	.020	.916
audio_MFCC3_median	-.152	.413	.020	.914
audio_MFCC3_min	-.076	.683	.094	.615
audio_MFCC3_offset	-.252	.171	.021	.909
audio_MFCC3_skewness	-.228	.216	-.157	.398
audio_MFCC3_slop	.211	.255	-.083	.656
audio_MFCC3_std	.038	.838	-.093	.619
audio_MFCC4_curvature	.314	.085	.084	.655
audio_MFCC4_kurtosis	-.315	.085	.178	.337
audio_MFCC4_max	-.143	.442	-.244	.186
audio_MFCC4_mean	**-.359**	**.047**	-.033	.859
audio_MFCC4_median	-.350	.053	-.008	.964
audio_MFCC4_min	-.117	.530	.201	.277
audio_MFCC4_offset	**-.362**	**.045**	-.045	.812
audio_MFCC4_skewness	.055	.771	-.085	.649
audio_MFCC4_slop	.091	.626	.153	.412
audio_MFCC4_std	.184	.323	-.169	.363
audio_MFCC5_curvature	.077	.681	.007	.971
audio_MFCC5_kurtosis	.066	.723	-.125	.503
audio_MFCC5_max	**-.382**	**.034**	-.226	.221
audio_MFCC5_mean	-.226	.221	-.225	.223
audio_MFCC5_median	-.220	.234	-.215	.246
audio_MFCC5_min	-.105	.573	.226	.222
audio_MFCC5_offset	-.264	.151	-.211	.256
audio_MFCC5_skewness	-.123	.510	-.132	.479
audio_MFCC5_slop	.244	.186	-.084	.652
audio_MFCC5_std	-.188	.311	-.211	.254
audio_MFCC6_curvature	-.268	.145	.128	.494
audio_MFCC6_kurtosis	-.136	.466	-.249	.177
audio_MFCC6_max	-.025	.895	.211	.255
audio_MFCC6_mean	-.070	.709	-.083	.655
audio_MFCC6_median	-.084	.652	-.100	.593
audio_MFCC6_min	-.015	.937	-.069	.714
audio_MFCC6_offset	-.129	.489	-.052	.780
audio_MFCC6_skewness	.134	.472	.137	.462
audio_MFCC6_slop	.314	.086	.058	.756
audio_MFCC6_std	.056	.767	.193	.297
audio_MFCC7_curvature	.031	.867	**.399**	**.026**
audio_MFCC7_kurtosis	-.079	.674	**-.441**	**.013**
audio_MFCC7_max	-.099	.596	-.339	.062
audio_MFCC7_mean	-.182	.328	-.021	.913
audio_MFCC7_median	-.176	.345	-.034	.857
audio_MFCC7_min	.052	.781	.130	.487
audio_MFCC7_offset	-.165	.376	-.049	.794
audio_MFCC7_skewness	.113	.544	.015	.937
audio_MFCC7_slop	-.044	.814	.242	.190
audio_MFCC7_std	-.022	.905	.001	.994
audio_MFCC8_curvature	-.174	.350	.067	.722
audio_MFCC8_kurtosis	.166	.371	.132	.478
audio_MFCC8_max	.077	.679	.011	.955
audio_MFCC8_mean	-.255	.166	-.105	.574
audio_MFCC8_median	-.242	.189	-.087	.640
audio_MFCC8_min	.007	.971	.005	.978
audio_MFCC8_offset	-.213	.249	-.016	.932
audio_MFCC8_skewness	.059	.751	.154	.407
audio_MFCC8_slop	-.030	.872	-.253	.170
audio_MFCC8_std	.090	.630	.000	.998
audio_MFCC9_curvature	.051	.784	.251	.173
audio_MFCC9_kurtosis	.171	.358	.024	.898

	T3 - SUDS		T4 - SUDS	
	r	p	r	p
audio_MFCC9_max	-.284	.122	.008	.968
audio_MFCC9_mean	-.268	.145	.067	.722
audio_MFCC9_median	-.264	.152	.088	.637
audio_MFCC9_min	-.222	.229	.205	.268
audio_MFCC9_offset	-.274	.135	.075	.688
audio_MFCC9_skewness	-.055	.769	-.051	.785
audio_MFCC9_slop	.017	.928	-.044	.815
audio_MFCC9_std	-.154	.408	-.060	.749
audio_MFSC0_curvature	.007	.971	-.137	.463
audio_MFSC0_kurtosis	-.156	.401	.093	.618
audio_MFSC0_max	-.202	.277	-.018	.923
audio_MFSC0_mean	-.039	.833	.004	.983
audio_MFSC0_median	-.048	.796	-.016	.932
audio_MFSC0_min	-.086	.647	.083	.657
audio_MFSC0_offset	.049	.792	-.030	.874
audio_MFSC0_skewness	-.147	.431	.084	.653
audio_MFSC0_slop	-.239	.196	-.012	.950
audio_MFSC0_std	-.060	.749	.038	.838
audio_MFSC1_curvature	.033	.860	-.103	.581
audio_MFSC1_kurtosis	.315	.084	.292	.111
audio_MFSC1_max	**.416**	**.020**	.033	.859
audio_MFSC1_mean	.168	.365	.022	.905
audio_MFSC1_median	.093	.619	.010	.957
audio_MFSC1_min	**.403**	**.025**	.039	.836
audio_MFSC1_offset	.312	.088	-.023	.901
audio_MFSC1_skewness	.322	.078	.250	.175
audio_MFSC1_slop	**-.399**	**.026**	.062	.742
audio_MFSC1_std	.270	.142	.055	.767
audio_MFSC10_curvature	.255	.166	-.130	.485
audio_MFSC10_kurtosis	-.087	.642	-.138	.460
audio_MFSC10_max	-.080	.668	-.034	.855
audio_MFSC10_mean	-.024	.899	.003	.987
audio_MFSC10_median	-.026	.889	-.018	.925
audio_MFSC10_min	.152	.413	-.048	.797
audio_MFSC10_offset	.024	.898	-.057	.759
audio_MFSC10_skewness	-.051	.787	.016	.932
audio_MFSC10_slop	-.281	.126	.062	.739
audio_MFSC10_std	-.022	.905	.065	.730
audio_MFSC11_curvature	.265	.150	-.091	.625
audio_MFSC11_kurtosis	-.193	.299	-.139	.457
audio_MFSC11_max	-.221	.232	-.299	.103
audio_MFSC11_mean	-.055	.769	-.066	.724
audio_MFSC11_median	-.057	.761	-.059	.753
audio_MFSC11_min	.004	.982	.154	.409
audio_MFSC11_offset	.068	.717	-.136	.466
audio_MFSC11_skewness	-.200	.281	-.120	.521
audio_MFSC11_slop	-.311	.088	.059	.752
audio_MFSC11_std	-.064	.734	-.088	.640
audio_MFSC2_curvature	-.016	.932	-.041	.827
audio_MFSC2_kurtosis	.203	.273	-.177	.341
audio_MFSC2_max	.222	.231	-.053	.777
audio_MFSC2_mean	.237	.200	.094	.616
audio_MFSC2_median	.204	.272	.085	.651
audio_MFSC2_min	.208	.261	-.040	.832
audio_MFSC2_offset	**.395**	**.028**	.042	.821
audio_MFSC2_skewness	.199	.282	-.124	.505
audio_MFSC2_slop	**-.484**	**.006**	.085	.649
audio_MFSC2_std	.288	.116	.099	.597
audio_MFSC3_curvature	-.020	.915	-.046	.805
audio_MFSC3_kurtosis	.212	.252	-.303	.097
audio_MFSC3_max	.176	.343	-.141	.450
audio_MFSC3_mean	.086	.644	.077	.680
audio_MFSC3_median	.062	.739	.008	.968
audio_MFSC3_min	.200	.281	.176	.343
audio_MFSC3_offset	.267	.146	.010	.957

	T3 - SUDS		T4 - SUDS	
	r	p	r	p
audio_MFSC3_skewness	.155	.406	-.266	.148
audio_MFSC3_slop	**-.540**	**.002**	.125	.501
audio_MFSC3_std	.114	.540	.079	.673
audio_MFSC4_curvature	-.042	.822	-.136	.465
audio_MFSC4_kurtosis	-.047	.803	.085	.649
audio_MFSC4_max	.015	.937	-.022	.907
audio_MFSC4_mean	.063	.735	.038	.839
audio_MFSC4_median	.048	.796	-.020	.915
audio_MFSC4_min	.109	.559	.145	.435
audio_MFSC4_offset	.263	.153	-.036	.846
audio_MFSC4_skewness	-.058	.758	.119	.525
audio_MFSC4_slop	**-.564**	**.001**	.148	.426
audio_MFSC4_std	.067	.721	.083	.659
audio_MFSC5_curvature	.057	.760	-.134	.471
audio_MFSC5_kurtosis	.054	.773	.107	.566
audio_MFSC5_max	-.014	.938	-.126	.499
audio_MFSC5_mean	-.020	.917	.027	.885
audio_MFSC5_median	-.053	.777	.003	.988
audio_MFSC5_min	.258	.162	.320	.080
audio_MFSC5_offset	.168	.366	-.036	.845
audio_MFSC5_skewness	.126	.498	.104	.577
audio_MFSC5_slop	**-.495**	**.005**	.121	.516
audio_MFSC5_std	.021	.909	.026	.891
audio_MFSC6_curvature	.127	.497	-.125	.502
audio_MFSC6_kurtosis	.142	.445	.109	.558
audio_MFSC6_max	.087	.643	-.002	.990
audio_MFSC6_mean	-.192	.301	.012	.948
audio_MFSC6_median	-.215	.246	-.003	.987
audio_MFSC6_min	.292	.111	.186	.315
audio_MFSC6_offset	-.029	.877	-.029	.876
audio_MFSC6_skewness	.177	.341	.069	.713
audio_MFSC6_slop	**-.479**	**.006**	.052	.782
audio_MFSC6_std	-.113	.544	.022	.905
audio_MFSC7_curvature	.146	.434	-.126	.499
audio_MFSC7_kurtosis	.170	.360	.201	.278
audio_MFSC7_max	.115	.539	.118	.528
audio_MFSC7_mean	-.201	.277	.050	.789
audio_MFSC7_median	-.202	.275	.017	.929
audio_MFSC7_min	.047	.801	-.021	.912
audio_MFSC7_offset	-.061	.745	-.015	.935
audio_MFSC7_skewness	.217	.241	.201	.278
audio_MFSC7_slop	**-.471**	**.007**	.085	.651
audio_MFSC7_std	-.119	.522	.096	.607
audio_MFSC8_curvature	**.452**	**.011**	-.154	.407
audio_MFSC8_kurtosis	-.013	.947	.146	.433
audio_MFSC8_max	-.128	.492	.135	.468
audio_MFSC8_mean	.032	.865	.083	.657
audio_MFSC8_median	.047	.802	.069	.711
audio_MFSC8_min	.199	.283	.303	.097
audio_MFSC8_offset	.123	.511	-.010	.955
audio_MFSC8_skewness	-.019	.920	.123	.510
audio_MFSC8_slop	**-.356**	**.049**	.140	.453
audio_MFSC8_std	.006	.975	.138	.461
audio_MFSC9_curvature	**.388**	**.031**	-.163	.381
audio_MFSC9_kurtosis	.133	.474	.076	.683
audio_MFSC9_max	.065	.727	.077	.679
audio_MFSC9_mean	-.015	.935	.034	.855
audio_MFSC9_median	-.007	.968	.008	.966
audio_MFSC9_min	.032	.863	.045	.809
audio_MFSC9_offset	.044	.816	-.030	.872
audio_MFSC9_skewness	.177	.342	.138	.458
audio_MFSC9_slop	-.328	.072	.076	.686
audio_MFSC9_std	-.002	.993	.099	.595
**LFCC**				
audio_LFCC0_curvature	-.055	.768	-.165	.375

	T3 - SUDS		T4 - SUDS	
	**r**	**p**	**r**	**p**
audio_LFCC0_kurtosis	.130	.487	-.129	.489
audio_LFCC0_max	.104	.579	.086	.645
audio_LFCC0_mean	-.007	.970	-.085	.648
audio_LFCC0_median	-.004	.985	-.075	.689
audio_LFCC0_min	-.005	.978	.064	.732
audio_LFCC0_offset	.098	.599	-.100	.592
audio_LFCC0_skewness	-.012	.950	.061	.746
audio_LFCC0_slop	-.350	.054	-.052	.782
audio_LFCC0_std	.176	.343	.179	.334
audio_LFCC1_curvature	**-.392**	**.029**	.183	.324
audio_LFCC1_kurtosis	.062	.740	.129	.488
audio_LFCC1_max	-.143	.442	-.051	.784
audio_LFCC1_mean	-.035	.851	-.021	.909
audio_LFCC1_median	-.055	.769	-.046	.806
audio_LFCC1_min	.044	.812	.116	.535
audio_LFCC1_offset	.006	.976	.013	.945
audio_LFCC1_skewness	.025	.894	-.003	.986
audio_LFCC1_slop	-.089	.635	-.074	.691
audio_LFCC1_std	-.223	.228	-.225	.225
audio_LFCC10_curvature	.180	.331	.028	.880
audio_LFCC10_kurtosis	-.197	.287	.322	.077
audio_LFCC10_max	-.050	.789	-.171	.357
audio_LFCC10_mean	.014	.939	.092	.621
audio_LFCC10_median	-.009	.964	.087	.643
audio_LFCC10_min	.005	.978	**-.373**	**.039**
audio_LFCC10_offset	.067	.722	-.065	.728
audio_LFCC10_skewness	.275	.134	-.350	.054
audio_LFCC10_slop	-.133	.476	.316	.084
audio_LFCC10_std	-.027	.884	.060	.748
audio_LFCC11_curvature	-.011	.954	.023	.904
audio_LFCC11_kurtosis	.036	.847	.141	.450
audio_LFCC11_max	-.053	.778	.010	.959
audio_LFCC11_mean	.069	.714	**.358**	**.048**
audio_LFCC11_median	.056	.764	**.363**	**.045**
audio_LFCC11_min	.187	.313	.233	.207
audio_LFCC11_offset	.063	.735	**.430**	**.016**
audio_LFCC11_skewness	.200	.281	-.142	.446
audio_LFCC11_slop	-.007	.971	-.158	.395
audio_LFCC11_std	-.201	.278	.031	.869
audio_LFCC2_curvature	.066	.724	.269	.144
audio_LFCC2_kurtosis	.139	.456	-.017	.929
audio_LFCC2_max	-.206	.266	-.259	.159
audio_LFCC2_mean	-.013	.944	.000	1.000
audio_LFCC2_median	-.035	.850	.001	.996
audio_LFCC2_min	-.138	.459	.130	.487
audio_LFCC2_offset	.048	.797	-.006	.973
audio_LFCC2_skewness	.013	.943	-.111	.551
audio_LFCC2_slop	-.263	.153	.087	.641
audio_LFCC2_std	-.132	.480	-.087	.643
audio_LFCC3_curvature	-.353	.051	.242	.190
audio_LFCC3_kurtosis	.314	.086	**.456**	**.010**
audio_LFCC3_max	.037	.844	-.127	.496
audio_LFCC3_mean	.107	.567	.016	.933
audio_LFCC3_median	.102	.586	-.008	.966
audio_LFCC3_min	-.257	.163	.076	.686
audio_LFCC3_offset	.009	.962	.121	.515
audio_LFCC3_skewness	-.189	.308	-.183	.324
audio_LFCC3_slop	**.375**	**.038**	-.296	.106
audio_LFCC3_std	.018	.924	-.242	.190
audio_LFCC4_curvature	-.162	.384	.047	.803
audio_LFCC4_kurtosis	-.117	.529	.137	.462
audio_LFCC4_max	.145	.437	-.079	.671
audio_LFCC4_mean	.339	.062	-.128	.492
audio_LFCC4_median	.327	.072	-.122	.513
audio_LFCC4_min	.038	.839	-.156	.402

	T3 - SUDS		T4 - SUDS	
	r	p	r	p
audio_LFCC4_offset	.302	.098	-.078	.678
audio_LFCC4_skewness	-.144	.440	-.126	.500
audio_LFCC4_slop	.135	.470	-.264	.152
audio_LFCC4_std	.247	.180	.027	.884
audio_LFCC5_curvature	-.045	.809	.061	.745
audio_LFCC5_kurtosis	.012	.948	-.097	.604
audio_LFCC5_max	.219	.237	.077	.679
audio_LFCC5_mean	.079	.673	.085	.651
audio_LFCC5_median	.104	.577	.109	.561
audio_LFCC5_min	-.215	.245	.000	.998
audio_LFCC5_offset	.046	.806	.194	.296
audio_LFCC5_skewness	-.246	.183	.032	.865
audio_LFCC5_slop	.047	.802	**-.378**	**.036**
audio_LFCC5_std	.200	.280	.160	.390
audio_LFCC6_curvature	-.271	.140	.159	.393
audio_LFCC6_kurtosis	-.070	.709	.011	.955
audio_LFCC6_max	.100	.591	-.006	.974
audio_LFCC6_mean	.079	.671	.231	.212
audio_LFCC6_median	.069	.713	.216	.244
audio_LFCC6_min	.108	.561	.335	.065
audio_LFCC6_offset	-.052	.781	.294	.109
audio_LFCC6_skewness	-.021	.911	.066	.725
audio_LFCC6_slop	**.383**	**.033**	-.248	.179
audio_LFCC6_std	.031	.870	-.112	.550
audio_LFCC7_curvature	.023	.903	.175	.347
audio_LFCC7_kurtosis	-.047	.800	.028	.880
audio_LFCC7_max	.068	.718	-.050	.790
audio_LFCC7_mean	.286	.119	.058	.755
audio_LFCC7_median	.293	.110	.080	.670
audio_LFCC7_min	.208	.262	.160	.390
audio_LFCC7_offset	.207	.265	-.062	.738
audio_LFCC7_skewness	-.022	.905	-.201	.278
audio_LFCC7_slop	.227	.219	**.387**	**.032**
audio_LFCC7_std	.011	.951	-.072	.701
audio_LFCC8_curvature	.081	.666	-.189	.310
audio_LFCC8_kurtosis	.010	.957	.096	.606
audio_LFCC8_max	.056	.767	-.328	.072
audio_LFCC8_mean	.092	.621	-.055	.769
audio_LFCC8_median	.116	.534	-.017	.926
audio_LFCC8_min	-.228	.217	-.264	.151
audio_LFCC8_offset	.047	.800	-.071	.703
audio_LFCC8_skewness	-.278	.131	**-.425**	**.017**
audio_LFCC8_slop	.006	.972	.149	.423
audio_LFCC8_std	.207	.265	.059	.754
audio_LFCC9_curvature	.092	.621	-.204	.271
audio_LFCC9_kurtosis	-.345	.058	-.046	.806
audio_LFCC9_max	-.183	.324	-.118	.528
audio_LFCC9_mean	-.196	.290	-.077	.679
audio_LFCC9_median	-.188	.310	-.096	.608
audio_LFCC9_min	.006	.974	.103	.581
audio_LFCC9_offset	-.199	.284	-.051	.784
audio_LFCC9_skewness	-.050	.790	.226	.222
audio_LFCC9_slop	-.065	.727	-.113	.547
audio_LFCC9_std	.137	.461	-.054	.774
**Other**				
audio_polynomialFeatures0_curvature	-.137	.463	.132	.479
audio_polynomialFeatures0_kurtosis	.025	.892	.205	.269
audio_polynomialFeatures0_max	-.029	.876	-.074	.694
audio_polynomialFeatures0_mean	.032	.866	.007	.972
audio_polynomialFeatures0_median	.024	.897	-.022	.908
audio_polynomialFeatures0_min	.134	.472	.106	.570
audio_polynomialFeatures0_offset	-.133	.475	.070	.708
audio_polynomialFeatures0_skewness	-.033	.860	-.088	.637
audio_polynomialFeatures0_slop	**.474**	**.007**	-.131	.484
audio_polynomialFeatures0_std	-.028	.881	.001	.998

	T3 - SUDS		T4 - SUDS	
	r	p	r	p
audio_polynomialFeatures1_curvature	.140	.454	-.132	.478
audio_polynomialFeatures1_kurtosis	.017	.929	.202	.277
audio_polynomialFeatures1_max	-.166	.372	-.097	.603
audio_polynomialFeatures1_mean	-.032	.866	-.006	.974
audio_polynomialFeatures1_median	-.024	.898	.023	.902
audio_polynomialFeatures1_min	.026	.891	.063	.737
audio_polynomialFeatures1_offset	.133	.476	-.070	.708
audio_polynomialFeatures1_skewness	.030	.873	.085	.648
audio_polynomialFeatures1_slop	**-.476**	**.007**	.132	.477
audio_polynomialFeatures1_std	-.027	.884	.001	.996
audio_RMS_curvature	.153	.411	-.144	.438
audio_RMS_kurtosis	-.045	.810	.241	.192
audio_RMS_max	.087	.641	-.001	.994
audio_RMS_mean	.041	.825	.022	.907
audio_RMS_median	.023	.901	.057	.760
audio_RMS_min	.099	.594	.088	.638
audio_RMS_offset	.209	.260	-.043	.820
audio_RMS_skewness	.013	.943	.110	.555
audio_RMS_slop	**-.471**	**.007**	.084	.653
audio_RMS_std	.086	.646	.024	.900
audio_spectralBandwidth_curvature	.298	.104	-.122	.515
audio_spectralBandwidth_kurtosis	.234	.204	.095	.610
audio_spectralBandwidth_max	.215	.245	-.160	.391
audio_spectralBandwidth_mean	-.059	.753	.091	.627
audio_spectralBandwidth_median	-.042	.824	.126	.500
audio_spectralBandwidth_min	.081	.663	.351	.053
audio_spectralBandwidth_offset	-.074	.691	.041	.828
audio_spectralBandwidth_skewness	.151	.418	-.007	.972
audio_spectralBandwidth_slop	.092	.621	.140	.453
audio_spectralBandwidth_std	-.069	.714	-.149	.423
audio_spectralCentroid_curvature	.338	.063	-.150	.420
audio_spectralCentroid_kurtosis	.252	.171	.196	.291
audio_spectralCentroid_max	-.012	.951	-.179	.336
audio_spectralCentroid_mean	-.059	.752	-.001	.997
audio_spectralCentroid_median	-.026	.888	.047	.802
audio_spectralCentroid_min	-.095	.612	.115	.539
audio_spectralCentroid_offset	.043	.818	-.085	.650
audio_spectralCentroid_skewness	.183	.324	.172	.354
audio_spectralCentroid_slop	-.340	.061	.161	.388
audio_spectralCentroid_std	-.136	.467	-.142	.445
audio_spectralContrast0_curvature	.321	.078	.152	.414
audio_spectralContrast0_kurtosis	-.245	.184	-.162	.383
audio_spectralContrast0_max	.047	.801	.097	.603
audio_spectralContrast0_mean	-.325	.075	-.279	.128
audio_spectralContrast0_median	-.306	.094	-.265	.150
audio_spectralContrast0_min	-.295	.108	-.115	.540
audio_spectralContrast0_offset	-.320	.079	-.225	.225
audio_spectralContrast0_skewness	-.269	.143	-.169	.363
audio_spectralContrast0_slop	.066	.723	-.209	.260
audio_spectralContrast0_std	.335	.066	.352	.052
audio_spectralContrast1_curvature	-.067	.720	-.104	.578
audio_spectralContrast1_kurtosis	**-.451**	**.011**	-.174	.350
audio_spectralContrast1_max	-.106	.570	-.221	.231
audio_spectralContrast1_mean	.228	.217	.070	.707
audio_spectralContrast1_median	.222	.231	.052	.780
audio_spectralContrast1_min	.069	.714	**.358**	**.048**
audio_spectralContrast1_offset	.259	.160	.093	.619
audio_spectralContrast1_skewness	-.334	.066	.000	1.000
audio_spectralContrast1_slop	-.211	.256	-.214	.248
audio_spectralContrast1_std	.347	.056	.224	.226
audio_spectralContrast2_curvature	.109	.561	-.203	.272
audio_spectralContrast2_kurtosis	-.262	.155	-.156	.401
audio_spectralContrast2_max	.035	.851	.060	.748
audio_spectralContrast2_mean	.287	.117	.118	.526
audio_spectralContrast2_median	.289	.115	.135	.468

	T3 - SUDS		T4 - SUDS	
	**r**	**p**	**r**	**p**
audio_spectralContrast2_min	.166	.373	.170	.361
audio_spectralContrast2_offset	.305	.095	.126	.498
audio_spectralContrast2_skewness	-.307	.093	-.211	.254
audio_spectralContrast2_slop	-.188	.310	-.295	.107
audio_spectralContrast2_std	.219	.237	.150	.422
audio_spectralContrast3_curvature	-.049	.792	-.069	.713
audio_spectralContrast3_kurtosis	-.149	.425	-.194	.296
audio_spectralContrast3_max	.055	.768	.063	.735
audio_spectralContrast3_mean	.022	.904	.133	.477
audio_spectralContrast3_median	.036	.847	.129	.490
audio_spectralContrast3_min	.211	.254	-.123	.511
audio_spectralContrast3_offset	.054	.771	.098	.601
audio_spectralContrast3_skewness	-.152	.415	-.176	.344
audio_spectralContrast3_slop	-.201	.278	-.108	.562
audio_spectralContrast3_std	-.015	.936	.159	.394
audio_spectralContrast4_curvature	-.174	.349	-.025	.894
audio_spectralContrast4_kurtosis	-.312	.088	-.280	.127
audio_spectralContrast4_max	-.017	.929	-.038	.841
audio_spectralContrast4_mean	.334	.066	.161	.388
audio_spectralContrast4_median	.327	.073	.145	.438
audio_spectralContrast4_min	.178	.339	.083	.655
audio_spectralContrast4_offset	.297	.104	.148	.426
audio_spectralContrast4_skewness	-.239	.196	-.134	.471
audio_spectralContrast4_slop	-.058	.756	-.159	.394
audio_spectralContrast4_std	.289	.114	.236	.201
audio_spectralContrast5_curvature	.009	.963	-.002	.992
audio_spectralContrast5_kurtosis	.205	.270	.004	.984
audio_spectralContrast5_max	-.127	.497	-.119	.524
audio_spectralContrast5_mean	-.107	.567	-.042	.822
audio_spectralContrast5_median	-.123	.509	-.039	.835
audio_spectralContrast5_min	.140	.453	.157	.400
audio_spectralContrast5_offset	.027	.886	-.050	.789
audio_spectralContrast5_skewness	.219	.237	.024	.897
audio_spectralContrast5_slop	**-.425**	**.017**	-.050	.791
audio_spectralContrast5_std	-.040	.830	.003	.986
audio_spectralContrast6_curvature	.258	.162	-.179	.335
audio_spectralContrast6_kurtosis	-.015	.935	.044	.813
audio_spectralContrast6_max	-.035	.851	-.153	.410
audio_spectralContrast6_mean	.016	.931	-.038	.839
audio_spectralContrast6_median	.023	.903	-.021	.911
audio_spectralContrast6_min	.109	.560	.027	.883
audio_spectralContrast6_offset	.090	.632	-.060	.750
audio_spectralContrast6_skewness	-.068	.717	-.014	.941
audio_spectralContrast6_slop	-.315	.085	-.056	.765
audio_spectralContrast6_std	-.121	.518	-.106	.570
audio_spectralFlatness_curvature	.109	.559	-.176	.344
audio_spectralFlatness_kurtosis	.257	.163	-.076	.683
audio_spectralFlatness_max	.082	.661	-.312	.088
audio_spectralFlatness_mean	-.189	.310	-.114	.542
audio_spectralFlatness_median	-.094	.616	.125	.503
audio_spectralFlatness_min	.047	.802	.252	.171
audio_spectralFlatness_offset	-.143	.442	-.179	.335
audio_spectralFlatness_skewness	.295	.107	-.035	.854
audio_spectralFlatness_slop	-.037	.843	.185	.319
audio_spectralFlatness_std	-.115	.539	-.237	.198
audio_spectralFlux_curvature	.261	.156	-.214	.248
audio_spectralFlux_kurtosis	.050	.791	.114	.541
audio_spectralFlux_max	.244	.186	-.036	.846
audio_spectralFlux_mean	.208	.262	-.020	.916
audio_spectralFlux_median	.173	.351	.061	.744
audio_spectralFlux_min	-.013	.947	**-.386**	**.032**
audio_spectralFlux_offset	.284	.122	-.071	.703
audio_spectralFlux_skewness	.006	.975	.055	.767
audio_spectralFlux_slop	-.348	.055	.233	.207
audio_spectralFlux_std	.247	.180	-.174	.350

	T3 - SUDS		T4 - SUDS	
	**r**	**p**	**r**	**p**
audio_spectralRolloff_curvature	.304	.097	-.173	.352
audio_spectralRolloff_kurtosis	.234	.206	.093	.617
audio_spectralRolloff_max	.289	.115	-.208	.261
audio_spectralRolloff_mean	-.148	.427	-.040	.831
audio_spectralRolloff_median	-.143	.443	.027	.885
audio_spectralRolloff_min	.062	.739	-.008	.965
audio_spectralRolloff_offset	-.025	.892	-.134	.471
audio_spectralRolloff_skewness	.178	.337	.038	.840
audio_spectralRolloff_slop	-.319	.080	.168	.366
audio_spectralRolloff_std	-.108	.564	-.122	.514
audio_intensity_curvature	.101	.587	-.113	.546
audio_intensity_kurtosis	-.117	.532	.032	.864
audio_intensity_max	.073	.695	.021	.910
audio_intensity_mean	.034	.857	-.067	.720
audio_intensity_median	.031	.868	-.022	.905
audio_intensity_min	.185	.319	.132	.479
audio_intensity_offset	.161	.387	-.072	.701
audio_intensity_skewness	.023	.902	.029	.879

Notes. Correlations in bold are significant at $p < .05$.

Table S3

Correlations between verbal features and stress at T3

	T3 - SUDS	
	**r**	**p**
“baan” = job	.154	.409
“beetje” = a bit	-.158	.395
“belangrijk” = important	.139	.457
“bijvoorbeeld” = for example	.027	.885
“denken” = to think	.054	.775
“deze” = this	-.217	.241
“ding” = thing	-.104	.577
“doen” = to do	-.051	.786
“één” = one	.068	.718
“eigenlijk” = actually	-.125	.504
“erg” = very	.089	.632
“ervaring” = experience	.092	.622
“gaan” = to go	**-.368**	**.042**
“geschikt” = suitable	-.095	.613
“geven” = to give	-.134	.471
“goed” = good	.334	.066
“graag” = gladly	.278	.130
“heel” = very	.061	.744
“helpen” = to help	-.105	.572
“hier” = here	.090	.632
“houden” = to hold	-.287	.117
“iemand” = someone	.244	.185
“iets” = something	.030	.873
“interessant” = interesting	.045	.810
“ja” = yes	**.363**	**.045**
“jaar” = year	-.062	.740
“kijken” = to look	-.277	.131
“kind” = child	-.257	.163
“komen” = to come	-.120	.522
“krijgen” = to get	-.127	.497
“leren” = to learn	-.052	.782
“leuk” = nice	-.184	.321
“leven” = life	-.306	.094
“lijken” = to resemble	-.101	.589
“maken” = to make	-.190	.306
“mee” = along	-.170	.360
“mens” = man	.014	.938
“natuurlijk” = naturally	-.156	.403
“nou” = well	-.192	.301
“oké” = okay	.041	.827
“persoon” = person	-.213	.250
“praten” = to talk	-.130	.484
“probleem” = problem	-.215	.246
“psychologie” = psychology	-.080	.668
“psycholoog” = psychologist	.203	.273
“punt” = point	.121	.518
“soms” = sometimes	.341	.061
“studie” = study	.032	.863
“tijd” = time	.035	.853
“toe” = to	-.038	.839
“vaak” = often	.151	.417
“veel” = a lot of	.232	.210
“vertellen” = to tell	.102	.585
“vinden” = to find	-.078	.675
“voelen” = to feel	**-.378**	**.036**
“werk” = work	.190	.306
“werken” = to work	.087	.643
“weten” = to know	-.194	.296
“willen” = to want	-.011	.951
“worden” = to become	-.040	.831
“zeggen” = to say	.284	.121
“zelf” = self	-.345	.057

“zien” = to see	-.199	.283

Notes. Correlations in bold are significant at $p < .05$.

Table S4

Correlations between verbal features and stress at T4

	T4 - SUDS	
	**r**	**p**
"ah" = oh	.054	.775
"beginnen" = to begin	-.146	.433
"denken" = to think	**.389**	**.030**
"eh" = eh	-.169	.364
"fout" = mistake	.041	.828
"he" = hey	-.128	.493
"ja" = yes	-.013	.943
"kijken" = to look	.034	.854
"min" = minus	-.007	.971
"ne" = no	.072	.698
"nee" = no	.147	.430
"neg" = no	-.266	.148
"nou" = well	.206	.266
"oh" = oh	-.045	.811
"oke" = ok	-.261	.156
"oké" = okay	.056	.764
"shit" = shit	-.059	.754
"sorry" = sorry	-.060	.750
"wachten" = to wait	-.083	.658
"worden" = to become	-.190	.306
"zeggen" = to say	-.188	.310

Notes. Correlations in bold are significant at $p < .05$.

Table S5

Correlations between physiological features and stress at T1 through T5

	**T1 - SUDS**		**T2 - SUDS**		**T3 - SUDS**		**T4 - SUDS**		**T5 - SUDS**	
	**r**	**p**	**r**	**p**	**r**	**p**	**r**	**p**	**r**	**p**
**ECG derived variables**										
Average_IBI_msec1	.032	.866	**-.477**	**.007**	-.165	.374	-.029	.878	-.099	.616
Max_IBI_msec1	.049	.799	**-.597**	**.000**	-.164	.377	-.050	.791	-.003	.987
Min_IBI_msec1	.048	.803	**-.404**	**.024**	-.145	.436	-.001	0.994	-.088	.657
RMSSD_msec1	.013	.944	**-.540**	**.002**	-.084	.652	.049	.792	-.041	.837
SDNN_msec1	-.044	.816	**-.515**	**.003**	-.091	.626	.200	.281	-.080	.684
LF_ms1	-.047	.807	**-.577**	**.001**	-.084	.652	.264	.151	-.045	.820
HF_ms1	-.059	.756	**-.474**	**.007**	-.013	.945	.162	.383	-.072	.716
**ICG derived variables**										
Stroke_Volume_(Nederend_2017)_cc1	.082	.668	.047	.801	.267	.146	.164	.379	.084	.673
Minute_Volume_(Nederend_2017)_lmin1	.065	.734	.326	.073	.327	.072	.139	.457	.157	.425
RSA0_msec1	-.045	.815	**-.493**	**.005**	-.067	.722	.177	.342	-.088	.655
Max_RSA0_msec1	-.048	.800	**-.504**	**.004**	-.021	.910	.038	.841	.051	.796
Min_RSA0_msec1	.012	.949	.000	1.000	.000	1.000	.000	1.000	.000	.655
StdDev_RSA0_msec1	-.074	.696	**-.451**	**.011**	.023	.901	.232	.210	-.013	.949
PEP_msec1	-.138	.468	-.350	.054	-.224	.226	-.100	.592	-.188	.338
LVET_msec1	-.057	.763	-.338	.063	-.147	.429	-.130	.484	-.294	.129
TWave_amplitude_mV1	-.058	.762	**-.393**	**.029**	-.321	.079	-.221	.232	.025	.900
Respiration_Rate_bpm1	-.187	.323	.069	.712	-.140	.452	**-.452**	**.011**	.034	.866
Max_RR_bpm1	.075	.692	.157	.400	-.061	.743	-.242	.190	-.017	.931
Min_RR_bpm1	.045	.813	-.002	.992	-.109	.561	**-.418**	**.019**	-.160	.416
StdDev_RR_bpm1	.059	.758	.052	.783	.146	.432	-.350	.054	-.133	.498
Tidal_Volume_mOhm1	.143	.450	.195	.293	.190	.305	.235	.203	.176	.370
Max_Tidal_mOhm1	-.129	.498	.093	.617	.100	.592	.272	.138	.088	.657
Min_Tidal_mOhm1	.002	.992	-.095	.611	.154	.409	.156	.402	.060	.762
StdDev_Tidal_mOhm1	.104	.583	.226	.221	.238	.197	.286	.119	.092	.643
**EDA**										
nsSCRs_per_minute_ppm1	-.026	.891	.189	.310	.036	.846	-.433	.015	-.136	.492
Average_SCL_uS1	.118	.536	.182	.328	.071	.702	.310	.090	.069	.725
Max_SCL_uS1	.167	.379	.251	.173	.102	.584	**.363**	**.045**	.075	.703
Min_SCL_uS1	.059	.758	.164	.377	.054	.772	.207	.263	.112	.571
**Accelerometry derived signal**										
Average_X_Motility_mg1	.199	.292	**.394**	**.028**	**.568**	**.001**	**.386**	**.032**	-.052	.792
Average_Y_Motility_mg1	.279	.135	.192	.302	**.364**	**.044**	.117	.530	.119	.546
Average_Z_Motility_mg1	.228	.225	.093	.618	.064	.733	.309	.091	.101	.610

Notes. Correlations in bold are significant at $p < .05$.

References

Abdulsatar, A. A., Davydov, V., Yushkova, V., Glinushkin, A., & Rud, V. Y. (2019). Age and gender recognition from speech signals. Journal of Physics: Conference Series,

Abhang, P. A., Gawali, B. W., & Mehrotra, S. C. (2016). Technical aspects of brain rhythms and speech parameters. *Introduction to EEG-and speech-based emotion recognition*, 51-79.

Beauchaine, T. P. (2015). Respiratory sinus arrhythmia: A transdiagnostic biomarker of emotion dysregulation and psychopathology. *Current opinion in psychology*, *3*, 43-47.

Beauchaine, T. P., Bell, Z., Knapton, E., McDonough-Caplan, H., Shader, T., & Zisner, A. (2019). Respiratory sinus arrhythmia reactivity across empirically based structural dimensions of psychopathology: A meta-analysis. *Psychophysiology*, *56*(5), e13329.

Berntson, G. G., Cacioppo, J. T., & Quigley, K. S. (1993). Respiratory sinus arrhythmia: Autonomic origins, physiological mechanisms, and psychophysiological implications. *Psychophysiology*, *30*(2), 183-196.

Brindle, R. C., Ginty, A. T., Phillips, A. C., & Carroll, D. (2014). A tale of two mechanisms: A meta-analytic approach toward understanding the autonomic basis of cardiovascular reactivity to acute psychological stress. *Psychophysiology*, *51*(10), 964-976.

Campbell, A. A., Wisco, B. E., Silvia, P. J., & Gay, N. G. (2019). Resting respiratory sinus arrhythmia and posttraumatic stress disorder: A meta-analysis. *Biological Psychology*, *144*, 125-135.

Ciharova, M., Amarti, K., van Breda, W., Peng, X., Lorente-Català, R., Funk, B., Hoogendoorn, M., Koutsouleris, N., Fusar-Poli, P., & Karyotaki, E. (2024). Use of Machine-Learning Algorithms Based on Text, Audio and Video Data in the Prediction of Anxiety and Post-Traumatic Stress in General and Clinical Populations: A Systematic Review. *Biological psychiatry*.

Cornet, V. P., & Holden, R. J. (2018). Systematic review of smartphone-based passive sensing for health and wellbeing. *Journal of biomedical informatics*, *77*, 120-132.

De Geus, E. J., Willemsen, G. H., Klaver, C. H., & van Doornen, L. J. (1995). Ambulatory measurement of respiratory sinus arrhythmia and respiration rate. *Biological Psychology*, *41*(3), 205-227.

Fazeli, S., Levine, L., Beikzadeh, M., Mirzasoleiman, B., Zadeh, B., Peris, T., & Sarrafzadeh, M. (2023). A Self-supervised Framework for Improved Data-Driven Monitoring of Stress via Multi-modal Passive Sensing. 2023 IEEE International Conference on Digital Health (ICDH),

Ferrand, C. T. (2002). Harmonics-to-noise ratio: an index of vocal aging. *JOURNAL OF VOICE*, *16*(4), 480-487.

Fleischer, M., Pinkert, S., Mattheus, W., Mainka, A., & Mürbe, D. (2015). Formant frequencies and bandwidths of the vocal tract transfer function are affected by the mechanical impedance of the vocal tract wall. *Biomechanics and modeling in mechanobiology*, *14*, 719-733.

Giannakopoulos, T., & Pikrakis, A. (2014). Chapter 4-audio features. *Introduction to audio analysis*, 59-103.

Goedhart, A. D., Van Der Sluis, S., Houtveen, J. H., Willemsen, G., & De Geus, E. J. (2007). Comparison of time and frequency domain measures of RSA in ambulatory recordings. *Psychophysiology*, *44*(2), 203-215.

Katona, P. G., & Jih, F. (1975). Respiratory sinus arrhythmia: noninvasive measure of parasympathetic cardiac control. *Journal of applied physiology*, *39*(5), 801-805.

Kelsey, R. M. (1991). Electrodermal lability and myocardial reactivity to stress. *Psychophysiology*, *28*(6), 619-631.

Kubicek, W. (1966). Development and evaluation of an impedance cardiac output system. *Aerosp. Med.*, *37*, 1208-1212.
Migliaro, E. R. (2020). The mechanical side of respiratory sinus arrhythmia. *Physiological Mini Reviews*, *13*.
Miller, L. H., & Shmavonian, B. M. (1965). Replicability of two GSR indices as a function of stress and cognitive activity. *Journal of personality and social psychology*, *2*(5), 753.
Nederend, I., de Geus, E. J., Kroft, L. J., Westenberg, J. J., Blom, N. A., & Ten Harkel, A. D. (2018). Cardiac autonomic nervous system activity and cardiac function in children after coarctation repair. *The Annals of Thoracic Surgery*, *105*(6), 1803-1808.
Nikula, R. (1991). Psychological correlates of nonspecific skin conductance responses. *Psychophysiology*, *28*(1), 86-90.
Oudin, A., Maatoug, R., Bourla, A., Ferreri, F., Bonnot, O., Millet, B., Schoeller, F., Mouchabac, S., & Adrien, V. (2023). Digital phenotyping: Data-driven psychiatry to redefine mental health. *Journal of medical Internet research*, *25*, e44502.
Rahman, S., Habel, M., & Contrada, R. J. (2018). Poincaré plot indices as measures of sympathetic cardiac regulation: Responses to psychological stress and associations with pre-ejection period. *International Journal of Psychophysiology*, *133*, 79-90.
Riese, H., Groot, P. F., van den Berg, M., Kupper, N. H., Magnee, E. H., Rohaan, E. J., Vrijkotte, T. G., Willemsen, G., & de Geus, E. J. (2003). Large-scale ensemble averaging of ambulatory impedance cardiograms. *Behavior Research Methods, Instruments, & Computers*, *35*, 467-477.
Salehi, A. W., Baglat, P., & Gupta, G. (2020). Review on machine and deep learning models for the detection and prediction of Coronavirus. *Materials Today: Proceedings*, *33*, 3896-3901.
Schultebraucks, K., Yadav, V., Shalev, A. Y., Bonanno, G. A., & Galatzer-Levy, I. R. (2022). Deep learning-based classification of posttraumatic stress disorder and depression following trauma utilizing visual and auditory markers of arousal and mood. *Psychological Medicine*, *52*(5), 957-967.
Shiffman, S. (2009). Ecological momentary assessment (EMA) in studies of substance use. *Psychological assessment*, *21*(4), 486.
Sueur, J., & Sueur, J. (2018). Mel-frequency cepstral and linear predictive coefficients. *Sound Analysis and Synthesis with R*, 381-398.
Thompson, W., Peter, V., Olsen, K. N., & Stevens, C. J. (2012). The effect of intensity on relative pitch. *Quarterly Journal of Experimental Psychology*, *65*(10), 2054-2072.
Tonhajzerova, I., Mestanik, M., Mestanikova, A., & Jurko, A. (2016). Respiratory sinus arrhythmia as a non-invasive index of 'brain-heart'interaction in stress. *The Indian journal of medical research*, *144*(6), 815.
Torous, J., Kiang, M. V., Lorme, J., & Onnela, J.-P. (2016). New tools for new research in psychiatry: a scalable and customizable platform to empower data driven smartphone research. *JMIR mental health*, *3*(2), e5165.
van der Mee, D., Duivestein, Q., Gevonden, M., Westerink, J., & de Geus, E. (2020). The short Sing-a-Song Stress Test: A practical and valid test of autonomic responses induced by social-evaluative stress. *Autonomic Neuroscience*, *224*, 102612.
van der Mee, D., Gevonden, M., Westerink, J. H., & De Geus, E. (2021). Validity of electrodermal activity-based measures of sympathetic nervous system activity from a wrist-worn device. *International Journal of Psychophysiology*, *168*, 52-64.
Wang, L., Hu, Y., Jiang, N., & Yetisen, A. K. (2024). Biosensors for psychiatric biomarkers in mental health monitoring. *Biosensors and Bioelectronics*, 116242.

Willemsen, G. H., DeGeus, E. J., Klaver, C. H., VanDoornen, L. J., & Carrofl, D. (1996). Ambulatory monitoring of the impedance cardiogram. *Psychophysiology*, *33*(2), 184-193.
